# Modular dual-color BiAD sensors for locus-specific readout of epigenome modifications in single cells

**DOI:** 10.1016/j.crmeth.2024.100739

**Published:** 2024-03-29

**Authors:** Anja R. Köhler, Johannes Haußer, Annika Harsch, Steffen Bernhardt, Lilia Häußermann, Lisa-Marie Brenner, Cristiana Lungu, Monilola A. Olayioye, Pavel Bashtrykov, Albert Jeltsch

**Affiliations:** 1 Institute of Biochemistry and Technical Biochemistry, University of Stuttgart, Allmandring 31, 70569 Stuttgart, Germany; 2 Institute of Cell Biology and Immunology, University of Stuttgart, Allmandring 31, 70569 Stuttgart, Germany

**Keywords:** epigenome modification, fluorescence microscopy, histone methylation, DNA methylation, split fluorophore, bimolecular fluorescence complementation, single-cell analysis

## Abstract

Dynamic changes in the epigenome at defined genomic loci play crucial roles during cellular differentiation and disease development. Here, we developed dual-color bimolecular anchor detector (BiAD) sensors for high-sensitivity readout of locus-specific epigenome modifications by fluorescence microscopy. Our BiAD sensors comprise an sgRNA/dCas9 complex as anchor and double chromatin reader domains as detector modules, both fused to complementary parts of a split IFP2.0 fluorophore, enabling its reconstitution upon binding of both parts in close proximity. In addition, a YPet fluorophore is recruited to the sgRNA to mark the genomic locus of interest. With these dual-color BiAD sensors, we detected H3K9me2/3 and DNA methylation and their dynamic changes upon RNAi or inhibitor treatment with high sensitivity at endogenous genomic regions. Furthermore, we showcased locus-specific H3K36me2/3 readout as well as H3K27me3 and H3K9me2/3 enrichment on the inactive X chromosome, highlighting the broad applicability of our dual-color BiAD sensors for single-cell epigenome studies.

## Highlights

• Dual-color BiAD sensors combine CRISPR imaging with epigenome modification readout
• Locus-specific detection of DNA, H3K9, H3K27, and H3K36 methylation in live cells
• Quantitative analysis of epigenome dynamics upon DNMT inhibitor treatment
• Investigation of cellular effects of H3K9 methyltransferase knockdown

## Motivation

Bimolecular anchor detector (BiAD) sensors enable the detection of epigenome modifications with locus-specific resolution in live cells. However, their application in human cells has so far been limited to DNA methylation readout at highly repetitive genomic regions. To improve the sensitivity, we aimed to develop dual-color BiAD sensors by combining CRISPR imaging for visualization of the specific genomic target locus with the BiAD readout of the epigenome modifications at this site. Thus, the BiAD analysis could be focused on the target locus region, allowing us to disregard signals in other parts of the nucleus, which was expected to increase the sensitivity of the method strongly. Moreover, we aimed to integrate additional double chromatin reader domains as detector modules into the system to increase its applicability for the readout of important histone posttranslational modifications in human cells.

## Graphical abstract

**Figure:**
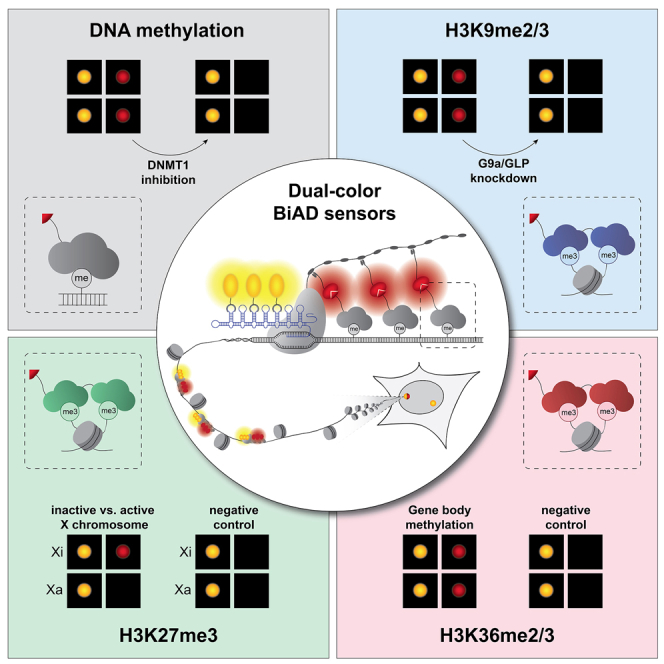

## Teaser

Bimolecular anchor detector (BiAD) sensors enable locus-specific investigation of epigenome modifications in live cells. Köhler et al. develop dual-color BiAD sensors combining the BiAD detection with CRISPR imaging for improved sensitivity and introduce detectors for dynamic readout of DNA, H3K9, H3K27, and H3K36 methylation at human endogenous target loci.

## Introduction

The dynamic regulation of gene expression during embryonic development and cellular differentiation is governed by a complex network of epigenetic mechanisms.^1^^,^^2^^,^^3^ All of these processes require the precise spatiotemporal establishment and maintenance of defined patterns of epigenome modifications, such as DNA methylation and numerous histone posttranslational modifications (PTMs).^4^^,^^5^^,^^6^ Conversely, disruptions in these locus-specific epigenomic patterns have been linked to the onset and progression of a wide spectrum of human diseases, including cancer.^7^^,^^8^^,^^9^^,^^10^ Adding to the complexity, it has become increasingly evident that many physiological and pathophysiological processes exhibit a high cell-to-cell heterogeneity.^11^^,^^12^^,^^13^ Therefore, gaining a comprehensive understanding of the locus-specific dynamics of epigenome modifications at single-cell resolution is essential to unravel the regulatory mechanisms underlying these processes, connect them to cellular phenotypes, and develop effective treatment strategies.^13^^,^^14^

Recent advancements in epigenomic sequencing technologies, such as DNA methylation analysis by bisulfite conversion coupled to sequencing and analysis of histone PTMs by various types of chromatin immunoprecipitation followed by high-troughput sequencing (ChIP-seq), including single-cell approaches, have significantly enhanced our understanding of the epigenome in both health and disease.^15^^,^^16^ However, these techniques can only provide a snapshot of the epigenomic landscape at a particular time point, and they cannot be used to match epigenome modifications and cellular phenotypes because the procedures require cell lysis. To address this limitation, bimolecular anchor detector (BiAD) sensors were developed for locus-specific readout of epigenome modifications in single cells.^17^ The BiAD technology is also compatible with live-cell imaging, potentially allowing the analysis of dynamic changes of epigenome modifications at the level of individual cells. The BiAD sensors comprise a programmable DNA-binding anchor module, such as engineered zinc finger proteins, transcription activator-like effectors, or a single-guide RNA (sgRNA)/dCas9 (dead Cas9) complex for locus-specific targeting of the sensor, and different chromatin reader domains used as detector modules for the recognition of defined chromatin modifications. Both modules are fused to complementary parts of a split fluorophore, enabling bimolecular fluorescence complementation (BiFC) when both modules bind in close spatial proximity. However, the imaging-based readout requires a strong BiFC signal at the target locus to distinguish it from the nuclear background, fluorophore aggregation, and signal fluctuations. Hence, the application of BiAD sensors so far was limited to highly repetitive genomic loci with >1,000 local repeats,^17^ which lead to the binding of several BiAD sensors at the target locus. Thus, enhancing the sensitivity of the BiAD sensors was essential to overcome this limitation and enable their application to a broader range of biologically relevant endogenous target regions.

Over the last years, several dCas9-based signal amplification systems have been developed for the visualization of endogenous genomic loci in live cells. This can be achieved by using a dCas9-fused peptide scaffold, like the SunTag, which enables the binding of multiple antibody-fused fluorophores per sgRNA/dCas9 binding site.^18^ Another frequently used method is to employ modified sgRNAs, such as MS2-based amplification systems^19^^,^^20^ or the Casilio system,^21^^,^^22^ which specifically recruit multiple fluorescently labeled RNA-binding proteins to the sgRNA/dCas9 target locus. Although these systems are already applied in CRISPR imaging for locus-specific genomic visualization of repeats with lower local copy numbers and, in some cases, even at nonrepetitive loci, they do not provide information on the epigenomic state at the target locus.

In this study, we combined CRISPR imaging for the visualization of specific genomic loci with the BiAD sensor-based readout of chromatin modifications at the endogenous target sites, using two distinct sgRNA/dCas9-based signal amplification systems. This dual-color readout drastically enhanced the sensitivity of BiAD sensors, enabling the qualitative and quantitative locus-specific detection of chromatin modifications at regions with low local copy numbers in single cells by fluorescence microscopy. Furthermore, we integrated improved detector modules for different epigenome modifications by using double chromatin reader domains for readout of histone 3 lysine 9 di- and trimethylation (H3K9me2/3), H3K27me3, and H3K36me2/3 in human cells, in addition to the already-established detector module for DNA methylation based on the MBD domain of MBD1. With its improved sensitivity and diverse panel of detector modules, these modular dual-color BiAD sensors offer a versatile and powerful tool to investigate locus-specific changes in chromatin modifications at a broad range of endogenous target sites in live cells. Our sensors allow the readout of epigenome modifications to be directly coupled with cellular phenotypes and cell physiology. These new developments are expected to provide important insights into epigenomic signaling pathways at the single-cell level.

## Results

In our previous work, different BiAD sensors were established for locus-specific detection of epigenome modifications in living cells.^17^ This included dCas9-based BiAD sensors for DNA cytosine-C5 methylation (5mC) readout, using the N-terminal part of split mVenus fused to the dCas9 anchor module and the C-terminal part of mVenus fused to the MBD domain of MBD1. This enabled BiFC when both modules bound to chromatin in close spatial proximity. The design of these sensors allowed for the reconstitution of one mVenus fluorophore per dCas9 binding site, if 5mC was present at the target locus. Although this approach was successfully applied for the detection of 5mC at highly repetitive centromeric and pericentromeric sequences in mouse and human cells with >1,000 local repeats, preliminary work in our lab showed that it was not functional at target loci with a smaller number of local repeats because fewer reconstituted fluorophores made it difficult to detect the BiFC signal.

### Dual-color BiAD sensor improves sensitivity of 5mC detector

**Figure 1:**
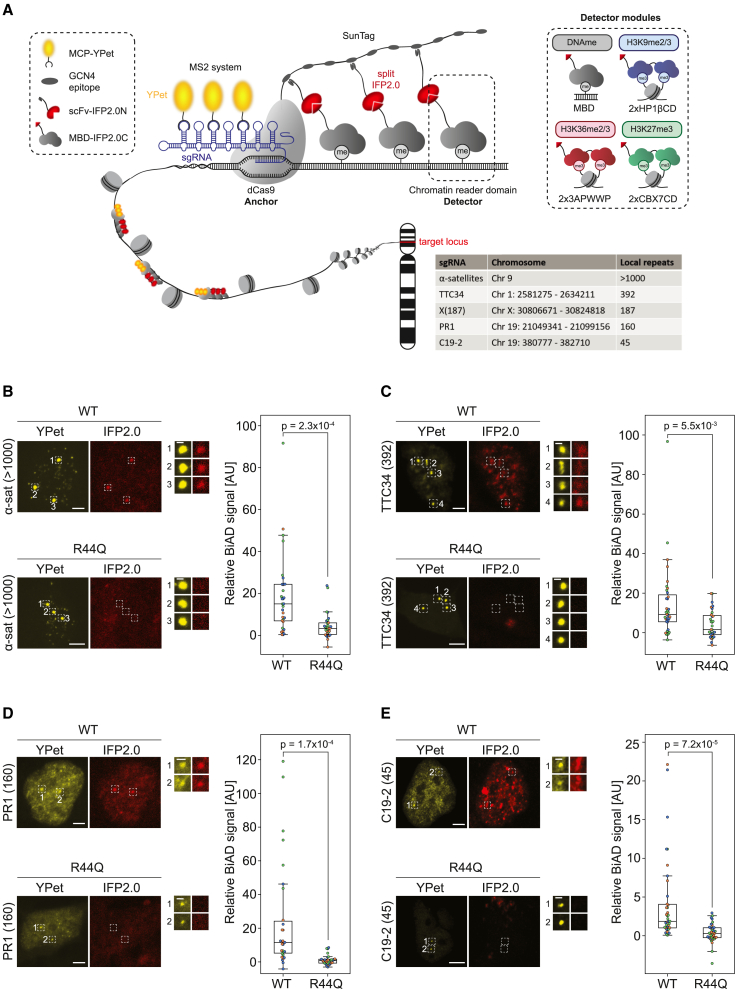
Validation of the dual-color BiAD sensor for 5mC readout (A) Schematic representation of the modular dual-color BiAD sensor and compilation of selected target loci with decreasing local repeat copy numbers. The target locus is visualized by a full-length fluorophore (YPet), which is recruited by an MS2 scaffold of the sgRNA in complex with the anchor module (dCas9). One part of the split fluorophore (IFP2.0) is recruited to the SunTag amplification system fused to dCas9 by an scFv antibody. If the chromatin modification of interest is present at the target locus, then the detector module (chromatin reader domain) can bind and bring the second part of the split IFP2.0 in close spatial proximity, leading to reconstitution of the split IFP2.0. Detector modules used for the recognition of DNA methylation and histone posttranslational modifications were the MBD1 MBD domain for DNA methylation and double domains (2×) of the HP1β CD for H3K9me2/3, DNMT3A PWWP domain for H3K36me2/3, and CBX7CD for H3K27me3. (B–E) HEK293 cells were transfected with all components of the dual-color BiAD sensor for 5mC detection with either the WT MBD1 detector or a 5mC binding-deficient mutant (R44Q). The BiAD sensor was tested on different target loci with decreasing local repeat numbers: (B) α-satellites (>1,000 repeats), (C) TTC34 (392 repeats), (D) PR1 (160 repeats), (E) and C19-2 (45 repeats). Exemplary fluorescence microscopy images showing the nucleus of individual fixed cells with colocalization of the marker (YPet) and BiAD signal (IFP2.0) for the WT detector, but not for the binding-deficient R44Q mutant. Scale bars, 5 μm and 1 μm for the magnified images. The boxplots show the relative BiAD signals. Each dot represents the mean relative BiAD signal of all of the spots within a single cell. Data were obtained in 3 independent experiments (depicted in orange, blue, and green). Significance was determined via a 2-tailed, unpaired t test. The p values are indicated in each boxplot. See also Figure S2.

To enhance the sensitivity of the 5mC BiAD sensor for readout of epigenome modifications at repeat loci with lower local copy numbers, we first aimed to amplify the fluorescent signal. For this, we added a 10×SunTag to the dCas9 and fused the N-terminal part of split mVenus to a single-chain fragment variable (scFv) antibody fragment that specifically binds the GCN4 epitopes of the SunTag. This system enables the recruitment of multiple detector modules to a single sgRNA/dCas9 binding site, because in principle each N-terminal mVenus fragment can reconstitute a full fluorophore. The performance of the improved BiAD sensor was analyzed using a calibration set of genomic loci containing repeats with different local copy numbers between 45 and >1,000 taken from Ma et al.^20^ (Figure 1A). However, the resulting signal amplification was still insufficient to reliably discriminate BiFC signals at target regions with <1,000 repeats from an unspecific nuclear background, like fluorophore aggregation or noise (Figure S1A). To overcome this limitation, we explored the possibility of using an additional detection system to recruit a full fluorophore to the same place and by this visually mark the target locus and then allow specific readout of the BiAD signal only at this region. Using the CRISPR-Sirius MS2-based amplification system,^20^ we could reliably detect regions with as few as 45 local repeats in HEK293 cells using the bright YPet fluorophore^23^ as detector (Figure S1B). Depending on the target locus, we observed two to four distinct YPet spots, which in each case was in line with the known polyploidy of HEK293 cells.^24^

After evaluating several split fluorophores, we chose the near-infrared fluorophore IFP2.0 because it was previously used for reversible BiFC assays,^25^^,^^26^ and it does not overlap spectrally with the YPet marker signal. IFP2.0 was split at position 132 to create two nonfluorescent parts. The N-terminal part was fused to the scFv antibody fragment binding to the SunTag, and the C-terminal part was fused to the detector module. This dual-color BiAD sensor combines the visualization of a specific genomic locus in the YPet channel with the BiAD sensor-based readout of chromatin modifications at the target locus in the IFP2.0 channel (Figure 1A). Moreover, the YPet marker fluorophore not only enables the precise localization of the dCas9 binding site but it also serves as an independent normalization marker for size, condensation, and accessibility of the target locus facilitating the quantitative analysis of the IFP2.0 BiAD signal at the target locus (Figure S1C).

We first applied the sensor with the MBD1 detector module for the investigation of DNA methylation at the genomic loci from the calibration set, revealing colocalization of the YPet signal with the reconstituted IFP2.0 BiAD signal for the wild-type (WT) MBD1 detector, but not for the 5mC binding-deficient R44Q mutant, which was used as a negative control.^17^^,^^27^ Control experiments lacking the scFv-IFP2.0N component did not reveal noticeable BiAD signals, confirming the specificity of our sensor (Figure S2A). With this dual-color BiAD sensor, we successfully detected 5mC at loci with decreasing local copy numbers, ranging from highly repetitive α-satellites (>1,000 repeats) to as few as 45 repeats at the C19-2 locus, indicating a drastic increase in sensitivity (Figures 1B–1E). In particular, in the case of the low copy-number repeats, based on the IFP2.0 BiAD signal alone, it was not possible to distinguish the signal at the locus of interest from unspecific nuclear background signals, emphasizing the crucial need for a second, independent fluorophore marking the endogenous target site that is a key advantage of our dual-color BiAD sensors. Since we did not observe adverse effects of cellular fixation on the BiAD and YPet signals (Figure 1D) compared to live-cell imaging as exemplarily shown for the PR1 (160 repeats) locus (Figure S2B), we proceeded with fixation of the transfected cells for more convenient handling and more flexible experimental design and allocation of instrument time at the microscope, and to allow direct comparison of BiAD images with immunofluorescence analyses.

For the quantitative analysis of the BiAD signal at the target locus, two intensity thresholds in the YPet channel were used—one to define the nucleus and the other to define high-intensity YPet spots as regions of interest (ROIs). Subsequently, the mean signal intensities in the YPet and IFP2.0 channels were measured for nucleus and spot ROIs. After subtracting the respective mean nuclear intensities, the IFP2.0 BiAD spot intensity was normalized to the corresponding YPet spot intensity. The resulting normalized BiAD signal was averaged for all of the spots within a single cell (for a detailed explanation, refer to the STAR Methods section and Figure S1C). The analysis of at least 30 cells with WT and R44Q negative control detector revealed a highly significant number of WT cells with a strong BiAD signal for all of the tested target loci (Figures 1B–1E). An exemplary analysis of the PR1 (160 repeats) locus data did not show noticeable differences in the YPet spot area or the spot count per nucleus for cells transfected with WT or R44Q detector (Figures S2C and S2D). For the purposes of this study, we deliberately chose to use transient transfections because they facilitate the flexible interchange of different modules to adjust to various experimental setups. However, it must be noted that cells had to be transiently transfected with five plasmids encoding all of the components of the dual-color BiAD sensor, but selection for imaging and analysis was based only on the presence of YPet spots. Defined YPet spots indicated the successful transfection and expression of three components: dCas9-SunTag, MCP-YPet, and the sgRNA, whereas the detector and the scFv modules, which carry the nonfluorescent IFP2.0 parts, cannot be accounted for. Hence, lack of sufficient amounts of these plasmids in some of the transfected cells may explain the occurrence of cells with no apparent BiAD signal even in settings where positive BiAD signals are expected. Therefore, the potential presence of cells incapable of generating a BiAD signal must be considered for the interpretation of all of the results.

### Detection of dynamic changes of DNA methylation in living cells with the dual-color BiAD sensor

**Figure 2:**
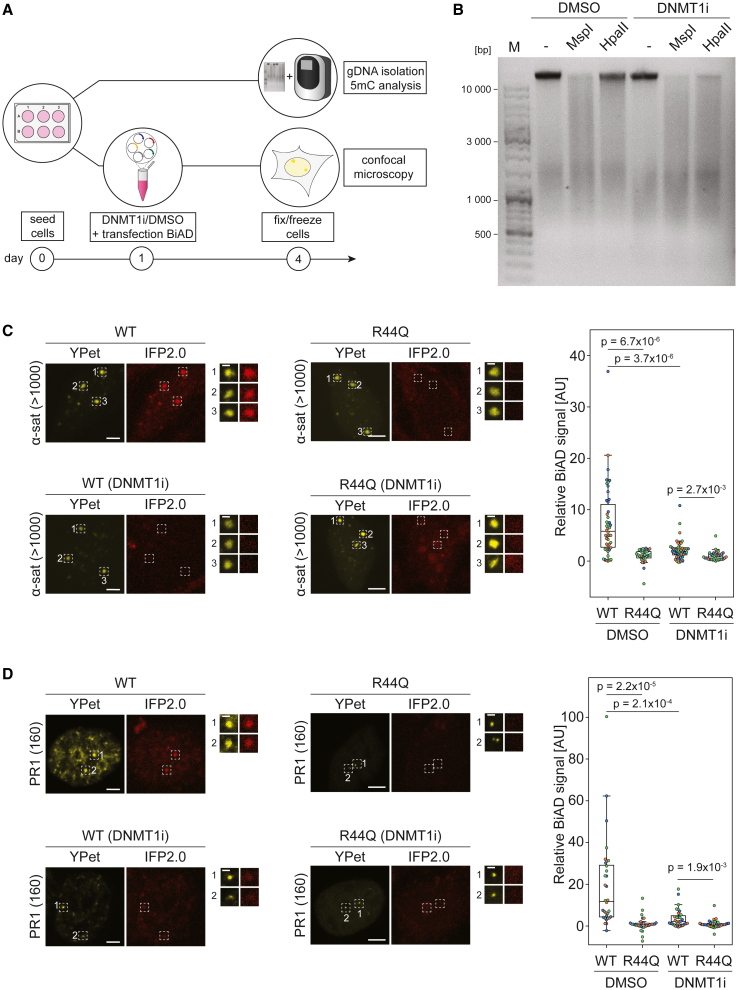
Visualization of locus-specific 5mC loss upon GSK-3685032 DNMT1i treatment (A) Schematic representation of the experimental setup. HEK293 cells were transfected with all components of the dual-color BiAD sensor for 5mC detection with either the WT MBD1 detector or a binding-deficient mutant (R44Q). Starting at the day of transfection, cells were treated with the DNMT1i or mock treated (DMSO) for 3 days. (B) Agarose gel showing global loss of CpG methylation in genomic DNA upon DNMT1i treatment. Equal amounts of genomic DNA isolated from DNMT1i/DMSO-treated cells were digested at CCGG sites with MspI (5mCpG insensitive) or HpaI (5mCpG sensitive). (C and D) Representative fluorescence microscopy images of fixed cells showing the loss of 5mC BiAD signal at (C) α-satellites (>1,000 repeats) and (D) PR1 (160 repeats) upon DNMT1i treatment. Scale bars, 5 μm and 1 μm for the magnified images. Boxplots show the relative BiAD signal intensities of DNMT1i- and mock-treated cells normalized to the respective MBD1 R44Q mutant. Data were obtained in 3 independent experiments (depicted in orange, blue, and green). Significance was determined via a 2-tailed, unpaired t test. The p values are indicated in each boxplot. See also Figure S2.

To determine whether the dual-color BiAD sensor can be used to track locus-specific dynamic changes in 5mC levels, we investigated 5mC in HEK293 cells upon treatment with the GSK-3685032 DNA methyltransferase 1 inhibitor (DNMT1i).^28^ Therefore, we transfected HEK293 cells with all of the components of the dual-color BiAD sensor for 5mC readout and treated them either with GSK-3685032 DNMT1i or with DMSO (mock control) (Figure 2A). Three days after treatment, global DNA demethylation at CpG sites in the DNMT1i-treated cells was verified by MspI (5mCpG insensitive) and HpaII (5mCpG sensitive) restriction digestion of purified genomic DNA (Figure 2B). Moreover, demethylation at the PR1 locus was confirmed by restriction digestion analyzed by qPCR at two exemplary sites (Figure S2E). Subsequently, using the dual-color BiAD sensors to assess locus-specific 5mC changes, we observed drastically reduced BiAD signals at α-satellite (>1,000 repeats) and PR1 (160 repeats) regions after 3 days of inhibitor treatment in comparison to mock-treated cells (Figures 2C and 2D). These findings document the applicability of the dual-color BiAD 5mC sensor to monitor changes in DNA methylation levels at endogenous genomic loci upon DNMT1i treatment by fluorescence microscopy in single cells.

### Double detector domains enhance the detection of histone PTMs in cells

In addition to the DNA methylation readout, our goal was to broaden the range of detector modules and allow the investigation of additional histone PTMs with our dual-color BiAD sensors. Previously, we used the chromodomain (CD) of HP1β (CBX1) for detection of H3K9me2/3 at mouse major satellite repeats.^17^ However, this detector displayed low sensitivity in human cell lines. Based on recent studies and our own experience,^29^^,^^30^^,^^31^^,^^32^^,^^33^ we hypothesized that the binding affinity and specificity of the detector modules toward histone PTMs could be improved by using two fused domains that could benefit from avidity effects in their specific chromatin interaction with two modified histone tails at the target locus (called double domains henceforth). We, therefore, evaluated single and double domains (2×) of HP1βCD for H3K9me2/3, the PWWP domain of DNMT3A (3APWWP) for H3K36me2/3 and the CBX7 chromodomain (CBX7CD) for H3K27me3 readout.

**Figure 3:**
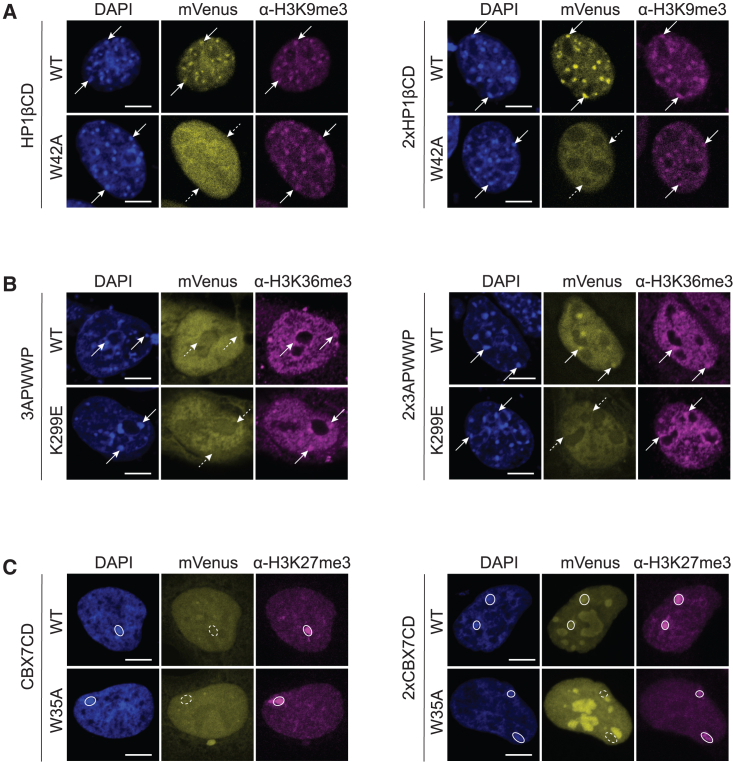
Validation of single and double reader domains as detector modules for histone PTMs (A) Representative fluorescence microscopy images of fixed NIH3T3 cells transfected with mVenus-fused HP1βCD single or double domain (2×) and the corresponding W42A binding pocket mutants. The WT 2×HP1βCD colocalizes with H3K9me3 staining at DAPI-dense heterochromatic regions, as exemplarily indicated by arrows. Dotted arrows indicate missing colocalization of the respective W42A mutants. (B) Representative fluorescence microscopy images of fixed NIH3T3 cells transfected with mVenus-fused DNMT3A PWWP single (3APWWP) or double domain (2×) and the corresponding K299E binding pocket mutants. The WT 2×3APWWP domain colocalizes with H3K36me3 immunostaining at DAPI-dense heterochromatic regions, as exemplarily indicated by arrows. Dotted arrows indicate missing colocalization of the single domain and the respective K299E mutants. (C) Representative fluorescence microscopy images of fixed HEK293 cells transfected with mVenus-fused CBX7CD single or double domain (2×) and the corresponding binding-deficient W35A mutants. 2×CBX7CD WT colocalizes with H3K27me3 and DAPI staining at the Xi, as indicated by circles. Dotted circles indicate missing colocalization of the single domain and the respective W35A mutants. In all of the panels, the images are representative of 5–15 individual cells that were analyzed. See also Figure S3.

Pilot experiments to assess the detection of the H3K9me2/3 and H3K36me2/3 modifications were conducted in NIH3T3 cells because immunofluorescence staining for both histone PTMs displayed distinct subnuclear patterns in these cells. Comparison of NIH3T3 cells transfected with single and double (2×) HP1βCD domains fused to mVenus revealed the previously observed heterochromatic pattern of the mVenus signal colocalizing with DAPI and H3K9me3 staining in mouse cells for the WT reader domain.^17^ However, the double domain displayed an even more pronounced spotty phenotype, indicating enhanced H3K9me2/3 binding (Figure 3A). Cells transfected with the binding pocket mutant W42A did not display a spotty pattern for both single and double domains. To validate these findings, we performed cotransfections of WT and W42A double domains in NIH3T3 as well as HEK293 cells (Figure S3A). By fusing WT and mutant double domains to different fluorophores (WT-mVenus/W42A-dsRed), we could observe their respective localization at the same time within the same cell. To exclude an adverse effect of the fused fluorophore on the binding of the detector modules, we first confirmed colocalization of WT-mVenus and WT-dsRed double domains with H3K9me3-dense, heterochromatic foci in the same cell. However, this pattern was not detected for cotransfections of WT-mVenus with W42A-dsRed, thus confirming that the specific H3K9me2/3 binding was lost with the W42A mutant.

For the 3APWWP domain, we once again observed that the double domain provided an advantage for H3K36me2/3 detection because it showed colocalization with H3K36me3 antibody-stained heterochromatic foci in NIH3T3, whereas the single domain did not (Figure 3B). The binding-deficient K299E mutant^34^ did not display colocalization with H3K36me3 or DAPI staining. These results were confirmed by cotransfection experiments with 2×3APWWP (WT-mVenus/dsRed and WT-mVenus/K299E-dsRed) in the same cell (Figure S3B).

Evaluation of CBX7CD single and double domain binding was carried out in HEK293 cells, which exhibit robust H3K27me3 staining at the inactive X chromosome(s) (Xi). For H3K27me3 detection, transfections with single CBX7CD revealed a diffuse subnuclear signal with no visible colocalization with the H3K27me3 immunostaining, whereas the double domain (2×CBX7CD) clearly colocalized with Xi based on the H3K27me3 staining (Figure 3C). The binding-deficient W35A mutant^35^ did not show this colocalization. This specific loss of H3K27me3 binding by the W35A mutant was also validated by cotransfection experiments (Figure S3C). Since previous *in vitro* studies reported binding of the CBX7CD to H3K27me3 as well as H3K9me3,^36^ we cotransfected 2×HP1βCD and 2×CBX7CD into NIH3T3 and HEK293 cells and conducted immunostaining for H3K9me3 and H3K27me3 (Figure S3D). The Xi in HEK293 cells showed strong H3K9me3 and H3K27me3 staining, which was reflected by colocalization with 2×HP1βCD as well as 2×CBX7CD. In contrast, the H3K9me3-dense foci in NIH3T3 and HEK293 cells did not colocalize with 2×CBX7CD-mVenus, indicating preferred binding of 2×CBX7CD to H3K27me3 in the cellular context.^31^ These initial experiments indicated that the use of double domains, which enable multivalent interactions with the chromatin at the target site, improved the recognition of histone PTMs in all three cases.

### Validation of additional detector modules for locus-specific H3K27me3 and H3K36me2/3 readout in dual-color BiAD sensors

**Figure 4:**
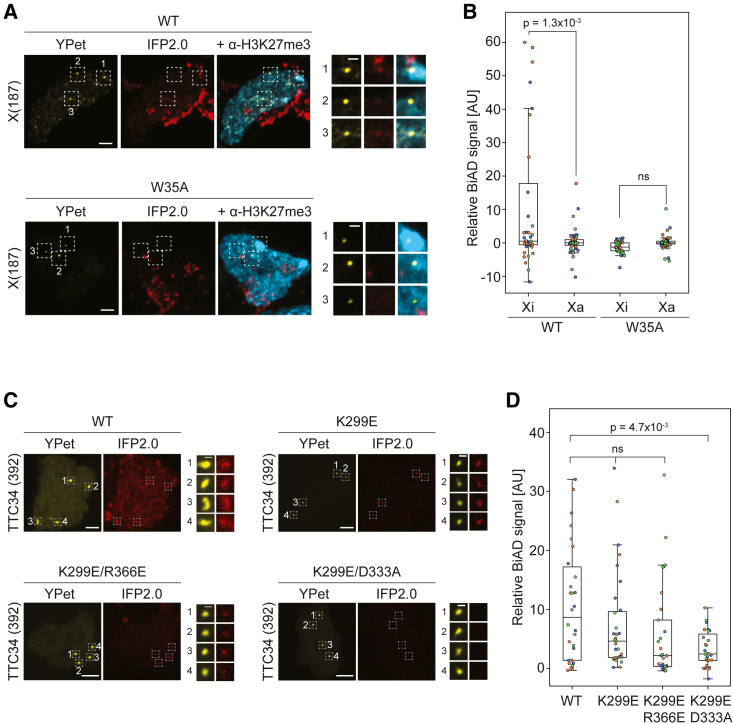
Validation of dual-color BiAD sensors for H3K27me3 and H3K36me2/3 readout (A and B) HEK293 cells were transfected with all components of the dual-color BiAD sensor for H3K27me3 detection at the X(187) locus using either the WT 2×CBX7CD detector or a binding-deficient W35A mutant. Cells were immunostained for H3K27me3 to identify the inactive (Xi) and active (Xa) X chromosomes. (A) Representative fluorescence microscopy images of fixed cells showing the colocalization of the marker (YPet) and BiAD signals (IFP2.0) for the WT detector at the Xi (spot 1), which was not observed at the Xa (spots 2 and 3), or for transfections with the W35A mutant. The H3K27me3 immunostaining signal is shown in cyan. Scale bars, 5 μm and 1 μm for the magnified images. (B) Boxplots showing the BiAD signal (IFP2.0) relative to the marker signal (YPet). Individual spots were separated into Xi and Xa and normalized to the mean Xa signal for each cell. Data were obtained in 3 independent experiments (depicted in orange, blue, and green). Significance was determined via a 2-tailed, unpaired t test. The p values are indicated in the boxplot; ns, nonsignificant p values (p > 0.05). (C and D) HEK293 cells were transfected with all of the components of the dual-color BiAD sensor for H3K36me2/3 detection at the TTC34 locus with either the WT 2×3APWWP detector or K299E, K299E/R366A, and K299E/D333A binding pocket mutants. (C) Exemplary fluorescence microscopy images of fixed cells showing the colocalization of the marker (YPet) and BiAD signal (IFP2.0) for the WT detector as well as the K299E and K299E/R366A mutants. No colocalization was observed for the K299E/D333A mutant, indicating that it is a suitable negative control. Scale bars, 5 μm and 1 μm for the magnified images. (D) Boxplots showing the relative BiAD intensities. Each dot represents the mean relative BiAD signal of all of the spots within a single cell. Data were obtained in 3 independent experiments (depicted in orange, blue, and green). Significance was determined via a 1-way ANOVA with Tukey’s posttest. The p values are indicated in the boxplot; ns, nonsignificant p values (p > 0.05).

To assess the potential of the 2×CBX7CD detector module as dual-color BiAD sensor for H3K27me3 readout, we selected the X(187) region on the X chromosome with 187 local repeats, since the Xi in female cells is characterized by enriched H3K27me3.^37^ In our preliminary experiments (Figure 3C), we observed that the Xi forms a visible Barr body in most cells, which is characterized by dense DAPI staining. However, many other heterochromatic regions also display elevated DAPI signals, which is why we chose to use H3K27me3 staining for identification of the Xi in our experiments to clearly distinguish it from other heterochromatic regions. Therefore, we additionally immunostained cells with an H3K27me3 antibody to identify the inactive (Xi) X chromosome(s). The sensor typically detected three spots in HEK293 cells in the YPet channel, which agrees with the polyploidy of this cell line. In most cells, one or two of the spots overlapped with H3K27me3 staining, indicating that these loci represent an Xi. Comparison of the relative BiAD IFP2.0 signals obtained with the WT 2×CBX7CD detector revealed a stronger signal on the Xi relative to the Xa (Figures 4A and 4B). This indicates that the dual-color BiAD sensor successfully detects the enrichment of H3K27me3 at the Xi. In contrast, the binding-deficient detector module 2×CBX7CD W35A did not show any BiAD signal, further confirming the specificity of the sensor for H3K27me3 detection.

To assess the potential of 2×3APWWP as the dual-color BiAD detector module, a repetitive region in a gene body (TTC34, 392 repeats) was selected because gene bodies of expressed genes are known to carry extensive H3K36me3.^38^ Transfections of the dual-color BiAD sensor for H3K36me2/3 readout at the TTC34 locus with the 2×3APWWP detector module revealed colocalization of the YPet marker signal and the BiAD IFP2.0 signal at the target locus in HEK293 cells (Figures 4C and 4D). However, we still observed some cells with an IFP2.0 signal at the target locus with the K299E binding pocket mutant, indicating insufficient performance of the negative control most likely due to residual binding of the mutated detector module. To address this issue, we tested two double mutants of the detector domain, K299E/D333A and K299E/R366E, because previous work from our laboratory showed the importance of the two residues D333 and R366 for H3K36me2/3 and DNA binding, respectively.^34^ Indeed, the K299E/D333A double mutant effectively eliminated the unspecific IFP2.0 signal, indicating its attribution to residual H3K36me2/3 rather than DNA binding (Figures 4C and 4D). These results demonstrate that the 2×CBX7CD and 2×3APWWP domains and their respective binding-deficient mutants can be used as detector modules in dual-color BiAD sensors to visualize H3K27me3 and H3K36me2/3 at endogenous target sites in live cells.

### Detection of H3K9me3 on Xi using the dual-color BiAD system

**Figure 5:**
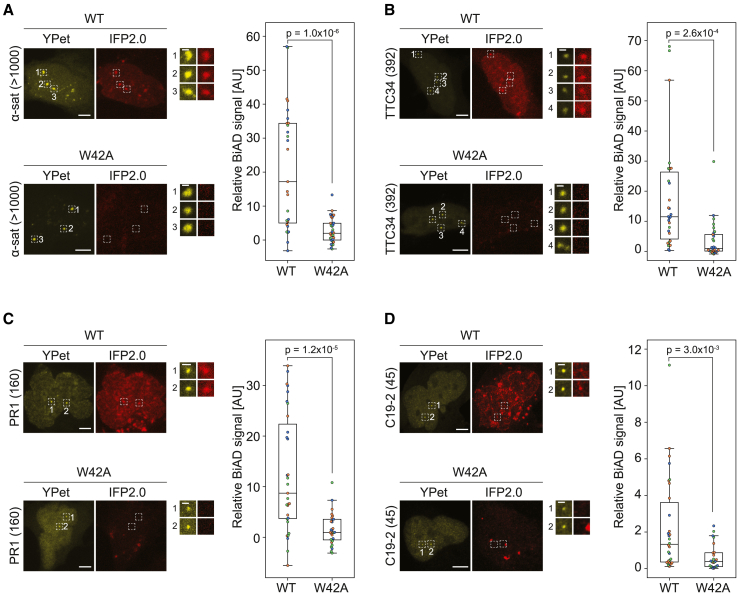
Dual-color BiAD sensor for H3K9me2/3 readout HEK293 cells were transfected with all components of the dual-color BiAD sensor for H3K9me2/3 detection using either the WT 2×HP1βCD detector or a binding-deficient mutant (W42A) as negative control. The BiAD sensor was tested on different target loci with decreasing local repeat numbers: (A) α-satellites (>1,000 repeats), (B) TTC34 (392 repeats), (C) PR1 (160 repeats), and (D) C19-2 (45 repeats). In each panel, exemplary fluorescence microscopy images of fixed cells show the colocalization of the marker (YPet) and BiAD signal (IFP2.0) for the WT detector, but not for the binding-deficient R44Q mutant. Scale bars, 5 μm and 1 μm for the magnified images. Boxplots show the relative BiAD signals. Each dot represents the mean relative BiAD signal of all of the spots within a single cell. Data were obtained in 3 independent experiments (depicted in orange, blue, and green). Significance was determined via a 2-tailed, unpaired t test. The p values are indicated in each boxplot.

After our pilot experiments indicated enhanced H3K9me2/3 binding of HP1βCD as a double domain (2×HP1βCD) in NIH3T3 cells (Figure 3A), we wanted to explore its potential as a detector module in dual-color BiAD sensors to locus-specifically investigate H3K9me2/3 in human cells. Using the double domain detector (2×HP1βCD), we were able to visualize H3K9me2/3 at repetitive endogenous loci in human cells for the first time (Figures 5A–5D). Similar to 5mC, we could detect H3K9me2/3 BiAD signals at various repeat numbers from α-satellites (>1,000 repeats) to 45 repeats at the C19-2 region.

**Figure 6:**
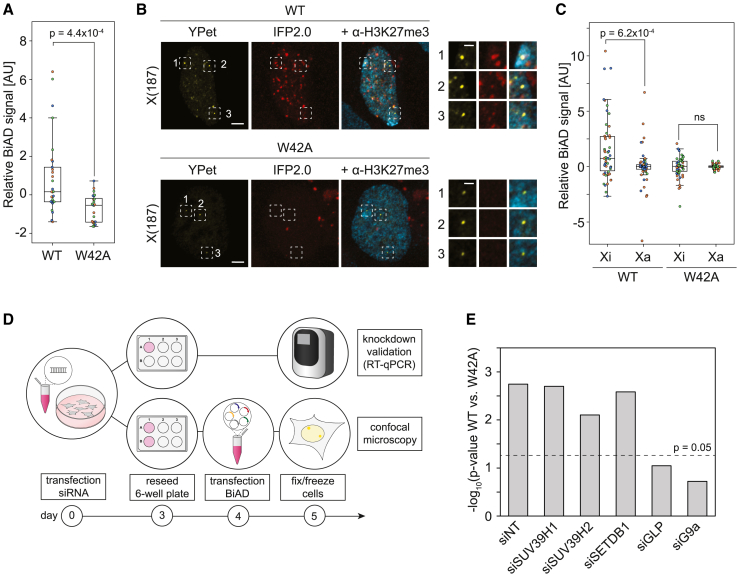
Dual-color BiAD sensor for H3K9me3 readout at the X chromosome (A–C) HEK293 cells were transfected with all components of the dual-color BiAD sensor for H3K9me2/3 detection at the X(187) locus using either the WT 2×HP1βCD detector or a binding-deficient mutant (W42A) as negative control. (A) Boxplots showing the relative BiAD signal in live cells (exemplary images are shown in Figure S4A). Each dot represents the mean relative BiAD signal of all of the spots within a single cell. (B) Fixed cells were immunostained for H3K27me3 (shown in cyan) to identify the inactive (Xi) X chromosomes. Exemplary fluorescence microscopy images showing the colocalization of the marker (YPet) and BiAD signal (IFP2.0) for the WT detector at the Xi (spot 1), less BiAD signal at the Xa (spots 2 and 3), and no colocalization for transfections with the W42A mutant. Scale bars, 5 μm and 1 μm for the magnified images. (C) Individual spots were separated into Xi and Xa and normalized to the mean Xa signal for each cell. The p values are indicated in the boxplots in (B) and (C); ns, nonsignificant p value (p > 0.05). (D and E) HEK293 cells were transfected with siRNAs targeting the human H3K9 methyltransferases SUV39H1, SUV39H2, SETDB1, GLP, and G9a or a control siRNA (siNT) and subsequently reseeded and transfected with the dual-color BiAD sensor for H3K9me2/3 detection at the X(187) locus. (D) Schematic representation of the experimental setup. (E) p values derived from the quantitative analysis (Figure S4C) of fluorescence microscopy images comparing relative BiAD signals of cells transfected with 2×HP1βCD WT and W42A detector modules (exemplary images are shown in Figure S4D).

To further test the H3K9me2/3 double domain detector module, we investigated whether it could be used to distinguish allelic differences in this histone PTM. Therefore, we used the intergenic X(187) region, which was already used for validation of the H3K27me3 detector, to determine whether our H3K9me2/3 BiAD sensor could detect the enrichment of H3K9me2/3 on the Xi that had been reported previously.^39^^,^^40^^,^^41^ Comparing the relative BiAD signals from cells transfected with 2×HP1βCD WT and W42A detector modules, we successfully identified H3K9me2/3 at the X(187) region in live cells (Figures 6A and S4A). To investigate whether our H3K9me2/3 BiAD sensor could detect allelic differences in H3K9me2/3 levels between Xi and Xa, we repeated the experiment in fixed cells and additionally stained the cells for H3K27me3 to identify the Xi (Figure 6B). When separating the individual spots into Xa and Xi, we found that the BiAD sensor detected more H3K9me2/3 at the Xi compared to the Xa (Figures 6B and 6C). Hence, the allelic difference in H3K9me2/3 levels at the X(187) locus on the X chromosomes could be detected by the H3K9me2/3 dual-color BiAD sensor.

### Functional studies of H3K9me3 establishment on Xi using the dual-color BiAD sensor

Finally, we wanted to use our dual-color H3K9me2/3 BiAD sensor to examine the role of different H3K9 protein lysine methyltransferases (PKMTs) in the deposition and maintenance of H3K9me2/3 at the X(187) locus. For this, we used individual small interfering RNA (siRNA) knockdown of the five main human H3K9 PKMTs (SUV39H1, SUV39H2, SETDB1, G9a [EHMT2], and GLP [EHMT1]), followed by transfection of the dual-color BiAD sensor for H3K9me2/3 readout and analysis 5 days after knockdown of the PKMTs (Figure 6D). The efficient individual knockdown of all five PKMTs was confirmed by reverse transcription-qPCR (Figure S4B). Analysis of the BiAD signals comparing the detector WT to the binding-deficient W42A mutant for each knockdown revealed that cells transfected with control siRNA (siNT) as well as after SUV39H1, SUV39H2, and SETDB1 knockdown maintained H3K9me2/3 at the X(187) locus (Figures 6E, S4C, and S4D). In contrast, cells transfected with siRNAs depleting either G9a or GLP showed a markedly reduced BiAD signal at the X(187) locus, with no significant difference in relative BiAD signals obtained from cells transfected with WT or binding pocket mutant detector. These results indicate that both of these enzymes are crucial for H3K9me2 deposition at the X(187) locus, which is in agreement with the finding that they predominantly exist in a heteromeric complex.^42^ In conclusion, our results highlight that dual-color BiAD sensors are valuable tools to gain insights into locus-specific and even allelic chromatin states and the enzymes involved in the regulation of the epigenome network.

## Discussion

Gaining insights into the locus-specific dynamics of epigenome modifications is essential to uncover the mechanisms occurring during cellular differentiation and development of diseases. To study these locus-specific changes at the single-cell level, we developed modular dual-color BiAD sensors that are compatible with live-cell imaging. By combining the fluorescent labeling of an endogenous target locus with the BiFC-based readout of chromatin modifications, we could, for the first time, detect DNA methylation as well as the histone PTMs H3K9me2/3, H3K27me3, and H3K36me2/3 at low copy repeat regions in single human cells. With this improved dual-color BiAD sensor design, the sensor sensitivity was improved strongly, from the detection limit at highly repetitive loci (>1,000 repeats) in the previously available BiAD sensors^17^ to regions with as few as 45 local repeats. Furthermore, we demonstrated that the dual-color BiAD sensors allowed us to observe changes in DNA methylation upon DNMT1i treatment, indicating that it can be used to monitor dynamic responses to drug treatments in cells with locus resolution. Hence, the dual-color BiAD sensors could be applied as a tool for the development and validation of new (epi)drugs.

The dual-color design combining the YPet fluorophore marking the target locus with the split IFP2.0 fluorophore for BiFC readout of the chromatin modification was one critical step to achieve the improved sensitivity of the BiAD sensors. The YPet marker allows the tracking of the target locus in real time during live-cell imaging and facilitates the discrimination of the IFP2.0 BiAD signal at the locus of interest from unspecific nuclear background, enabling the quantitative and qualitative readout of epigenome modifications. The split site in the IFP2.0 fluorophore had been designed for reversible BiFC assays,^25^ which could help to minimize the influence of the BiAD sensors on the three-dimensional (3D) chromatin structure caused by the enforced interaction of anchor and detector binding sites. It had been demonstrated in previous studies that multivalent interactions with the histone PTM increase the affinity of natural reader domains for their respective mark.^29^^,^^31^^,^^32^^,^^33^ Exploiting this principle, we showed that the use of double reader domains enabled the locus-specific detection of the histone PTMs H3K9me2/3, H3K36me2/3, and H3K27me3 in human cells with dual-color BiAD sensors.

It has been shown previously that H3K27me3 and H3K9me2/3 are enriched on the inactive X chromosome.^39^^,^^40^^,^^41^ Using our dual-color BiAD sensors with additional double domain detector modules for H3K27me3 and H3K9me2/3 readout, we were able to detect the allelic enrichment of these marks at the X(187) locus on the inactive X chromosome. Furthermore, the dual-color BiAD sensors were used successfully to dissect the H3K9 PKMT regulatory network at the X chromosome. Using individual knockdowns of the five main human H3K9 PKMTs, G9a, GLP, SUV39H1, SUV39H2, and SETDB1, we identified that G9a and GLP, which are known to form heteromeric complexes,^42^ are mainly responsible for H3K9me2 deposition at the X(187) locus. This result fits to previous data because G9a/GLP have been shown to interact with multiple X chromosome-associated factors such as the long noncoding RNAs Xist and Tsix,^43^ as well as polycomb repressive complex 2, responsible for H3K27me3 deposition at the inactive X chromosome,^44^ and the loss of G9a causes failure of X chromosome inactivation.^43^ These findings highlight the unique potential of dual-color BiAD sensors to investigate the locus-specific changes of epigenome modifications in live cells. A recent study used fluorescently labeled mintbodies to visualize the accumulation of H3K27me3 and H4K20me1 during X chromosome inactivation.^45^ Although this method allowed for tracking the Xi and Xa in live cells using dCas9-EGFP targeted to microsatellite repeats on the X chromosome, it lacked locus-specific resolution of the analysis of the epigenome marks. Using our dual-color BiAD sensor for locus-specific H3K27me3 readout, an additional dimension could be added by analyzing the H3K27me3 dynamics at different dCas9 target loci on the X chromosome to further dissect the spatiotemporal dynamics during the establishment of X chromosome inactivation.

In our experimental setup using the HEK293 cell line, we encountered limitations with the Sirius-MS2 system to visualize regions containing fewer than 45 local repeats. However, even with this current limitation, 254 or 266 endogenous loci in the human reference genomes of female and male cells, respectively, are already within the detection range of our dual-color BiAD sensors (http://genome.ucf.edu/CRISPRbar/).^19^^,^^20^ Among these potential target loci, which, for example, include the TERT gene, 143 (XX)/146 (XY) are either in gene bodies or gene control regions, illustrating potential applications of our dual color BiAD sensors with direct biological significance (Table S1). To explore the full potential of the dual-color BiAD sensors to detect chromatin modifications at even lower copy loci and ultimately with single-locus resolution, it will be essential to enhance the detection limit of the marker fluorophore. This could be achieved by a different sgRNA-based recruitment mechanism, such as the recently published Casilio system for single-locus detection.^22^ Alternative approaches could include tiling of the dual-color BiAD sensors using multiple individual sgRNAs to visualize a nonrepetitive locus.^46^^,^^47^^,^^48^ Moreover, further protein engineering of the reader domain as demonstrated for H3K9me2/3 and H3K27me3 readers^31^^,^^49^ may lead to an increased sensitivity and specificity of the BiAD signal. Achieving single-locus resolution would be particularly valuable in the context of active modifications such as H3K4 methylation or histone acetylation, which mostly occur at nonrepetitive sites. In this context, the double domain approach will be applicable because the improved functionality of corresponding double reader domains has already been demonstrated such as the TAF3 PHD domain for H3K4me3^29^ as well as the second bromodomain of human polybromodomain 1 for H3K14ac readout.^33^

In summary, the dual-color BiAD sensors developed in this study provide a powerful experimental approach for detecting chromatin PTMs at genomic loci containing lower copy repeats and ultimately at single-locus resolution. These sensors offer a modular and versatile toolbox that is compatible with live-cell studies, enabling the visualization of dynamic changes in epigenomic marks at endogenous target loci in single cells and allowing a combined analysis of cellular phenotypes and epigenome modifications. In combination with a broad palette of detector modules for readout of various chromatin PTMs, the dual-color BiAD sensors will greatly enhance our understanding of locus-specific dynamic epigenome regulation processes at the single-cell level. From the long-term perspective, it would be fascinating to express BiAD sensors in animals and follow epigenetic reprogramming of developing tissues with time and cellular resolution in living animals.

### Limitations of the study

As discussed above, the main limitation of the current dual-color BiAD sensors is their restriction to target sites containing ≥45 local repeats. This limitation may be overcome with further optimization, new fluorophores, or improved imaging technology. Moreover, as with any CRISPR-dCas9 methodology, it cannot be excluded that the binding of sgRNA/dCas9 complexes affects chromatin modifications, its 3D structure, and/or binding of endogenous chromatin-binding factors in the timescale relevant to our experiments. On the technical side, one limitation of our study is that in the current design of the sensors, cotransfection of several plasmids is necessary and, therefore, each sample of transfected cells contained cells that did not contain all of the required BiAD parts and hence remained BiAD negative. This problem could be circumvented in future by the generation of stable cell lines expressing some of the BiAD components or by combining multiple BiAD components on the same plasmid. Another technical limitation is that currently the thresholds to define ROIs were manually defined for each nucleus depending on its fluorescence intensity. This setting may be automated in improved versions of the workflow.

## STAR★Methods

### Key resources table

**Table:** 

REAGENT or RESOURCE	SOURCE	IDENTIFIER
**Antibodies**		

α-H3K36me3	Abcam	ab9050
α-H3K27me3	Active Motif	39155
α-H3K27me2/3	Active Motif	39536
α-H3K9me3	Diagenode	C15410193
goat α-rabbit IgG Alexa Fluor™ 405	Invitrogen	A-48258
goat α-mouse IgG Alexa Fluor™ 405	Invitrogen	A-31553
goat α-mouse IgG Alexa Fluor™ 594	Invitrogen	A-11005
goat α-mouse IgG Alexa Fluor™ 633	Invitrogen	A-21052
goat α-rabbit IgG Alexa Fluor™ 647	Invitrogen	A-32733

**Bacterial and virus strains**		

*E. coli* XL1 Blue	Agilent	200228
*E. coli* Stbl3™	Invitrogen	C737303

**Chemicals, peptides, and recombinant proteins**		

DNMT1 inhibitor GSK-3685032	MedChemExpress	HY-139664
Biliverdin	Sigma-Aldrich	30891

**Critical commercial assays**		

Celluspot™ arrays	Active Motif	Cat# 13001

**Deposited data**		

ImageJ macro for image analysis	This paper	https://doi.org/10.6084/m9.figshare.23592894

**Experimental models: Cell lines**		

HEK293	DSMZ	RRID: CVCL_0045
NIH3T3	ATCC	RRID: CRL-1658

**Oligonucleotides**		

Primers for siRNA knockdown validation, see Table S7	This paper	N/A
Primers for 5mC-sensitive qPCR, see Table S7	This paper	N/A
siRNAs used for H3K9 methyltransferase knockdown, see Table S5	This paper	N/A

**Recombinant DNA**		

Plasmids deposited with Addgene, see Table S7	This paper	N/A

**Software and algorithms**		

FIJI, ImageJ 1.54b	Schindelin et al.^50^	https://imagej.net/software/fiji/
Graphpad Prism 5	Graphpad Software, Inc	N/A
Illustrator CS6	Adobe	N/A
Python 3.9.7	Python Software Foundation	https://www.python.org/downloads/
Matplotlib	Hunter et al.^51^	N/A
Seaborn	Waskom et al.^52^	N/A
Excel 2016	Microsoft	N/A
ZEN 3.0 SR (black edition) and Zen 3.0 (blue edition)	Zeiss	N/A

**Other**		

sgRNA sequences, see Table S2	Ma et al.^20^	http://genome.ucf.edu/CRISPRbar/

### Resource availability

#### Lead contact

Further information and requests for resources should be directed to and will be fulfilled by the lead contact, Albert Jeltsch (albert.jeltsch@ibtb.uni-stuttgart.de).

#### Materials availability

Plasmids generated in this study have been deposited to Addgene (Table S2).

#### Data and code availability

• All data reported in this paper will be shared by the lead contact upon request.
• All original code used for image analysis has been deposited at figshare.com and is publicly available as of the date of publication. DOIs are listed in the key resources table.
• Any additional information needed to reanalyze the data reported in this paper is available from the lead contact upon request.

### Experimental model and study participant details

#### Cell culture

Human embryonic kidney (HEK293; female; RRID: CVCL_0045) and NIH3T3 (male; RRID: CRL-1658) cells were obtained from DSMZ (Braunschweig, Germany) and ATCC (American Type Culture Collection), respectively. HEK293 and NIH3T3 cells were cultivated in DMEM culture medium (DMEM supplemented with 10% Fetal Bovine Serum (FBS), 1% penicillin/streptomycin, 4 mM L-glutamine) in a humidified incubator (BINDER) at 37°C and 5% CO_2_. To maintain a confluency of 70–90%, cells were split at a 1:7 to 1:10 ratio every 2 to 3 days. For this, the cells were washed with PBS (without CaCl_2_ and MgCl_2_), followed by addition of trypsin-EDTA solution (Sigma) and incubation at 37°C until cells detached. Afterward, cells were resuspended using culture medium and split in the desired ratio. For storage, cells were harvested at 300 g for 5 min, resuspended in freezing medium (90% FBS, 10% DMSO) and gradually frozen to −80°C. Cells were routinely checked for mycoplasma contamination by PCR.^53^

### Method details

#### Retrieval of repetitive genomic loci and cloning of sgRNA and dCas9-SunTag vectors

Endogenous loci in the human genome containing repetitive sequences, which can be targeted by CRISPR-imaging, were retrieved from the CRISPRbar web server^54^ (http://genome.ucf.edu/CRISPRbar/) using default settings. A subset of them containing gene-associated target loci within the detection range of the dual-color BiAD sensors is provided in Table S1. For cloning, the 12 nucleotides preceding the PAM sequence (NGG) were taken as sgRNA sequence. A list of all sgRNA sequences used in this study is provided in Table S3. The individual sgRNA sequences taken from^20^^,^^54^ were inserted into pPUR-hU6-sgRNA-Sirius-8XMS2 using Golden Gate Assembly as described in.^54^ pPUR-hU6-sgRNA-Sirius-8XMS2 was a gift from Thoru Pederson^20^ (Addgene plasmid #121942; RRID:Addgene_121942).

To generate an expression construct for the GCN4-binding single-chain fragment variable (scFv) fused to the N-terminal part of mVenus or IFP2.0, the region encoding sfGFP in pHR-scFv-GCN4-sfGFP-GB1-NLS-dWPRE was replaced by the N-terminal part of split mVenus (amino acid 1–210, Addgene plasmid # 27794) or IFP2.0 (amino acid 1–132, Addgene plasmid # 54784) using the Gibson Assembly mix (New England Biolabs). pHR-scFv-GCN4-sfGFP-GB1-NLS-dWPRE was a gift from Ron Vale^18^ (Addgene plasmid #60906; RRID:Addgene_60906). IFP2.0-C1 was a gift from Michael Davidson & Xiaokun Shu^55^ (Addgene plasmid # 54784; RRID:Addgene_54784).

To obtain an expression construct for the MS2 coat protein (MCP) RNA-binding domain fused to YPet, the HaloTag in pHAGE-EFS-MCP-HALOnls was replaced with YPet from pcDNA3-Cyto-CaNAR2 using Gibson Assembly. The pHAGE-EFS-MCP-HALOnls plasmid was a gift from Thoru Pederson^20^ (Addgene plasmid # 121937; RRID:Addgene_121937). The pcDNA3-Cyto-CaNAR2 plasmid containing the YPet fluorophore was a gift from Jin Zhang^56^ (Addgene plasmid # 64729; RRID:Addgene_64729).

The dCas9-SunTag plasmid pCAG-dCas9-5xPlat2AflD was a gift from Izuho Hatada^57^ (Addgene plasmid # 82560; RRID:Addgene_82560). This plasmid was used for DNA methylation detection. For detection of histone posttranslational modifications, five additional copies of the GCN4 epitope were inserted using the BamHI restriction site to generate a SunTag with ten GCN4 epitopes, separated by 22 amino acid linkers.

#### Cloning of detector domains

All chromatin reader domains were cloned as single or double domain into the mVenus-C1 mammalian expression vector from Steven Vogel^58^ (Addgene plasmid # 27794; RRID:Addgene_27794) using the Gibson Assembly mix as described in.^17^ For the double domain plasmids, the domains were separated by a 12 amino acid linker containing an SV40 large T-antigen monopartite NLS. For testing the specificity of detector binding or application in BiAD sensors, the mVenus fluorophore was replaced by dsRed or the C-terminal part of IFP2.0 (amino acid 133–321), respectively, by Gibson assembly.

The MBD1 MBD domain and its R44Q binding pocket mutant for DNA methylation readout were taken from.^17^ For use in the dual-color BiAD sensor, the C-terminal part of mVenus was exchanged to the C-terminal part of IFP2.0. The CBX7 chromodomain (aa 1–76 of human CBX7, accession number NP_783640.1) for H3K27me3 readout was cloned as single or double domain as described above. The W35A point mutation was introduced by site-directed mutagenesis PCR.^59^ For H3K36me2/3 detection, the PWWP domain of human DNMT3A1 (amino acid 283–425, accession number NP_783328.1)^34^ was cloned as single or double domain into the mVenus-C1 expression vector as described above. The K299E, R366E and D333A point mutations were introduced by site-directed mutagenesis PCR.^59^ For H3K9me2/3 readout, the previously used mouse HP1β chromodomain (amino acid 17–76, accession number NP_031648)^17^ and the W42A binding pocket mutant were taken and cloned as single or double domain as described above.

#### Transfections

For transient transfections, HEK293 or NIH3T3 cells were seeded either on glass cover slips in a 6-well plate or, for live-cell imaging, in a 35 mm glass bottom Fluorodish (World Precision Instruments) at a density of 200,000 cells per well one day before transfection. Right before transfection, the medium was exchanged with 1.5 mL fresh culture medium. For transfections with the fluorescent protein IFP2.0, the medium was supplemented with 30 nM biliverdin which serves as the chromophore. The plasmid amounts for the various transient transfection reactions are given in Table S4. Each well was transfected with 1.5 μg total DNA using FuGENE (Promega) according to the manufacturer’s instructions.

#### Fixation and antibody staining

Cells were fixed 24 h after transfection unless otherwise indicated. Before fixation, the cells were washed three times for 5 min with PBS with CaCl_2_ and MgCl_2_ (PBS+) to prevent detachment of the cells during the washing steps. Fixation was conducted for 10 min with 4% paraformaldehyde. Afterward, cells were washed three times for 5 min each with PBS+ and either mounted directly onto glass slides with a drop of fluoromount G with or without DAPI (Invitrogen) or antibody staining was performed.

For antibody staining, cells were permeabilized and blocked for 30 min at room temperature using saponin blocking buffer containing 5% (v/v) fetal bovine serum, and 0.1% (w/v) saponin (Roth) in PBS+. The cells were incubated with antibodies against the respective histone mark overnight at 4°C, whereby the antibodies were diluted in saponin antibody dilution buffer containing 1% (w/v) Albumin, Bovine (Sigma-Aldrich), and 0.1% (w/v) saponin (Roth) in PBS+. On the next day, the slides were washed three times for 5 min and then incubated with the corresponding secondary antibody diluted in saponin antibody dilution buffer for 1 h at room temperature in the dark. After three additional 5 min washing cycles with PBS+, the glass slides were mounted with a drop of fluoromount G with or without DAPI (Invitrogen). A list of primary and secondary antibodies for immunostainings is provided in Table S5.

The specificity of all primary antibodies used in this study was validated using CelluSpot arrays (Active Motif), basically as described in.^60^ The α-H3K9me3 antibody showed a clear preference for H3K9me3 with only weak binding to H3K9me2 (Figure S5A). The α-H3K36me3 antibody demonstrated binding to H3K36me3, however, we observed some cross-reactivity with H4K20me3, H3K27me3 and H3K9me3 on peptide arrays (Figure S5B). During this study, two different antibodies were used for H3K27me3 immunostaining. The polyclonal α-H3K27me3 antibody (Active motif 39155) displayed high specificity toward H3K27me3 (Figure S5C). Due to the batch-to-batch variability of polyclonal antibodies, we also evaluated a monoclonal α-H3K27me2/3 antibody as an alternative (Figure S5D). Since we observed strong binding to H3K27me3 with only a weak signal for H3K27me2 in our specificity analysis, we utilized both antibodies for H3K27me3 immunostaining.

#### Fluorescence microscopy and image analysis

The samples were imaged using a confocal laser scanning microscope (LSM710 or LSM980 Airyscan 2, Carl Zeiss) equipped with a Plan-Apochromat 63x/1.4 Oil DIC M27 objective. The laser excitation wavelength and emission collection windows were ex. 405 nm/em. 410–479 nm for DAPI/Alexa 405, ex. 514 nm/em. 515–544 nm or em. 515–621 nm, if the 561 nm laser was not used in the experiment, for mVenus/YPet, ex. 561 nm/563–621 nm for dsRed, ex. 633 nm (LSM710)/ex. 639 nm (LSM980)/em. 638–747 nm for IFP2.0, Alexa 633 and Alexa 647 dyes. The image acquisition settings were kept constant within the same experiment. Z-stacks through the nucleus were collected with an interval of 0.5 μm (LSM710) or 0.13 μm (LSM980). Live-cell imaging was conducted at 37°C and 5% CO_2_. The stacks were superimposed to gain the maximum intensity projection using the lite ZEN 3.0 SR (black edition) software. For image export, the Zen 3.0 (blue edition) software was used. The exemplary images were exported with the same brightness and contrast settings for each comparison of WT and mutant sensors.

Images were analyzed using a custom FIJI (ImageJ 1.54b)^50^ macro without adjustments of contrast and brightness. Based on the YPet channel, two intensity thresholds were set manually for each cell to define the nucleus and spots as regions of interest (ROIs) (see Figure S1C). First, a low intensity threshold was applied to define the nuclear background signal. Subsequently, a second, high-intensity threshold was defined to identify the brightest YPet spots within the nucleus with a maximum of six spots per cell. Afterward, the mean intensity of the ROIs was measured in all channels. For quantitative analysis, the nuclear signal intensity was subtracted from the spot intensities for each channel. Then, the BiAD spot intensity (IFP2.0 channel) was normalized to the corresponding YPet spot intensity (Figure S1C; equation 1). Unless otherwise stated in the figure legends, the normalized BiAD signals of all spots within one cell were averaged and are represented as dot in the boxplots (Figure S1C; equation 2). Box-, swarm- and stripplots were generated using the Seaborn Python data visualization library.^52^^,^^51^

#### siRNA knockdown of H3K9 methyltransferases

For individual transient knockdown of the five mammalian H3K9 methyltransferases EHMT1 (GLP), EHMT2 (G9a), SETDB1, SUV39H1 and SUV39H2, 0.5x10^6^ cells were seeded in a 6 cm dish and siRNAs were transfected with RNAiMAX (Invitrogen) according to the manufacturer’s instructions. The siRNAs used were purchased from ThermoFisher. A list of all siRNAs is provided in Table S6. Three days post transfection, cells were reseeded either onto glass slides for microscopy or in a 6-well cell culture dish for knockdown validation. Transfections of the dual-color BiAD sensor were carried out as described above. Cells were either fixed for microscopy or snap frozen for knockdown validation five days after siRNA transfection.

For knockdown validation, total RNA was isolated from cells using the RNeasy Plus Mini Kit (Qiagen) according to the manufacturer’s instructions. Subsequently, mRNA was transcribed to cDNA and gene expression was determined by qPCR basically as described in.^61^ The gene expression was calculated using the ΔΔCt method relative to the siRNA negative control and SDHA as housekeeping gene. All primer sequences used for knockdown validation are provided in Table S7.

#### DNMT1 inhibitor treatment

HEK293 cells were seeded onto glass slides and transfected as described in the previous section. The medium exchange before transfection contained 30 nM biliverdin (Sigma-Aldrich) and 10 μM DNMT1 inhibitor (GSK-3685032, MedChemExpress, dissolved in DMSO)^28^ or an equal volume of DMSO as control. Cells were fixed three days after transfection. For analysis of global DNA demethylation, the genomic DNA (gDNA) was isolated using the QIAamp DNA Mini Kit (Qiagen) according to the manufacturer’s instructions. Afterward, 500 ng gDNA were digested at CCGG sites with MspI (5mCpG insensitive) or HpaII (5mCpG sensitive) for 1 h at 37°C. Afterward, the digested products as well as undigested control samples were analyzed on an agarose gel (1%) stained with GelRed and imaged using a Quantum imaging system (Vilber).

For locus-specific 5mC analysis, two amplicons in the PR1 locus were designed that contained HpaII restriction sites (CCGG). If the CpG site within the HpaII recognition sequence is methylated, HpaII does not cleave the DNA and the qPCR template remains intact. DNA methylation at these sites was analyzed by qPCR conducted basically as described in^61^ using undigested and HpaII digested genomic DNA (5–20 ng) from three biological replicates isolated from DNMT1i and DMSO treated cells. Afterward, the template quantities were calculated using a standard curve. Variations in DNA amounts between samples were normalized using a control amplicon in the PR1 locus, which does not contain HpaII restriction sites. Primer sequences used for qPCR are provided in Table S7. Subsequently, HpaII digested samples from DNMT1i and DMSO treated cells were normalized to the respective undigested gDNA to identify the relative methylation levels.

### Quantification and statistical analysis

Microscopy images were quantified using a custom FIJI (ImageJ 1.54b) macro (https://doi.org/10.6084/m9.figshare.23592894) and Microsoft Excel 2016 as described in “fluorescence microscopy and image analysis”. Statistical analyses were conducted using Microsoft Excel 2016 and GraphPad Prism 5. All details regarding statistical analysis are provided the respective figure legends and figures, including numbers of replicates for each experiment, statistical tests used and the obtained p values. The boxplots depict the median of the data as horizontal line with the box drawn from the first quartile to the third quartile and whiskers extending to 1.5 times the interquartile range.

## Supplemental information

Document S1. Figures S1–S5 and Tables S1–S7

Document S2. Article plus supplemental information
