# Supplementary material for: Modular dual-color BiAD sensors for locus-specific readout of epigenome modifications in single cells
Source: Cell Rep Methods. 2024 Mar 29;4(4):100739. doi: 10.1016/j.crmeth.2024.100739 (PMC11045877; doi:10.1016/j.crmeth.2024.100739)
Supplement: Document S1. Figures S1–S5 and Tables S1–S7 [file mmc1.pdf]

Cell Reports Methods, Volume *4*

Supplemental information

Modular dual-color BiAD sensors for locus-specific readout of epigenome modifications in single cells

Anja R. Köhler, Johannes Haußer, Annika Harsch, Steffen Bernhardt, Lilia Häußermann, Lisa-Marie Brenner, Cristiana Lungu, Monilola A. Olayioye, Pavel Bashtrykov, and Albert Jeltsch

Supplemental Figures

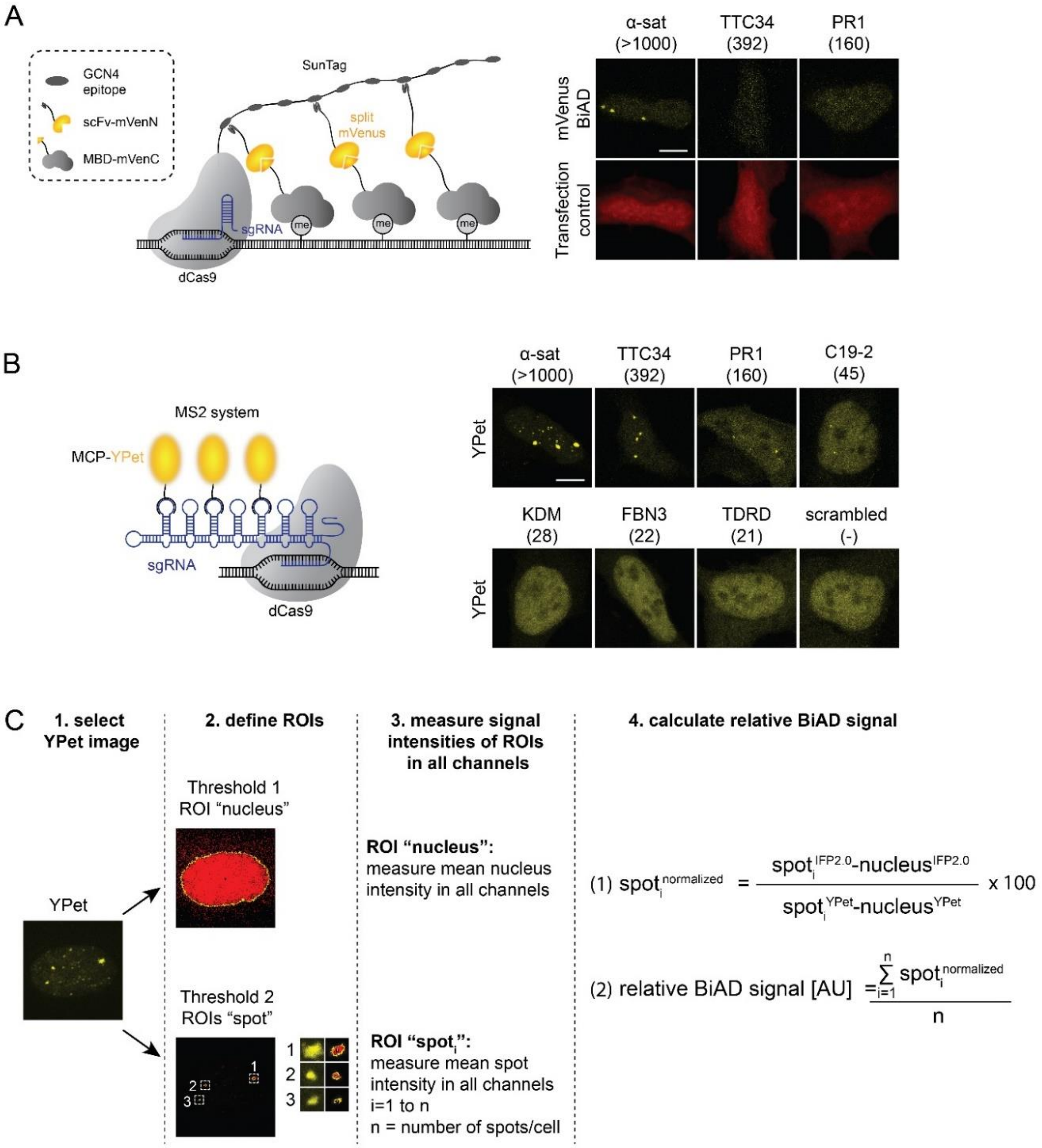

Figure S1. Development of BiAD sensors with enhanced sensitivity at repeats with lower local copy number, related to STAR Methods. A) Amplification of the BiAD signal by recruiting multiple detector modules via a 10xSunTag fused to dCas9. Exemplary fluorescence microscopy images of fixed HEK293 cells transfected with the BiAD sensor plasmids. **B)** Evaluation of the MS2-based CRISPR-Sirius imaging system in fixed HEK293 cells at different target loci. **C)** Quantitative analysis of the dual-color BiAD microscopy images using ImageJ. Two user defined thresholds are set, the first to define the nucleus and the second to define target spots in the YPet channel. The average nuclear background is subtracted from mean spot intensity for YPet and IFP2.0 channels. Then, the obtained IFP2.0 BiAD signal is normalized to the YPet marker signal. Finally, the relative BiAD signal for each cell is calculated as the mean of all normalized spots within this cell.

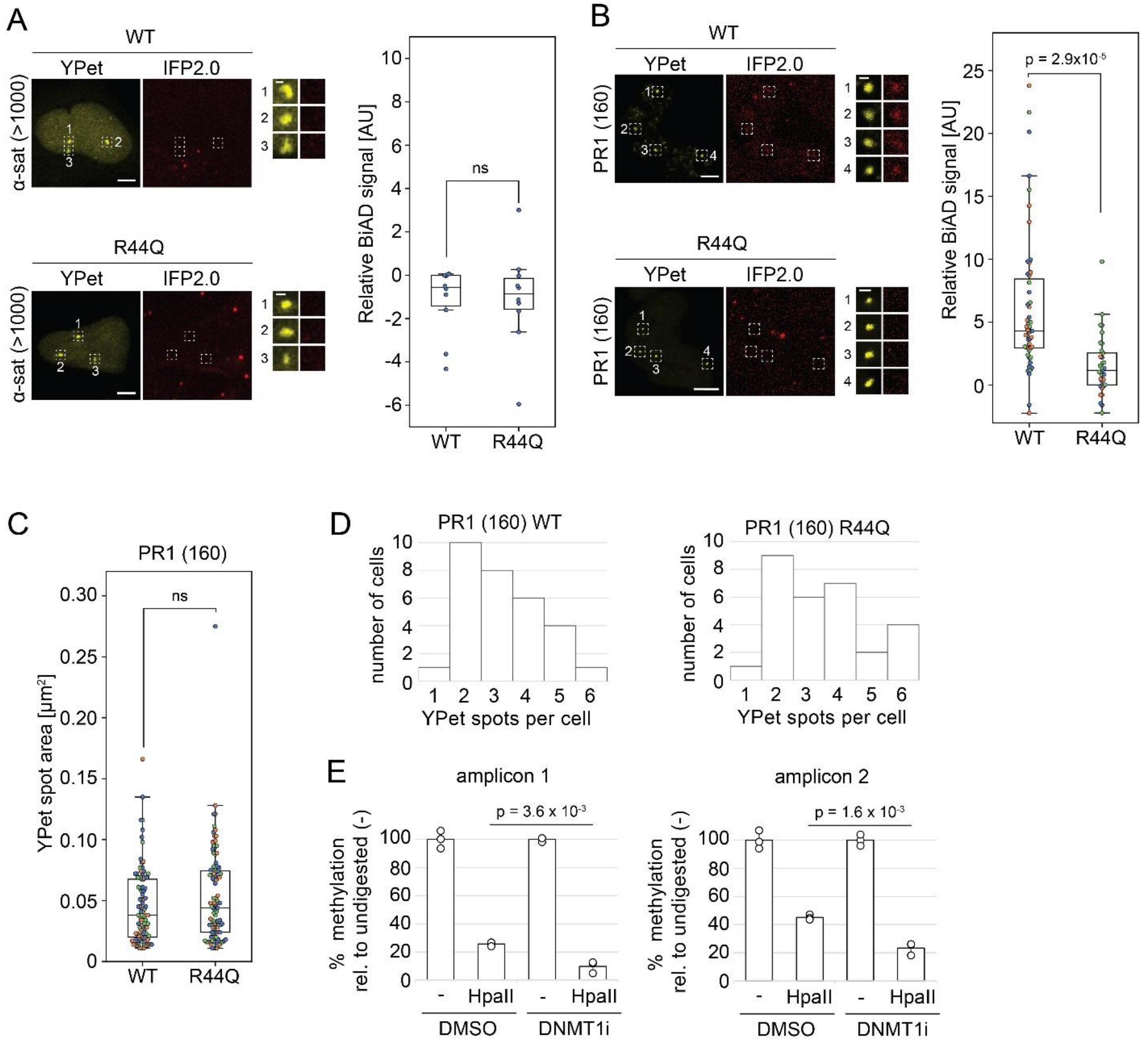

Figure S2. Additional validation of BiAD sensors for DNA methylation readout, related to Figure 1 and Figure 2. A) HEK293 cells were transfected with only four components of the dual-color BiAD sensors for 5mC detection at α-satellites (>1000 repeats), lacking the scFv-IFP2.0N expressing plasmid. Exemplary fluorescence microscopy images of fixed cells showing very low IFP2.0 (BiAD) signals and no co-localization between YPet (marker) and residual BiAD signal for wildtype and binding-deficient R44Q detector. **B)** Exemplary live-cell fluorescence microscopy images of HEK293 cells transfected with all components of the dual-color BiAD sensor for 5mC detection at the PR1 locus (160 repeats) showing the co-localization of the marker (YPet) and BiAD signal (IFP2.0) for the wildtype detector, but not for the binding-deficient R44Q mutant. **A-B)** Box plots show the relative BiAD signals. Each dot represents the mean relative BiAD signal of all spots within a single cell. Scale bar is 5 µm and 1 µm for the magnified images. **C-D)** Image analysis related to Figure 1D showing **C)** YPet spot area and **D)** spot count per nucleus for HEK293 cells transfected with all components of the dual-color BiAD sensor for 5mC detection with either the wildtype MBD1 detector (WT) or a 5mC binding-deficient mutant (R44Q). **E)** Exemplary validation of genomic DNA demethylation at the PR1 locus by DNMT1 inhibitor treatment. Genomic DNA was isolated from DNMT1i/DMSO-treated cells and digested at CCGG sites with HpaII (5mCpG sensitive). qPCR on digested and undigested (-) genomic DNA showing reduced 5mC at two amplicons within the PR1 locus for cells treated with DNMT1i compared to mock-treated cells (DMSO). Bar diagrams display the mean of three independent experiments (indicated as dots) ± SD. Significances were determined by a two-tailed, unpaired t-test. The p-values are indicated in the boxplots and bar diagrams, "ns" indicates a non-significant p-value ($p>0.05$).

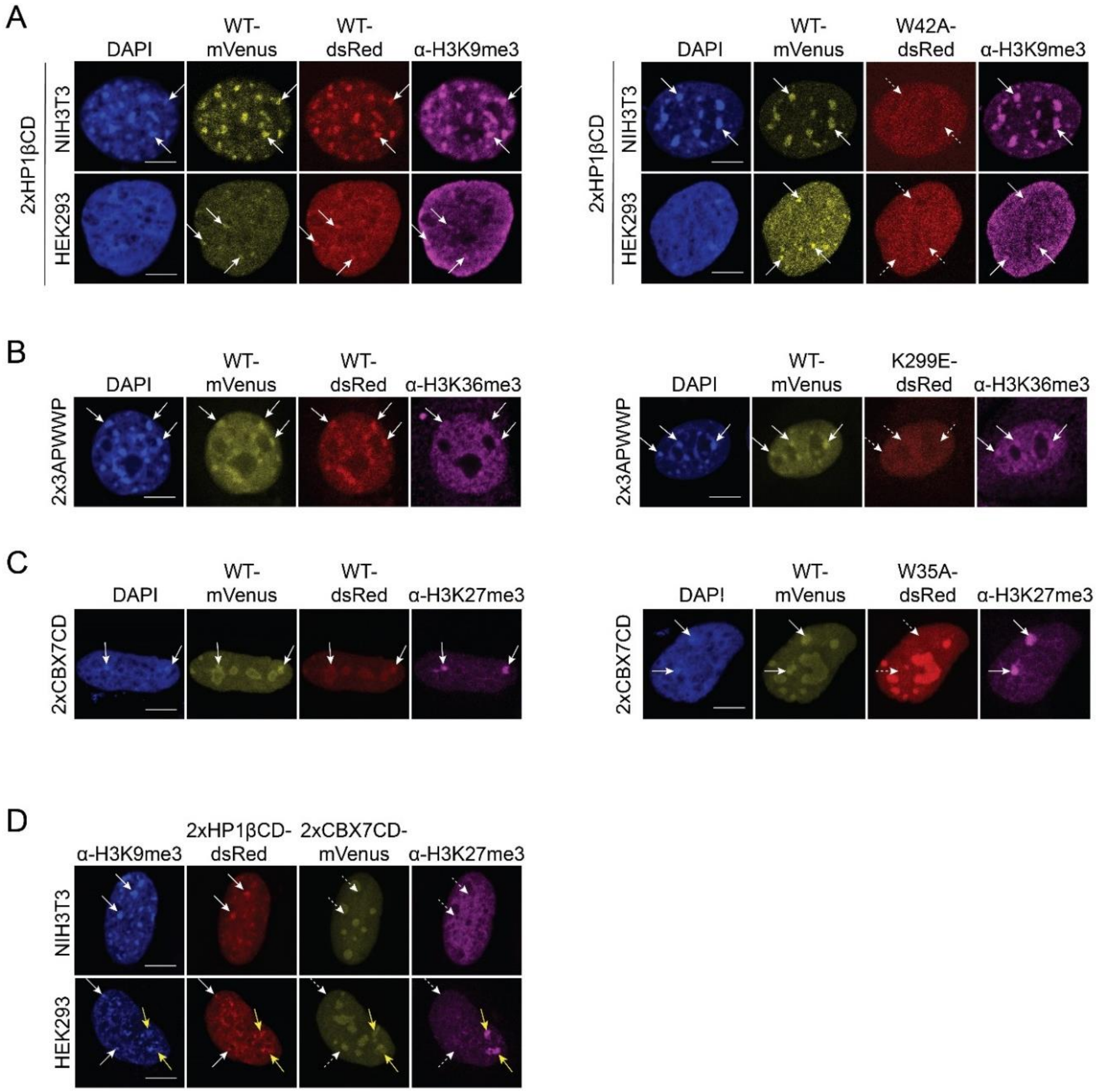

Figure S3. Validation of double reader domains as BiAD detector modules, related to Figure 3. A) Co-transfection of mVenus/dsRed-fused 2xHP1βCD WT and W42A detector modules in NIH3T3 and HEK293 cells combined with α-H3K9me3 and DAPI staining. **B)** Cotransfection of mVenus/dsRed-fused 2x3APWWP WT and K299E detector modules in NIH3T3 cells combined with α-H3K36me3 and DAPI staining. **C)** Cotransfection of mVenus/dsRed-fused 2xCBX7CD WT and W35A detector modules in HEK293 cells combined with α-H3K27me3 and DAPI staining. The co-localization of the detector with the antibody staining and DAPI are exemplarily indicated by arrows in panels A-C. Dotted arrows indicate missing co-localization of the respective binding pocket mutant. **D)** Cotransfection of 2xHP1βCD-dsRed and 2xCBX7CD-mVenus detector modules in NIH3T3 and HEK293 cells combined with α-H3K9me3 and α-H3K27me3 staining. Co-localization of 2xHP1βCD-dsRed and H3K9me3 are exemplarily indicated with arrows. Dotted arrows mark missing co-localization of 2xCBX7CD-mVenus with H3K9me3. Yellow arrows indicate the Xi in HEK293 with co-localization in all channels. All panels show images of fixed cells.

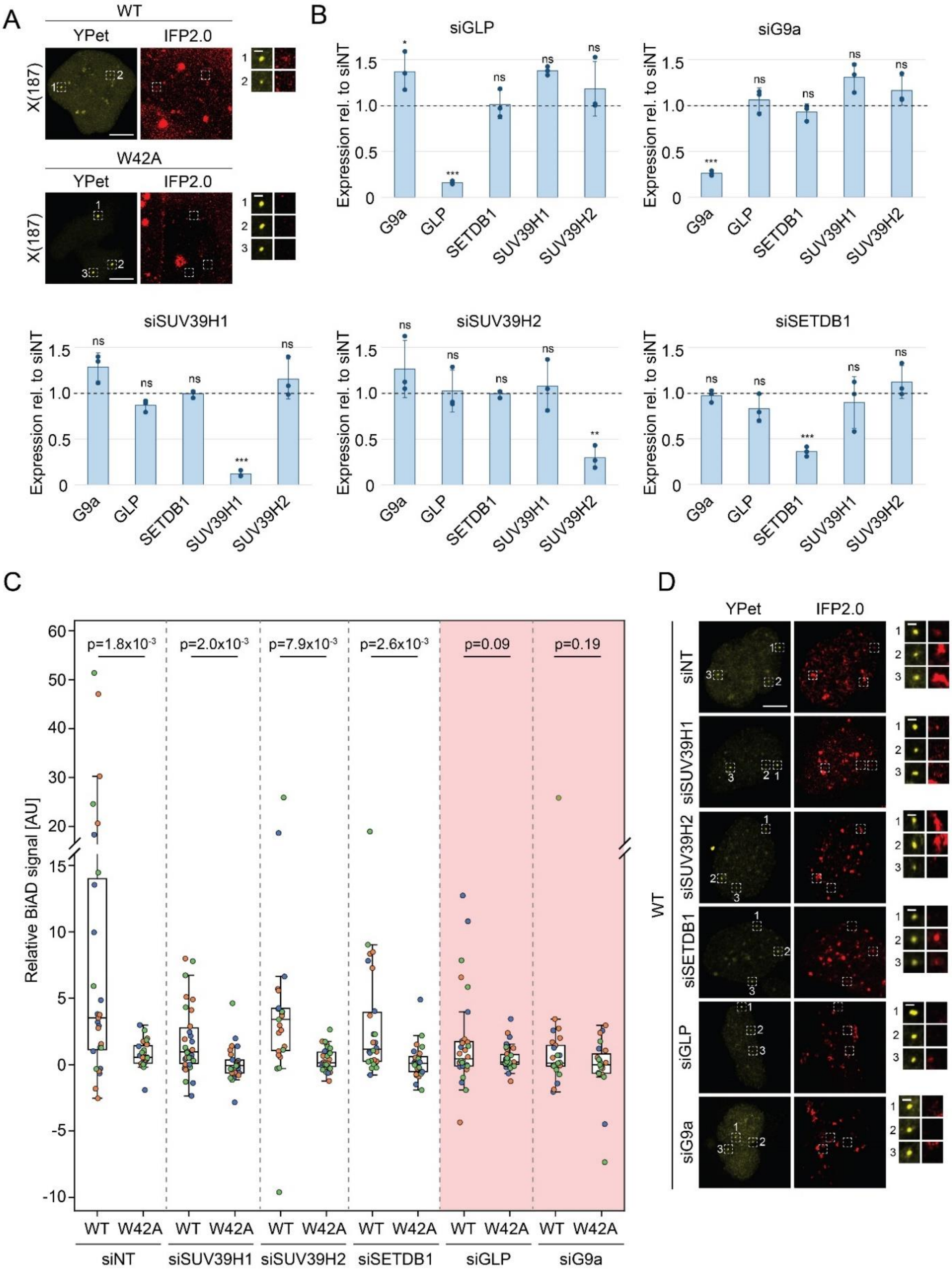

A
WT
YPet
IFP2.0
X(187)
W42A
B
siGLP
siG9a
Expression rel. to siNT
G9a
GLP
SETDB1
SUV39H1
SUV39H2
siSUV39H1
siSUV39H2
siSETDB1
C
Relative BiAD signal [AU]
p=1.8x10-3
p=2.0x10-3
p=7.9x10-3
p=2.6x10-3
p=0.09
p=0.19
WT W42A
siNT
siSUV39H1
siSUV39H2
siSETDB1
siGLP
siG9a
D
YPet
IFP2.0
WT
siNT
siSUV39H1
siSUV39H2
siSETDB1
siGLP
siG9a

Figure S4. H3K9 PKMT siRNA knockdown validation and investigation of H3K9me2/3 changes at the X(187) locus, related to Figure 6. A) Exemplary live cell fluorescence microscopy images related to Figure 6A. **B)** RT-qPCR knockdown validation using siRNAs targeting the human H3K9 PKMTs SUV39H1, SUV39H2, SETDB1, GLP and G9a relative to a control siRNA (siNT). Bar diagrams illustrate the PKMT expression relative to control siRNA (siNT) five days after siRNA transfection showing the mean of three independent experiments (indicated as dots) ± SD. Significance was determined by one-way ANOVA and Dunett post-test using siNT as the control group; * $p \leq 0.05$, ** $p \leq 0.01$, *** $p \leq 0.001$, “ns” $p > 0.05$. **C)** Four days after siRNA transfection, HEK293 cells were transfected with all components of the dual-color BiAD sensor for H3K9me2/3 detection at the X(187) locus using either the wildtype 2xHP1βCD detector (WT) or a binding-deficient mutant (W42A) as negative control. Boxplots show the relative BiAD signal at the X(187) locus in cells upon knockdown of different H3K9 PKMTs. Each dot represents the mean relative BiAD signal of one cell. Data were obtained in three independent experiments (depicted in orange, blue and green). Significance was determined via a two-tailed, unpaired t-test. The p-values are indicated in each boxplot and shown in Figure 6E. **D)** Exemplary fluorescence microscopy images of fixed cells transfected with WT detector from panel B). Scale bar is 5 µm and 1 µm for the magnified images.

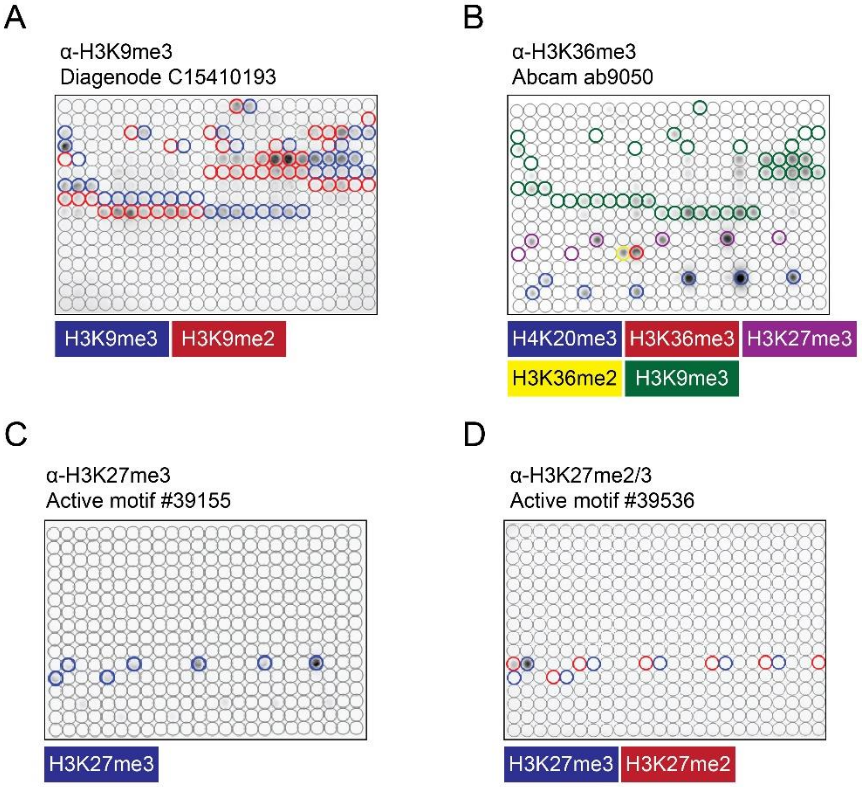

Figure S5. Antibody validation on Celluspots™ peptide arrays, related to STAR Methods. A) α-H3K9me3 (Diagenode C15410193). **B)** α-H3K36me3 (Abcam ab9050). **C)** α-H3K27me3 (Active motif #39155). **D)** α-H3K27me2/3 (Active motif #39536). Relevant peptide spots are annotated below each array.

Supplemental Tables

Table S1. Selection of target loci in the human genome that could be analyzed with the BiAD sensors described here, related to STAR Methods. The loci contain 45 or more local repeats and they are associated with a gene as indicated.

Repetitive sequence (5'-3')	Repeats	Genomic region (hg19)	Gene
CCCGTGTCTCTGCTC	59	Chr1:1223580-1225633	SCNN1D
TTAGGGTCACGGTGG	83	Chr1:2052916-2055861	PRKCZ
TCACACTCTCCCTGG	45	Chr1:227143014-227144982	COQ8A
CCAGGGAAAGCCATC	79	Chr1:246137768-246140373	SMYD3
CCACGCTCCCTAGAT	70	Chr1:246683178-246685393	LINCO1743
CCACAGGTGAGCATC	392	Chr1:2581275-2634211	TTC34
CAGAGAGAAGGGTGG	57	Chr2:1008176-1010495	SNTG2
CACTCGGGTGCAGGG	56	Chr2:1101637-1104810	SNTG2
CCTTACAGTCCTTTG	46	Chr2:1107429-1111487	SNTG2
CCCTCCCGCCTCTCT	53	Chr2:112896304-112897931	FBLN7
CCCTGTCCTGCCTGC	117	Chr2:1218818-1227201	SNTG2
CCCGAGAACTGTGCT	65	Chr2:135364479-135367491	TMEM163
CCCCTGTGTGCAACC	103	Chr2:1526480-1541951	TPO
AACTCCAGGTGAAGG	55	Chr2:1706738-1708450	PXDN
GACAGTGGTGAGTGG	83	Chr2:238451302-238453899	MLPH
CCTGGTGCAGTGCTC	59	Chr2:239441454-239459820	LINCO1107
CCTCCTCCACCTCGG	56	Chr2:241847109-241848811	CROCC2
CCCCCACACTAACCC	55	Chr2:241862860-241864454	CROCC2
GGTAGGAGCCTCTGG	112	Chr2:241915551-241920737	CROCC2
GATGGGAGCCCCGGG	45	Chr2:242704672-242706929	D2HGDH
CCGCGTGATTGCCAG	66	Chr2:242948556-242952808	LINCO1237
CCCTCCATCCAGCCC	46	Chr2:3298022-3300541	EIPR1
CCGCCTCCTCCACCA	70	Chr3:126580436-126583815	CHCHD6
CCCCTCTACTCACCC	46	Chr3:133132825-133137011	BFSP2
CCACACCCATACCTT	120	Chr3:195502236-195504816	MUC4
GGTGACAGGAAGCGG	100	Chr3:195505721-195515533	MUC4
CCTTAACTAGTCTGA	67	Chr3:197739385-197742639	LMLN
AAGTAAAATTGATGG	99	Chr3:77338424-77345592	ROBO2
AGGATGAGGGAAAGG	64	Chr4:184771961-184773735	STOX2
CCATCATACTTCCTA	88	Chr4:187353431-187357853	F11-AS1
CCCGATGTCCAAATA	48	Chr4:189424308-189430772	LINC01060
CCCCTACCCCATGCT	54	Chr4:662196-663594	PDE6B
GAGCAGGTGGGCGGG	54	Chr4:738471-741507	PCGF3
TGTGTTGGGGTCAGG	103	Chr4:7454523-7457556	SORCS2
CCCTGCTCTTCCCAC	107	Chr5:1022265-1026738	NKD2
CCCCAGTGAGCCCCC	93	Chr5:1070083-1073429	SLC12A7
GACAGGGACACCCGG	53	Chr5:1289185-1291897	TERT
CCAGCTCCTCCTCTA	74	Chr5:1326410-1329137	CLPTM1L
CCACAGCTTCCCATC	57	Chr5:176297770-176300222	UC5A
AGTCAGCGACTGAGG	48	Chr5:356563-359536	AHRR
CCAGTCCTGGTGGGG	98	Chr5:629494-633663	CEP72
GGTGTGACTGTGAGG	92	Chr5:648309-651530	CEP72
CCCCATCATAGTGTC	45	Chr6:157731388-157735394	TMEM242
GCATCTTTCTAGAGG	55	Chr6:168992931-168995966	SMOC2
ACAGCCAGCACAGGG	63	Chr6:169014272-169016452	SMOC2
CCTGCCGCACCTAGT	92	Chr6:3090978-3094477	RIPK1
GAGTGCTGGTTCCGG	50	Chr6:3145256-3147143	BPHL
AGTGGAGTCCTGTGG	71	Chr7:1040958-1043677	C7orf50
GGTGAATCACCATGG	78	Chr7:154451869-154455994	DPP6
CACTGAACTCGGTGG	83	Chr7:157439520-157442716	PTPRN2
CCATCTACCCATGCA	47	Chr7:157488518-157490732	PTPRN2
CCAGCACAGTTACCC	58	Chr7:157524153-157527056	PTPRN2
CACACTCCTGCAGGG	56	Chr7:157650733-157652746	PTPRN2
CCACACTCCATCTGC	54	Chr7:157850813-157853026	PTPRN2
CCCCATCTCACGCCA	49	Chr7:157939607-157941888	PTPRN2
CCACACTCTCACCAT	151	Chr7:158122661-158135328	PTPRN2
CCTGCGCACCGCCGG	53	Chr7:158217836-158220732	PTPRN2
CCCTGGAGTGGAGTC	46	Chr7:158316035-158317251	PTPRN2
GAGAAAGGAGAAAGG	81	Chr7:944997-946293	ADAP1
GAGGAGGAGGAAGGG	46	Chr7:981913-983521	ADAP1

Repetitive sequence (5'-3')	Repeats	Genomic region (hg19)	Gene
CCATGTCTAGTTCTC	97	Chr8:1184911-1191424	DLGAP2
CCGTGCGGGTCCTGA	46	Chr8:1298340-1300510	DLGAP2
CCAGAGTCGTGTATT	272	Chr8:1341179-1356081	DLGAP2
CAGGAGGAGCCAGGG	46	Chr8:140767496-140769803	TRAPPC9
AGATGTGTGCTCAGG	66	Chr8:145603817-145607540	ADCK5
CCAGTGGTTTCCATC	69	Chr8:145786505-145788291	ARHGAP39
CCTCACCTGTGTCAC	73	Chr8:1590035-1592301	DLGAP2
AGGACAGAGACGCGG	46	Chr8:625097-626540	ERICH1
CCCCCTGCACCCCCA	60	Chr8:940305-943540	DLGAP2
CCTCCACTGTGTGTG	98	Chr8:963588-967669	DLGAP2
TGTCACCGCGAGGGG	57	Chr8:983686-986261	DLGAP2
TGTGAGTGCATGTGG	45	Chr9:135252516-135254116	TTF1
ATCACACCTCCCAGG	70	Chr9:137740627-137742849	MIR
ACGGGACTACACGGG	61	Chr9:140223024-140225109	EXD3
CCCCCAGACCAGGAC	52	Chr9:140334277-140335713	ENTPD8
ACTCTGTCTGTGAGG	84	Chr9:140459676-140463065	DPH7
CCACCTGCCCTGATG	46	Chr9:140912342-140914057	CACNA1B
TGTGTGATGCTGGGG	78	Chr10:133972112-133975418	JAKMIP3
CCTGGGTTCTGTCTG	60	Chr10:134531001-134535345	INPP5A
CCCTGGATCACAGCC	48	Chr10:1582650-1585175	ADARB2
TCCCACTCCACCAGG	60	Chr10:1741356-1744133	ADARB2
AGATGAGCTTGGGGG	46	Chr11:134034437-134037905	NCAPD3
GAGCGTCTTCTGCGG	76	Chr11:1450754-1454390	BRSK2
CCCCGCTCACCCCCG	64	Chr11:400746-403054	PKP3
TCTGACCATGTCTGG	49	Chr11:410465-412766	SIGIRR
CACACATCAACATGG	68	Chr11:60866662-60869195	CD5
TATGCCTATGCCTGG	89	Chr12:1194126-1200023	ERC1
TGGGGTAGAAGGTGG	47	Chr12:122185156-122186161	TMEM120B
GGGGGTCTGTCCCGG	62	Chr12:124395812-124397056	DNAH10
CCTCATCCTCATCAC	59	Chr12:124951485-124953558	NCOR2
GAGTGAGTGGAGTGG	49	Chr12:125894105-125895755	TMEM132B
GAGTGTGTTTACTGG	46	Chr12:129130259-129133630	TMEM132C
TCCTCCATGACCGGG	52	Chr12:129572401-129574161	TMEM132D
CCAGGGGAACGGGAT	98	Chr12:130339606-130343633	TMEM132D
CCTTCCCTATACCCA	152	Chr12:132811643-132819399	GALNT9
CCTGGGCACACCCTC	65	Chr12:132879958-132882307	GALNT9
CCAGTTAGTTAGTGA	78	Chr12:133268545-133271869	PXMP2
CACCACCAGCACAGG	51	Chr12:3306816-3308072	TSPAN9
CCTGCAGCCTGCCCT	54	Chr12:3377306-3378778	TSPAN9
GACAGGGACAACTGG	62	Chr12:40876395-40885001	MUC19
CCACCTTCCACCTTC	102	Chr12:620386-622381	B4GALNT3
CCTGGTAAGCATGGA	397	Chr13:112930813-112973591	LINC01043
GGAGGACCTCTGTGG	54	Chr13:114000255-114002380	GRTP1
CCTCGCGGGGAAATC	70	Chr13:114848979-114852850	RASA3
GAATGTGAGTGACGG	60	Chr13:30131785-30133705	SLC7A1
CCCGTGGCACCCTCA	111	Chr14:105695963-105707283	BRF1
GCAGATGAGGGATGG	85	Chr15:101094498-101098864	PRKXP1
ACGGTGACAGCACGG	48	Chr16:1010255-1012281	LMF1
GGTGTCTCGGGATGG	66	Chr16:907422-909987	LMF1
CCTGAGCCTGAGTTC	74	Chr17:1020323-1022749	ABR
TACTTCCTATGAGGG	65	Chr17:103456-107807	RPH3AL
CCACCGTCAGACCTC	67	Chr17:136689-138746	RPH3AL
CCACCTCCATTGACC	61	Chr17:164188-167359	RPH3AL
ACACACCTCCACTGG	59	Chr17:26842241-26845271	FOXN1
CCCTCAGAACCTAAT	63	Chr17:478652-487334	VPS53
GGAGTTAGGGAGGGG	53	Chr17:77000014-77001370	CANT1
AGGTGATGGTGGTGG	73	Chr17:78287209-78289860	RNF213
CCGCACGGCAGCTCA	57	Chr17:78814075-78815874	RPTOR
CCTCAGTTTCCCTCA	75	Chr17:80317257-80319485	TEX19
CCGCGTGTGGGATTC	45	Chr17:80960763-80962730	B3GNTL1
CCTCCTCCTCTCCCC	45	Chr17:852048-852985	NXN
CCTTCCTCTATTGAT	55	Chr17:92037-94098	RPH3AL
TATGTGAACTGAGGG	48	Chr17:965418-968846	ABR
CCTGATTATTAACAT	67	Chr18:13673058-13676522	FAM210A
CCACACTGTGCCTTT	46	Chr18:44926826-44929361	MIR4527HG
GTGGTCAGGTGGGGG	79	Chr18:74104742-74106806	ZNF516
GAACCGAGGGAGAGG	51	Chr18:77679453-77681928	SLC66A2

Repetitive sequence (5'-3')	Repeats	Genomic region (hg19)	Gene
CCAGGTCTGGTCATT	99	Chr19:54422568-54428888	CACNG7
GTGTAATGTCCGAGG	49	Chr19:56361126-56363117	NLRP4
CCTGCTTCCCTCCTC	77	Chr19:59050388-59054262	ZBTB45
GGTCACACTGAGGGG	64	Chr20:20335249-20338937	CFAP61
TGTGCAGTTTGGGGG	49	Chr20:59847681-59850791	CDH4
CCATTTCTAGAAACA	84	Chr20:62238367-62241366	GMEB2
GGGGGCAGTGTGGGG	57	Chr20:62724838-62725982	OPRL1
CCCTCCCTGGCTTCT	49	Chr20:62804793-62807841	MYT1
CCTGCTGAGGGAGAT	59	Chr21:45001698-45004129	HSF2BP
TGTGGAGGGGTGTGG	56	Chr21:45815540-45816849	TRPM2
CCCTGCACCAACACA	63	Chr22:44508553-44511878	PARVB
CCTGTGTGTAGGTTC	46	Chr22:47010646-47013453	GRAMD4
CCTCCAGTCAGTGCC	72	Chr22:50782053-50784952	PPP6R2
CGCGGGGTGAGCGGG	93	ChrX:1484145-1486728	IL3RA
AGAGAGATGGAGAGG	63	ChrX:2194791-2200434	DHRSX
CCAACGGGAGGCGGG	45	ChrX:303018-305087	PPP2R3B
CGCGGGGTGAGCGGG	93	ChrY:1434145-1436728	IL3RA
AGAGAGATGGAGAGG	63	ChrY:2144791-2150434	DHRSX
CCAACGGGAGGCGGG	45	ChrY:253018-255087	PPP2R3B

Table S2. Plasmids deposited with Addgene, related to STAR Methods.

Plasmid	Addgene ID
dCas9-10xSunTag(22aa)	215745
scFv-IFP2.0N	215746
MCP-YPet	215747
MBD-IFP2.0C	215748
MBD_R44Q-IFP2.0C	215749
2xHP1bCD-IFP2.0C	215750
2xHP1bCD_W42A-IFP2.0C	215751
2x3APWWP-IFP2.0C	215752
2x3APWWP_K299E_D333A-IFP2.0C	215753
2xCBX7CD-IFP2.0C	215754
2xCBX7CD_W35A-IFP2.0C	215755

Table S3. Compilation of genomic targets, their local copy numbers and sgRNAs used in this study, related to STAR Methods.

Target locus	Local copies	Chromosome	sgRNA sequence
α-satellites	> 1000	9	TGGAATGGAATGGAATGGAA
TTC34	392	1	GATGCTCACCTG
X(187)	187	X	CAAGGCAAGGCA
PR1	160	19	GTGACAGTGAAC
C19-2	45	19	AGCAGATGTAGG
KDM	28	19	CACTCCGATAAA
FBN3	22	19	ATCCCTCCAACC
TDRD	21	19	GACTGGCTGATG
scrambled	-	-	GAACAGTCGCGTTTGCGACT

Table S4. Plasmids used for transient transfection of BiAD sensors and detector validation, related to STAR Methods.

Plasmid	Amount [ng]
sgRNA	400
dCas9-SunTag	100
scFv-IFP2.0N/mVenusN	300
Detector-IFP2.0C/mVenusC	300
MCP-YPet/NLS-mRuby2-C1 (transfection control)	400
Detector-mVenus	200
Detector-dsRed (optional)	200
pcDNA4.1 (filler plasmid for detector validation)	add 1500

Table S5. Antibodies used in this study, related to STAR Methods.

Antibody	Company	Catalog No	Dilution
α-H3K36me3	Abcam	ab9050	1:500
α-H3K27me3	Active Motif	39155	1:500
α-H3K27me2/3	Active Motif	39536	1:750
α-H3K9me3	Diagenode	C15410193	1:500
goat α-rabbit IgG Alexa Fluor™ 405	Invitrogen	A-48258	1:750
goat α-mouse IgG Alexa Fluor™ 405	Invitrogen	A-31553	1:750
goat α-mouse IgG Alexa Fluor™ 594	Invitrogen	A-11005	1:2000
goat α-mouse IgG Alexa Fluor™ 633	Invitrogen	A-21052	1:2000
goat α-rabbit IgG Alexa Fluor™ 647	Invitrogen	A-32733	1:2000

Table S6. siRNAs used in this study, related to Figure 6.

Knockdown target	Ambion silencer select assay ID
siNT (negative control)	#4390843
GLP (EHMT1)	s36392
G9a (EHMT2)	s21469
SETDB1	s19112
SUV39H1	s13658
SUV39H2	s36183

Table S7. Primer sequences used for RT-qPCR siRNA knockdown validation and 5mC-sensitive qPCR, related to Figure 2 and Figure 6.

H3K9 methyltransferase	forward primer (5' to 3')	reverse primer (5' to 3')
GLP (EHMT1)	CAGGACTTCCAAGGAGAGCA	ACTCAGGTCAGACTCGTCAC
G9a (EHMT2)	AGTGATGATGTCCACTCACTGGGA	AGAGACTGAAGTCATCACCCACCA
SETDB1	GCCTACAGCAAGGAACGTATCC	GTTGATGGCAGGCACACTTGGA
SUV39H1	GCCCAAATCGTGTGGTACAG	CCTGACGGTCGTAGATCTGG
SUV39H2	ATTGATAACCTCGATACTCGTCTT	TCTCCAGAACCTTTCATTTGATAA
SDHA (housekeeping gene)	TGGGAACAAGAGGGCATCTG	CCACCACTGCATCAAATTCAT
Amplicon 1	ACCATCCACCACTGGTTCAC	CCCACTGTGGTGGTAGAGTA
Amplicon 2	AAGCTGTTCCGTGTTAGCC	TGAAGAGGTTCCATCGTGGT
Control amplicon	CCCACTCGTTCTCCGAAACA	TGGATGGCAGTGTTGTGGTT
