# Supplementary material for: Modular dual-color BiAD sensors for locus-specific readout of epigenome modifications in single cells
Source: Cell Rep Methods. 2024 Mar 29;4(4):100739. doi: 10.1016/j.crmeth.2024.100739 (PMC11045877; doi:10.1016/j.crmeth.2024.100739)
Supplement: Document S2. Article plus supplemental information [file mmc2.pdf]

Cell Reports
Methods

Article

Modular dual-color BiAD sensors for locus-specific readout of epigenome modifications in single cells

Graphical abstract

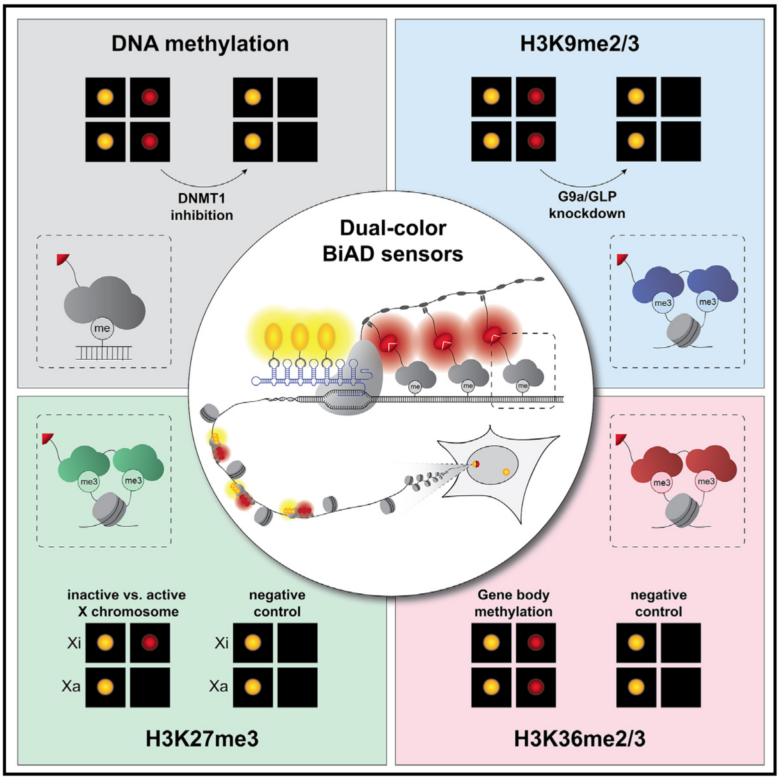

Highlights

- Dual-color BiAD sensors combine CRISPR imaging with epigenome modification readout
- Locus-specific detection of DNA, H3K9, H3K27, and H3K36 methylation in live cells
- Quantitative analysis of epigenome dynamics upon DNMT inhibitor treatment
- Investigation of cellular effects of H3K9 methyltransferase knockdown

Authors

Anja R. Köhler, Johannes Haußer, Annika Harsch, ..., Monilola A. Olayioye, Pavel Bashtrykov, Albert Jeltsch

Correspondence

albert.jeltsch@ibtb.uni-stuttgart.de

In brief

Bimolecular anchor detector (BiAD) sensors enable locus-specific investigation of epigenome modifications in live cells. Köhler et al. develop dual-color BiAD sensors combining the BiAD detection with CRISPR imaging for improved sensitivity and introduce detectors for dynamic readout of DNA, H3K9, H3K27, and H3K36 methylation at human endogenous target loci.

Köhler et al., 2024, Cell Reports Methods *4*, 100739
April 22, 2024 © 2024 The Author(s).
https://doi.org/10.1016/j.crmeth.2024.100739

CellPress

Cell Reports Methods

CellPress
OPEN ACCESS

Article

Modular dual-color BiAD sensors for locus-specific readout of epigenome modifications in single cells

Anja R. Köhler,[1] Johannes Haußer,[1] Annika Harsch,[1] Steffen Bernhardt,[1] Lilia Häußermann,[1] Lisa-Marie Brenner,[2] Cristiana Lungu,[2] Monilola A. Olayioye,[2] Pavel Bashtrykov,[1] and Albert Jeltsch[1,3,*]

[1]Institute of Biochemistry and Technical Biochemistry, University of Stuttgart, Allmandring 31, 70569 Stuttgart, Germany

[2]Institute of Cell Biology and Immunology, University of Stuttgart, Allmandring 31, 70569 Stuttgart, Germany

[3]Lead contact

*Correspondence: albert.jeltsch@ibtb.uni-stuttgart.de

https://doi.org/10.1016/j.crmeth.2024.100739

MOTIVATION Bimolecular anchor detector (BiAD) sensors enable the detection of epigenome modifications with locus-specific resolution in live cells. However, their application in human cells has so far been limited to DNA methylation readout at highly repetitive genomic regions. To improve the sensitivity, we aimed to develop dual-color BiAD sensors by combining CRISPR imaging for visualization of the specific genomic target locus with the BiAD readout of the epigenome modifications at this site. Thus, the BiAD analysis could be focused on the target locus region, allowing us to disregard signals in other parts of the nucleus, which was expected to increase the sensitivity of the method strongly. Moreover, we aimed to integrate additional double chromatin reader domains as detector modules into the system to increase its applicability for the readout of important histone posttranslational modifications in human cells.

SUMMARY

Dynamic changes in the epigenome at defined genomic loci play crucial roles during cellular differentiation and disease development. Here, we developed dual-color bimolecular anchor detector (BiAD) sensors for high-sensitivity readout of locus-specific epigenome modifications by fluorescence microscopy. Our BiAD sensors comprise an sgRNA/dCas9 complex as anchor and double chromatin reader domains as detector modules, both fused to complementary parts of a split IFP2.0 fluorophore, enabling its reconstitution upon binding of both parts in close proximity. In addition, a YPet fluorophore is recruited to the sgRNA to mark the genomic locus of interest. With these dual-color BiAD sensors, we detected H3K9me2/3 and DNA methylation and their dynamic changes upon RNAi or inhibitor treatment with high sensitivity at endogenous genomic regions. Furthermore, we showcased locus-specific H3K36me2/3 readout as well as H3K27me3 and H3K9me2/3 enrichment on the inactive X chromosome, highlighting the broad applicability of our dual-color BiAD sensors for single-cell epigenome studies.

INTRODUCTION

The dynamic regulation of gene expression during embryonic development and cellular differentiation is governed by a complex network of epigenetic mechanisms.[1–3] All of these processes require the precise spatiotemporal establishment and maintenance of defined patterns of epigenome modifications, such as DNA methylation and numerous histone posttranslational modifications (PTMs).[4–6] Conversely, disruptions in these locus-specific epigenomic patterns have been linked to the onset and progression of a wide spectrum of human diseases, including cancer.[7–10] Adding to the complexity, it has become increasingly evident that many physiological and pathophysiological processes exhibit a high cell-to-cell heterogeneity.[11–13] Therefore, gaining a comprehensive understanding of the locus-specific dynamics of epigenome modifications at single-cell resolution is essential to unravel the regulatory mechanisms underlying these processes, connect them to cellular phenotypes, and develop effective treatment strategies.[13,14]

Recent advancements in epigenomic sequencing technologies, such as DNA methylation analysis by bisulfite conversion coupled to sequencing and analysis of histone PTMs by various types of chromatin immunoprecipitation followed by high-troughput sequencing (ChIP-seq), including single-cell approaches, have significantly enhanced our understanding of the epigenome in both health and disease.[15,16] However, these techniques can only provide a snapshot of the epigenomic landscape at a particular time point, and they cannot be used to

This is an open access article under the CC BY license (http://creativecommons.org/licenses/by/4.0/).

match epigenome modifications and cellular phenotypes because the procedures require cell lysis. To address this limitation, bimolecular anchor detector (BiAD) sensors were developed for locus-specific readout of epigenome modifications in single cells.[17] The BiAD technology is also compatible with live-cell imaging, potentially allowing the analysis of dynamic changes of epigenome modifications at the level of individual cells. The BiAD sensors comprise a programmable DNA-binding anchor module, such as engineered zinc finger proteins, transcription activator-like effectors, or a single-guide RNA (sgRNA)/dCas9 (dead Cas9) complex for locus-specific targeting of the sensor, and different chromatin reader domains used as detector modules for the recognition of defined chromatin modifications. Both modules are fused to complementary parts of a split fluorophore, enabling bimolecular fluorescence complementation (BiFC) when both modules bind in close spatial proximity. However, the imaging-based readout requires a strong BiFC signal at the target locus to distinguish it from the nuclear background, fluorophore aggregation, and signal fluctuations. Hence, the application of BiAD sensors so far was limited to highly repetitive genomic loci with >1,000 local repeats,[17] which lead to the binding of several BiAD sensors at the target locus. Thus, enhancing the sensitivity of the BiAD sensors was essential to overcome this limitation and enable their application to a broader range of biologically relevant endogenous target regions.

Over the last years, several dCas9-based signal amplification systems have been developed for the visualization of endogenous genomic loci in live cells. This can be achieved by using a dCas9-fused peptide scaffold, like the SunTag, which enables the binding of multiple antibody-fused fluorophores per sgRNA/dCas9 binding site.[18] Another frequently used method is to employ modified sgRNAs, such as MS2-based amplification systems[19,20] or the Casilio system,[21,22] which specifically recruit multiple fluorescently labeled RNA-binding proteins to the sgRNA/dCas9 target locus. Although these systems are already applied in CRISPR imaging for locus-specific genomic visualization of repeats with lower local copy numbers and, in some cases, even at nonrepetitive loci, they do not provide information on the epigenomic state at the target locus.

In this study, we combined CRISPR imaging for the visualization of specific genomic loci with the BiAD sensor-based readout of chromatin modifications at the endogenous target sites, using two distinct sgRNA/dCas9-based signal amplification systems. This dual-color readout drastically enhanced the sensitivity of BiAD sensors, enabling the qualitative and quantitative locus-specific detection of chromatin modifications at regions with low local copy numbers in single cells by fluorescence microscopy. Furthermore, we integrated improved detector modules for different epigenome modifications by using double chromatin reader domains for readout of histone 3 lysine 9 di- and trimethylation (H3K9me2/3), H3K27me3, and H3K36me2/3 in human cells, in addition to the already-established detector module for DNA methylation based on the MBD domain of MBD1. With its improved sensitivity and diverse panel of detector modules, these modular dual-color BiAD sensors offer a versatile and powerful tool to investigate locus-specific changes in chromatin modifications at a broad range of endogenous target sites in live cells. Our sensors allow the readout of epigenome modifications to be directly coupled with cellular phenotypes and cell physiology. These new developments are expected to provide important insights into epigenomic signaling pathways at the single-cell level.

RESULTS

In our previous work, different BiAD sensors were established for locus-specific detection of epigenome modifications in living cells.[17] This included dCas9-based BiAD sensors for DNA cytosine-C5 methylation (5mC) readout, using the N-terminal part of split mVenus fused to the dCas9 anchor module and the C-terminal part of mVenus fused to the MBD domain of MBD1. This enabled BiFC when both modules bound to chromatin in close spatial proximity. The design of these sensors allowed for the reconstitution of one mVenus fluorophore per dCas9 binding site, if 5mC was present at the target locus. Although this approach was successfully applied for the detection of 5mC at highly repetitive centromeric and pericentromeric sequences in mouse and human cells with >1,000 local repeats, preliminary work in our lab showed that it was not functional at target loci with a smaller number of local repeats because fewer reconstituted fluorophores made it difficult to detect the BiFC signal.

Dual-color BiAD sensor improves sensitivity of 5mC detector

To enhance the sensitivity of the 5mC BiAD sensor for readout of epigenome modifications at repeat loci with lower local copy numbers, we first aimed to amplify the fluorescent signal. For this, we added a 10×SunTag to the dCas9 and fused the N-terminal part of split mVenus to a single-chain fragment variable (scFv) antibody fragment that specifically binds the GCN4 epitopes of the SunTag. This system enables the recruitment of multiple detector modules to a single sgRNA/dCas9 binding site, because in principle each N-terminal mVenus fragment can reconstitute a full fluorophore. The performance of the improved BiAD sensor was analyzed using a calibration set of genomic loci containing repeats with different local copy numbers between 45 and >1,000 taken from Ma et al.[20] (Figure 1A). However, the resulting signal amplification was still insufficient to reliably discriminate BiFC signals at target regions with <1,000 repeats from an unspecific nuclear background, like fluorophore aggregation or noise (Figure S1A). To overcome this limitation, we explored the possibility of using an additional detection system to recruit a full fluorophore to the same place and by this visually mark the target locus and then allow specific readout of the BiAD signal only at this region. Using the CRISPR-Sirius MS2-based amplification system,[20] we could reliably detect regions with as few as 45 local repeats in HEK293 cells using the bright YPet fluorophore[23] as detector (Figure S1B). Depending on the target locus, we observed two to four distinct YPet spots, which in each case was in line with the known polyploidy of HEK293 cells.[24]

After evaluating several split fluorophores, we chose the near-infrared fluorophore IFP2.0 because it was previously used for reversible BiFC assays,[25,26] and it does not overlap spectrally with the YPet marker signal. IFP2.0 was split at position 132 to create two nonfluorescent parts. The N-terminal part was fused

CellPress
OPEN ACCESS

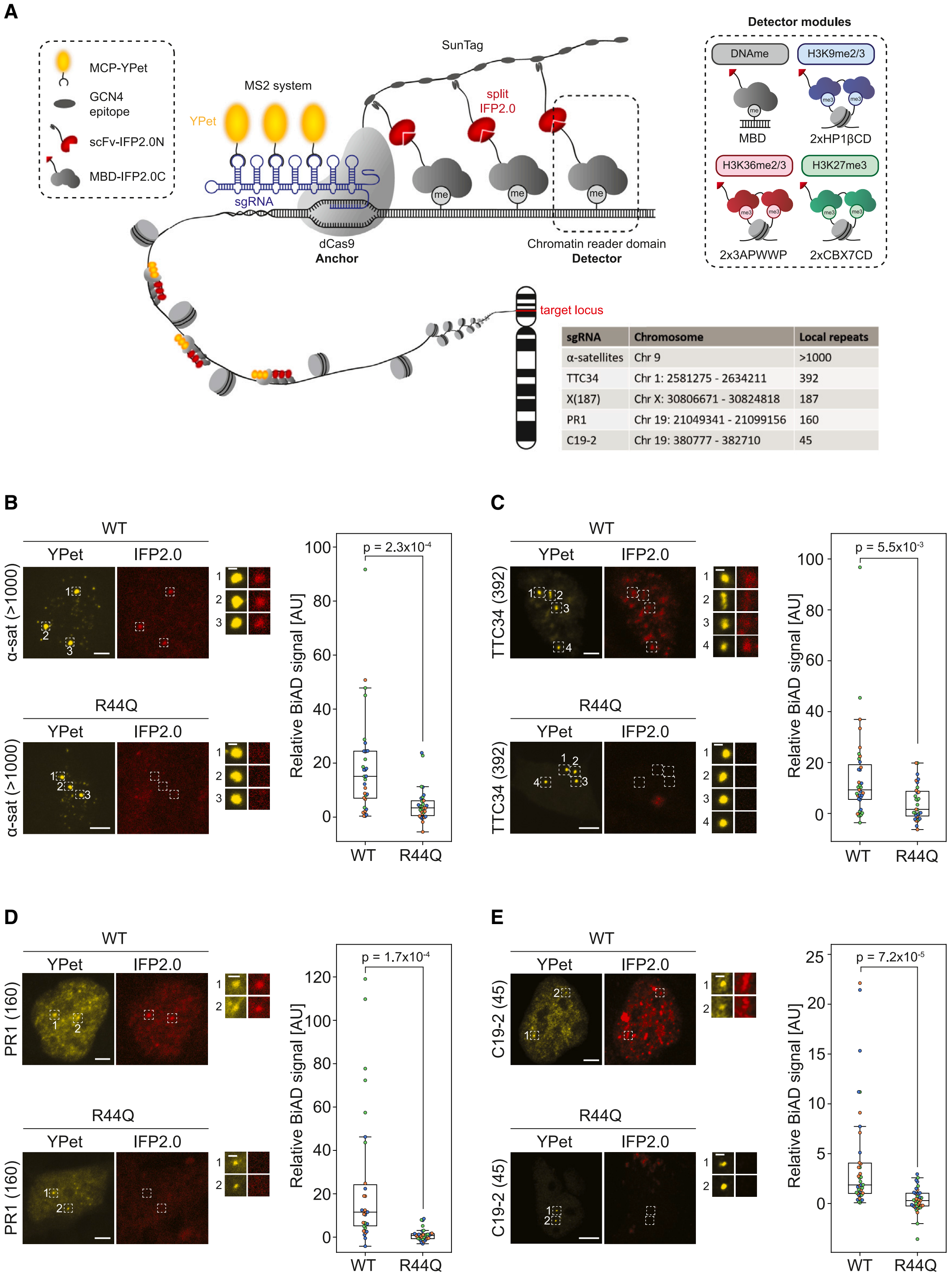

Figure 1. Validation of the dual-color BiAD sensor for 5mC readout

(A) Schematic representation of the modular dual-color BiAD sensor and compilation of selected target loci with decreasing local repeat copy numbers. The target locus is visualized by a full-length fluorophore (YPet), which is recruited by an MS2 scaffold of the sgRNA in complex with the anchor module (dCas9). One part of the split fluorophore (IFP2.0) is recruited to the SunTag amplification system fused to dCas9 by an scFv antibody. If the chromatin modification of interest is present at the target locus, then the detector module (chromatin reader domain) can bind and bring the second part of the split IFP2.0 in close spatial proximity,

(legend continued on next page)

to the scFv antibody fragment binding to the SunTag, and the C-terminal part was fused to the detector module. This dual-color BiAD sensor combines the visualization of a specific genomic locus in the YPet channel with the BiAD sensor-based readout of chromatin modifications at the target locus in the IFP2.0 channel (Figure 1A). Moreover, the YPet marker fluorophore not only enables the precise localization of the dCas9 binding site but it also serves as an independent normalization marker for size, condensation, and accessibility of the target locus facilitating the quantitative analysis of the IFP2.0 BiAD signal at the target locus (Figure S1C).

We first applied the sensor with the MBD1 detector module for the investigation of DNA methylation at the genomic loci from the calibration set, revealing colocalization of the YPet signal with the reconstituted IFP2.0 BiAD signal for the wild-type (WT) MBD1 detector, but not for the 5mC binding-deficient R44Q mutant, which was used as a negative control.[17,27] Control experiments lacking the scFv-IFP2.0N component did not reveal noticeable BiAD signals, confirming the specificity of our sensor (Figure S2A). With this dual-color BiAD sensor, we successfully detected 5mC at loci with decreasing local copy numbers, ranging from highly repetitive α-satellites (>1,000 repeats) to as few as 45 repeats at the C19-2 locus, indicating a drastic increase in sensitivity (Figures 1B–1E). In particular, in the case of the low copy-number repeats, based on the IFP2.0 BiAD signal alone, it was not possible to distinguish the signal at the locus of interest from unspecific nuclear background signals, emphasizing the crucial need for a second, independent fluorophore marking the endogenous target site that is a key advantage of our dual-color BiAD sensors. Since we did not observe adverse effects of cellular fixation on the BiAD and YPet signals (Figure 1D) compared to live-cell imaging as exemplarily shown for the PR1 (160 repeats) locus (Figure S2B), we proceeded with fixation of the transfected cells for more convenient handling and more flexible experimental design and allocation of instrument time at the microscope, and to allow direct comparison of BiAD images with immunofluorescence analyses.

For the quantitative analysis of the BiAD signal at the target locus, two intensity thresholds in the YPet channel were used—one to define the nucleus and the other to define high-intensity YPet spots as regions of interest (ROIs). Subsequently, the mean signal intensities in the YPet and IFP2.0 channels were measured for nucleus and spot ROIs. After subtracting the respective mean nuclear intensities, the IFP2.0 BiAD spot intensity was normalized to the corresponding YPet spot intensity. The resulting normalized BiAD signal was averaged for all of the spots within a single cell (for a detailed explanation, refer to the STAR Methods section and Figure S1C). The analysis of at least 30 cells with WT and R44Q negative control detector revealed a highly significant number of WT cells with a strong BiAD signal for all of the tested target loci (Figures 1B–1E). An exemplary analysis of the PR1 (160 repeats) locus data did not show noticeable differences in the YPet spot area or the spot count per nucleus for cells transfected with WT or R44Q detector (Figures S2C and S2D). For the purposes of this study, we deliberately chose to use transient transfections because they facilitate the flexible interchange of different modules to adjust to various experimental setups. However, it must be noted that cells had to be transiently transfected with five plasmids encoding all of the components of the dual-color BiAD sensor, but selection for imaging and analysis was based only on the presence of YPet spots. Defined YPet spots indicated the successful transfection and expression of three components: dCas9-SunTag, MCP-YPet, and the sgRNA, whereas the detector and the scFv modules, which carry the nonfluorescent IFP2.0 parts, cannot be accounted for. Hence, lack of sufficient amounts of these plasmids in some of the transfected cells may explain the occurrence of cells with no apparent BiAD signal even in settings where positive BiAD signals are expected. Therefore, the potential presence of cells incapable of generating a BiAD signal must be considered for the interpretation of all of the results.

Detection of dynamic changes of DNA methylation in living cells with the dual-color BiAD sensor

To determine whether the dual-color BiAD sensor can be used to track locus-specific dynamic changes in 5mC levels, we investigated 5mC in HEK293 cells upon treatment with the GSK-3685032 DNA methyltransferase 1 inhibitor (DNMT1i).[28] Therefore, we transfected HEK293 cells with all of the components of the dual-color BiAD sensor for 5mC readout and treated them either with GSK-3685032 DNMT1i or with DMSO (mock control) (Figure 2A). Three days after treatment, global DNA demethylation at CpG sites in the DNMT1i-treated cells was verified by MspI (5mCpG insensitive) and HpaII (5mCpG sensitive) restriction digestion of purified genomic DNA (Figure 2B). Moreover, demethylation at the PR1 locus was confirmed by restriction digestion analyzed by qPCR at two exemplary sites (Figure S2E). Subsequently, using the dual-color BiAD sensors to assess locus-specific 5mC changes, we observed drastically reduced BiAD signals at α-satellite (>1,000 repeats) and PR1 (160 repeats) regions after 3 days of inhibitor treatment in comparison to mock-treated cells (Figures 2C and 2D). These findings document the applicability of the dual-color BiAD

leading to reconstitution of the split IFP2.0. Detector modules used for the recognition of DNA methylation and histone posttranslational modifications were the MBD1 MBD domain for DNA methylation and double domains (2×) of the HP1β CD for H3K9me2/3, DNMT3A PWWP domain for H3K36me2/3, and CBX7CD for H3K27me3.

(B–E) HEK293 cells were transfected with all components of the dual-color BiAD sensor for 5mC detection with either the WT MBD1 detector or a 5mC binding-deficient mutant (R44Q). The BiAD sensor was tested on different target loci with decreasing local repeat numbers: (B) α-satellites (>1,000 repeats), (C) TTC34 (392 repeats), (D) PR1 (160 repeats), (E) and C19-2 (45 repeats). Exemplary fluorescence microscopy images showing the nucleus of individual fixed cells with colocalization of the marker (YPet) and BiAD signal (IFP2.0) for the WT detector, but not for the binding-deficient R44Q mutant. Scale bars, 5 μm and 1 μm for the magnified images. The boxplots show the relative BiAD signals. Each dot represents the mean relative BiAD signal of all of the spots within a single cell. Data were obtained in 3 independent experiments (depicted in orange, blue, and green). Significance was determined via a 2-tailed, unpaired t test. The p values are indicated in each boxplot.

See also Figure S2.

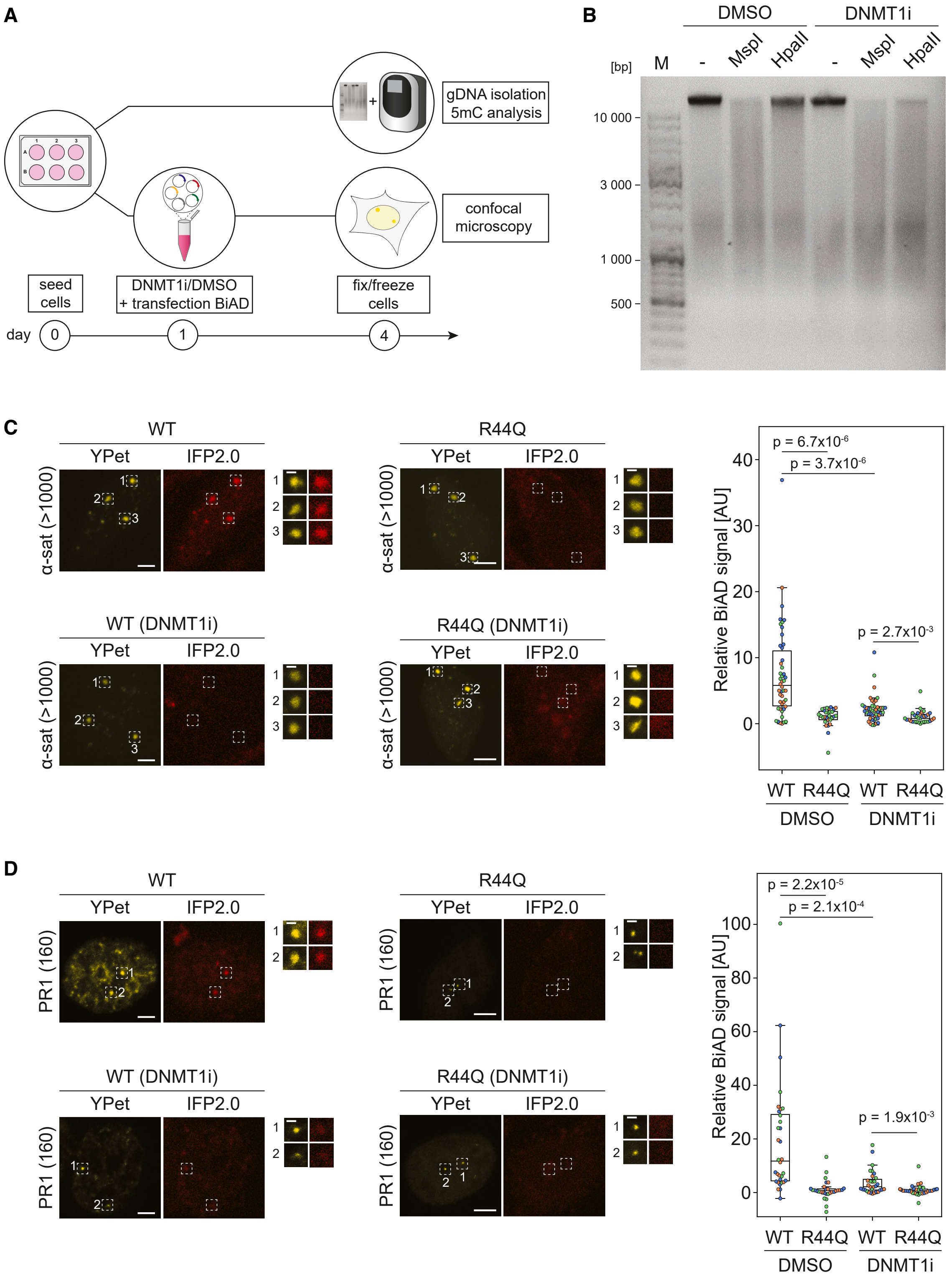

Figure 2. Visualization of locus-specific 5mC loss upon GSK-3685032 DNMT1i treatment

(A) Schematic representation of the experimental setup. HEK293 cells were transfected with all components of the dual-color BiAD sensor for 5mC detection with either the WT MBD1 detector or a binding-deficient mutant (R44Q). Starting at the day of transfection, cells were treated with the DNMT1i or mock treated (DMSO) for 3 days.

(legend continued on next page)

5mC sensor to monitor changes in DNA methylation levels at endogenous genomic loci upon DNMT1i treatment by fluorescence microscopy in single cells.

Double detector domains enhance the detection of histone PTMs in cells

In addition to the DNA methylation readout, our goal was to broaden the range of detector modules and allow the investigation of additional histone PTMs with our dual-color BiAD sensors. Previously, we used the chromodomain (CD) of HP1β (CBX1) for detection of H3K9me2/3 at mouse major satellite repeats.[17] However, this detector displayed low sensitivity in human cell lines. Based on recent studies and our own experience,[29–33] we hypothesized that the binding affinity and specificity of the detector modules toward histone PTMs could be improved by using two fused domains that could benefit from avidity effects in their specific chromatin interaction with two modified histone tails at the target locus (called double domains henceforth). We, therefore, evaluated single and double domains (2×) of HP1βCD for H3K9me2/3, the PWWP domain of DNMT3A (3APWWP) for H3K36me2/3 and the CBX7 chromodomain (CBX7CD) for H3K27me3 readout.

Pilot experiments to assess the detection of the H3K9me2/3 and H3K36me2/3 modifications were conducted in NIH3T3 cells because immunofluorescence staining for both histone PTMs displayed distinct subnuclear patterns in these cells. Comparison of NIH3T3 cells transfected with single and double (2×) HP1βCD domains fused to mVenus revealed the previously observed heterochromatic pattern of the mVenus signal colocalizing with DAPI and H3K9me3 staining in mouse cells for the WT reader domain.[17] However, the double domain displayed an even more pronounced spotty phenotype, indicating enhanced H3K9me2/3 binding (Figure 3A). Cells transfected with the binding pocket mutant W42A did not display a spotty pattern for both single and double domains. To validate these findings, we performed cotransfections of WT and W42A double domains in NIH3T3 as well as HEK293 cells (Figure S3A). By fusing WT and mutant double domains to different fluorophores (WT-mVenus/W42A-dsRed), we could observe their respective localization at the same time within the same cell. To exclude an adverse effect of the fused fluorophore on the binding of the detector modules, we first confirmed colocalization of WT-mVenus and WT-dsRed double domains with H3K9me3-dense, heterochromatic foci in the same cell. However, this pattern was not detected for cotransfections of WT-mVenus with W42A-dsRed, thus confirming that the specific H3K9me2/3 binding was lost with the W42A mutant.

For the 3APWWP domain, we once again observed that the double domain provided an advantage for H3K36me2/3 detection because it showed colocalization with H3K36me3 antibody-stained heterochromatic foci in NIH3T3, whereas the single domain did not (Figure 3B). The binding-deficient K299E mutant[34] did not display colocalization with H3K36me3 or DAPI staining. These results were confirmed by cotransfection experiments with 2×3APWWP (WT-mVenus/dsRed and WT-mVenus/K299E-dsRed) in the same cell (Figure S3B).

Evaluation of CBX7CD single and double domain binding was carried out in HEK293 cells, which exhibit robust H3K27me3 staining at the inactive X chromosome(s) (Xi). For H3K27me3 detection, transfections with single CBX7CD revealed a diffuse subnuclear signal with no visible colocalization with the H3K27me3 immunostaining, whereas the double domain (2× CBX7CD) clearly colocalized with Xi based on the H3K27me3 staining (Figure 3C). The binding-deficient W35A mutant[35] did not show this colocalization. This specific loss of H3K27me3 binding by the W35A mutant was also validated by cotransfection experiments (Figure S3C). Since previous *in vitro* studies reported binding of the CBX7CD to H3K27me3 as well as H3K9me3,[36] we cotransfected 2×HP1βCD and 2×CBX7CD into NIH3T3 and HEK293 cells and conducted immunostaining for H3K9me3 and H3K27me3 (Figure S3D). The Xi in HEK293 cells showed strong H3K9me3 and H3K27me3 staining, which was reflected by colocalization with 2×HP1βCD as well as 2× CBX7CD. In contrast, the H3K9me3-dense foci in NIH3T3 and HEK293 cells did not colocalize with 2×CBX7CD-mVenus, indicating preferred binding of 2×CBX7CD to H3K27me3 in the cellular context.[31] These initial experiments indicated that the use of double domains, which enable multivalent interactions with the chromatin at the target site, improved the recognition of histone PTMs in all three cases.

Validation of additional detector modules for locus-specific H3K27me3 and H3K36me2/3 readout in dual-color BiAD sensors

To assess the potential of the 2×CBX7CD detector module as dual-color BiAD sensor for H3K27me3 readout, we selected the X(187) region on the X chromosome with 187 local repeats, since the Xi in female cells is characterized by enriched H3K27me3.[37] In our preliminary experiments (Figure 3C), we observed that the Xi forms a visible Barr body in most cells, which is characterized by dense DAPI staining. However, many other heterochromatic regions also display elevated DAPI signals, which is why we chose to use H3K27me3 staining for identification of the Xi in our experiments to clearly distinguish it from other heterochromatic regions. Therefore, we additionally immunostained cells with an H3K27me3 antibody to identify the inactive (Xi) X chromosome(s). The sensor typically detected three spots in HEK293 cells in the YPet channel, which agrees with the polyploidy of this cell line. In most cells, one or two of the spots overlapped with H3K27me3 staining, indicating that these

(B) Agarose gel showing global loss of CpG methylation in genomic DNA upon DNMT1i treatment. Equal amounts of genomic DNA isolated from DNMT1i/DMSO-treated cells were digested at CCGG sites with MspI (5mCpG insensitive) or HpaI (5mCpG sensitive).
(C and D) Representative fluorescence microscopy images of fixed cells showing the loss of 5mC BiAD signal at (C) α-satellites (>1,000 repeats) and (D) PR1 (160 repeats) upon DNMT1i treatment. Scale bars, 5 μm and 1 μm for the magnified images. Boxplots show the relative BiAD signal intensities of DNMT1i- and mock-treated cells normalized to the respective MBD1 R44Q mutant. Data were obtained in 3 independent experiments (depicted in orange, blue, and green). Significance was determined via a 2-tailed, unpaired t test. The p values are indicated in each boxplot.
See also Figure S2.

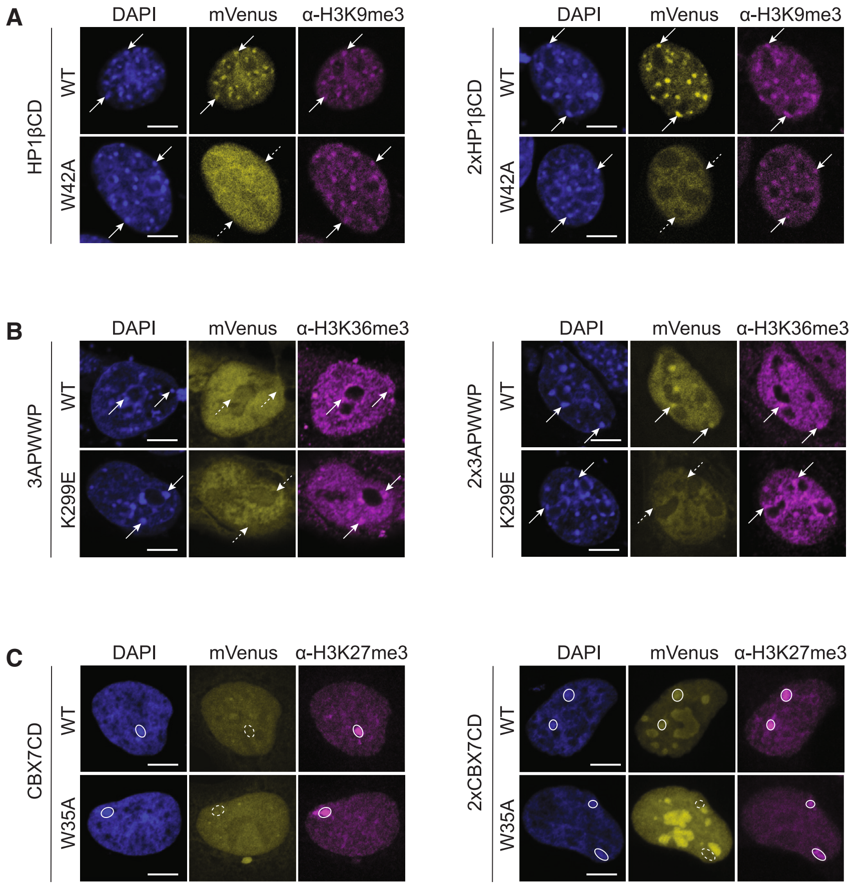

Figure 3. Validation of single and double reader domains as detector modules for histone PTMs

(A) Representative fluorescence microscopy images of fixed NIH3T3 cells transfected with mVenus-fused HP1βCD single or double domain (2×) and the corresponding W42A binding pocket mutants. The WT 2×HP1βCD colocalizes with H3K9me3 staining at DAPI-dense heterochromatic regions, as exemplarily indicated by arrows. Dotted arrows indicate missing colocalization of the respective W42A mutants.

(B) Representative fluorescence microscopy images of fixed NIH3T3 cells transfected with mVenus-fused DNMT3A PWWP single (3APWWP) or double domain (2×) and the corresponding K299E binding pocket mutants. The WT 2×3APWWP domain colocalizes with H3K36me3 immunostaining at DAPI-dense heterochromatic regions, as exemplarily indicated by arrows. Dotted arrows indicate missing colocalization of the single domain and the respective K299E mutants.

(C) Representative fluorescence microscopy images of fixed HEK293 cells transfected with mVenus-fused CBX7CD single or double domain (2×) and the corresponding binding-deficient W35A mutants. 2×CBX7CD WT colocalizes with H3K27me3 and DAPI staining at the Xi, as indicated by circles. Dotted circles indicate missing colocalization of the single domain and the respective W35A mutants.

In all of the panels, the images are representative of 5–15 individual cells that were analyzed.

See also Figure S3.

loci represent an Xi. Comparison of the relative BiAD IFP2.0 signals obtained with the WT 2×CBX7CD detector revealed a stronger signal on the Xi relative to the Xa (Figures 4A and 4B). This indicates that the dual-color BiAD sensor successfully detects the enrichment of H3K27me3 at the Xi. In contrast, the binding-deficient detector module 2×CBX7CD W35A did not show any BiAD signal, further confirming the specificity of the sensor for H3K27me3 detection.

To assess the potential of 2×3APWWP as the dual-color BiAD detector module, a repetitive region in a gene body (TTC34, 392 repeats) was selected because gene bodies of expressed genes are known to carry extensive H3K36me3.[38] Transfections of the dual-color BiAD sensor for H3K36me2/3 readout at the TTC34 locus with the 2×3APWWP detector module revealed colocalization of the YPet marker signal and the BiAD IFP2.0 signal at the target locus in HEK293 cells (Figures 4C and 4D). However, we still observed some cells with an IFP2.0 signal at the target locus with the K299E binding pocket mutant, indicating insufficient performance of the negative control most likely due to residual binding of the mutated detector module. To address this issue, we tested two double mutants of the detector domain, K299E/D333A and K299E/R366E, because previous work from our laboratory showed the importance of the two residues D333 and R366 for H3K36me2/3 and DNA binding, respectively.[34] Indeed, the K299E/D333A double mutant effectively eliminated the unspecific IFP2.0 signal, indicating its attribution to residual H3K36me2/3 rather than DNA binding (Figures 4C and 4D). These results demonstrate that the 2×CBX7CD and 2×3APWWP domains and their respective binding-deficient mutants can be used as detector modules in dual-color BiAD sensors to visualize H3K27me3 and H3K36me2/3 at endogenous target sites in live cells.

Detection of H3K9me3 on Xi using the dual-color BiAD system

After our pilot experiments indicated enhanced H3K9me2/3 binding of HP1βCD as a double domain (2×HP1βCD) in NIH3T3 cells (Figure 3A), we wanted to explore its potential as a detector module in dual-color BiAD sensors to locus-specifically investigate H3K9me2/3 in human cells. Using the double domain detector (2×HP1βCD), we were able to visualize H3K9me2/3 at repetitive endogenous loci in human cells for the first time (Figures 5A–5D). Similar to 5mC, we could detect H3K9me2/3 BiAD signals at various repeat numbers from α-satellites (>1,000 repeats) to 45 repeats at the C19-2 region.

To further test the H3K9me2/3 double domain detector module, we investigated whether it could be used to distinguish allelic differences in this histone PTM. Therefore, we used the intergenic X(187) region, which was already used for validation of the H3K27me3 detector, to determine whether our H3K9me2/3

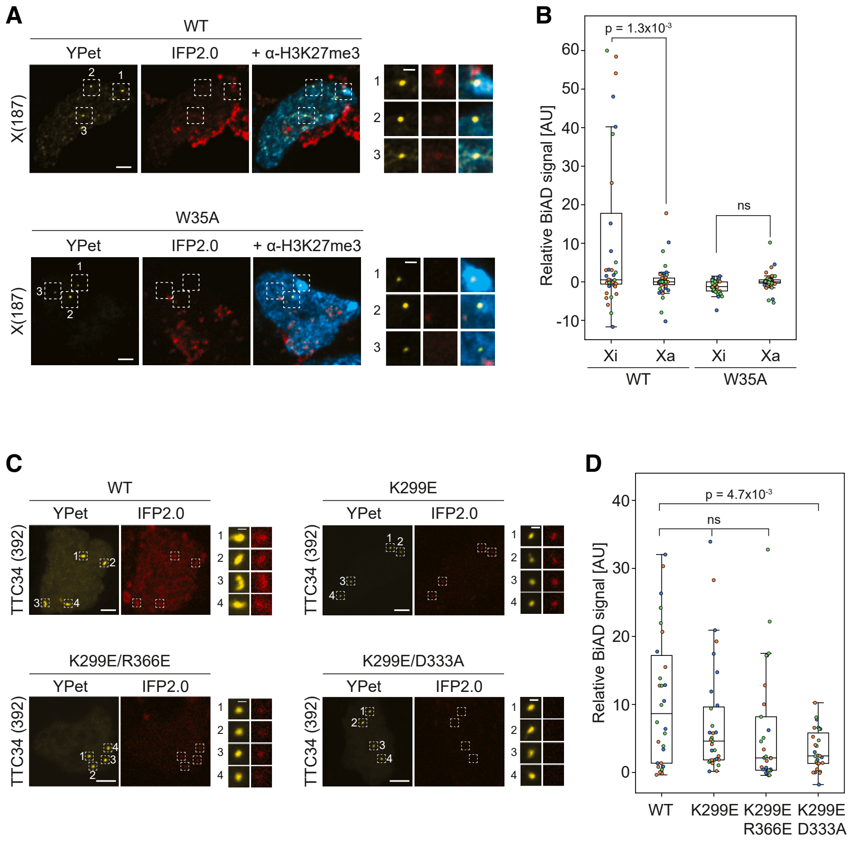

Figure 4. Validation of dual-color BiAD sensors for H3K27me3 and H3K36me2/3 readout

(A and B) HEK293 cells were transfected with all components of the dual-color BiAD sensor for H3K27me3 detection at the X(187) locus using either the WT 2×CBX7CD detector or a binding-deficient W35A mutant. Cells were immunostained for H3K27me3 to identify the inactive (Xi) and active (Xa) X chromosomes. (A) Representative fluorescence microscopy images of fixed cells showing the co-localization of the marker (YPet) and BiAD signals (IFP2.0) for the WT detector at the Xi (spot 1), which was not observed at the Xa (spots 2 and 3), or for transfections with the W35A mutant. The H3K27me3 immunostaining signal is shown in cyan. Scale bars, 5 μm and 1 μm for the magnified images. (B) Boxplots showing the BiAD signal (IFP2.0) relative to the marker signal (YPet). Individual spots were separated into Xi and Xa and normalized to the mean Xa signal for each cell. Data were obtained in 3 independent experiments (depicted in orange, blue, and green). Significance was determined via a 2-tailed, unpaired t test. The p values are indicated in the boxplot; ns, nonsignificant p values ($p > 0.05$).

(C and D) HEK293 cells were transfected with all of the components of the dual-color BiAD sensor for H3K36me2/3 detection at the TTC34 locus with either the WT 2×3APWWP detector or K299E, K299E/R366A, and K299E/D333A binding pocket mutants. (C) Exemplary fluorescence microscopy images of fixed cells showing the colocalization of the marker (YPet) and BiAD signal (IFP2.0) for the WT detector as well as the K299E and K299E/R366A mutants. No colocalization was observed for the K299E/D333A mutant, indicating that it is a suitable negative control. Scale bars, 5 μm and 1 μm for the magnified images. (D) Boxplots showing the relative BiAD intensities. Each dot represents the mean relative BiAD signal of all of the spots within a single cell. Data were obtained in 3 independent experiments (depicted in orange, blue, and green). Significance was determined via a 1-way ANOVA with Tukey's posttest. The p values are indicated in the boxplot; ns, nonsignificant p values ($p > 0.05$).

BiAD sensor could detect the enrichment of H3K9me2/3 on the Xi that had been reported previously.[39–41] Comparing the relative BiAD signals from cells transfected with 2×HP1βCD WT and W42A detector modules, we successfully identified H3K9me2/3 at the X(187) region in live cells (Figures 6A and S4A). To investigate whether our H3K9me2/3 BiAD sensor could detect allelic differences in H3K9me2/3 levels between Xi and Xa, we repeated the experiment in fixed cells and additionally stained the cells for H3K27me3 to identify the Xi (Figure 6B). When separating the individual spots into Xa and Xi, we found that the BiAD sensor detected more H3K9me2/3 at the Xi compared to the Xa (Figures 6B and 6C). Hence, the allelic difference in H3K9me2/3 levels at the X(187) locus on the X chromosomes could be detected by the H3K9me2/3 dual-color BiAD sensor.

Functional studies of H3K9me3 establishment on Xi using the dual-color BiAD sensor

Finally, we wanted to use our dual-color H3K9me2/3 BiAD sensor to examine the role of different H3K9 protein lysine methyltransferases (PKMTs) in the deposition and maintenance of H3K9me2/3 at the X(187) locus. For this, we used individual small interfering RNA (siRNA) knockdown of the five main human H3K9 PKMTs (SUV39H1, SUV39H2, SETDB1, G9a [EHMT2], and GLP [EHMT1]), followed by transfection of the dual-color BiAD sensor for H3K9me2/3 readout and analysis 5 days after knockdown of the PKMTs (Figure 6D). The efficient individual knockdown of all five PKMTs was confirmed by reverse transcription-qPCR (Figure S4B). Analysis of the BiAD signals comparing the detector WT to the binding-deficient W42A mutant for each knockdown revealed that cells transfected with control siRNA (siNT) as well as after SUV39H1, SUV39H2, and SETDB1 knockdown maintained H3K9me2/3 at the X(187) locus (Figures 6E, S4C, and S4D). In contrast, cells transfected with siRNAs depleting either G9a or GLP showed a markedly reduced BiAD signal at the X(187) locus, with no significant difference in relative BiAD signals obtained from cells transfected with WT or binding pocket mutant detector. These results indicate that both of these enzymes are crucial for H3K9me2 deposition at the X(187) locus, which is in agreement with the finding that they predominantly exist in a heteromeric complex.[42] In conclusion, our results highlight that dual-color BiAD sensors are valuable tools to gain insights into locus-specific and even allelic chromatin states and the enzymes involved in the regulation of the epigenome network.

DISCUSSION

Gaining insights into the locus-specific dynamics of epigenome modifications is essential to uncover the mechanisms occurring

CellPress
OPEN ACCESS

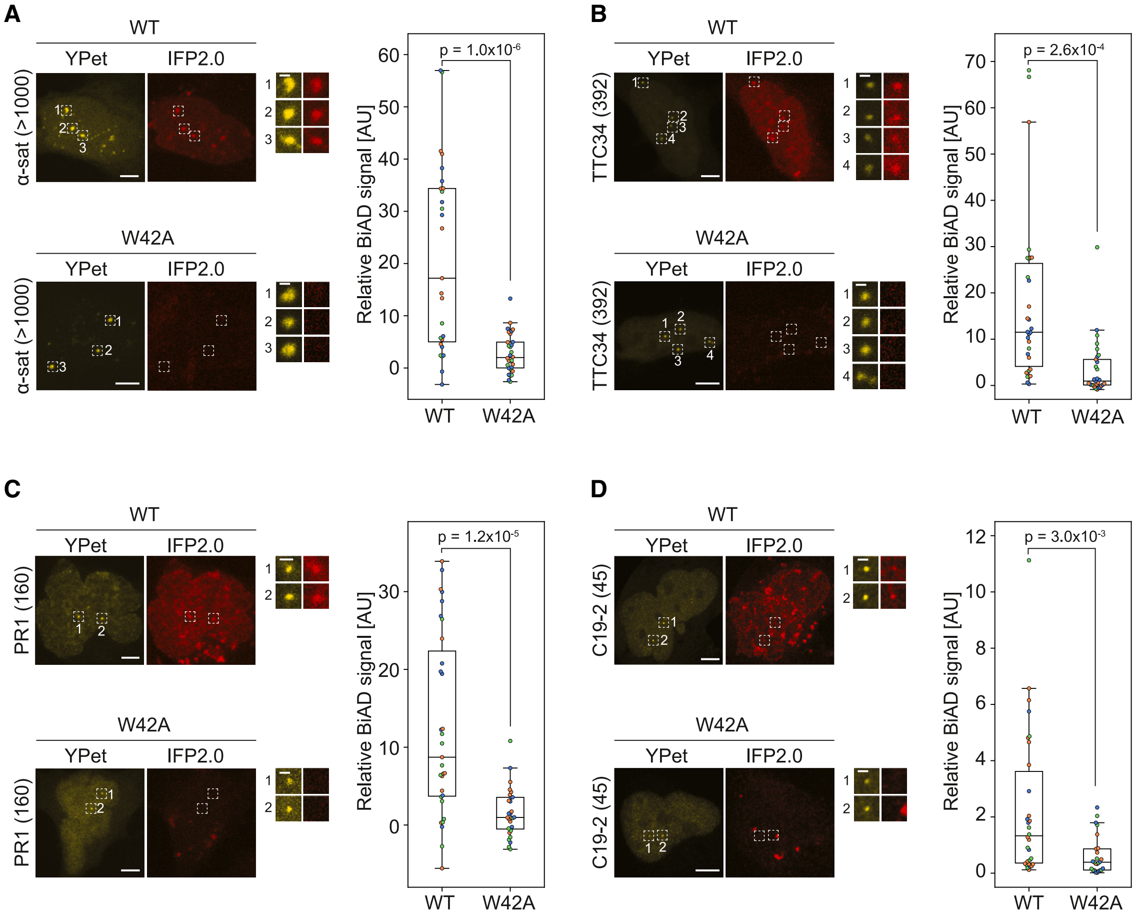

Figure 5. Dual-color BiAD sensor for H3K9me2/3 readout

HEK293 cells were transfected with all components of the dual-color BiAD sensor for H3K9me2/3 detection using either the WT 2×HP1βCD detector or a binding-deficient mutant (W42A) as negative control. The BiAD sensor was tested on different target loci with decreasing local repeat numbers: (A) α-satellites (>1,000 repeats), (B) TTC34 (392 repeats), (C) PR1 (160 repeats), and (D) C19-2 (45 repeats). In each panel, exemplary fluorescence microscopy images of fixed cells show the colocalization of the marker (YPet) and BiAD signal (IFP2.0) for the WT detector, but not for the binding-deficient R44Q mutant. Scale bars, 5 μm and 1 μm for the magnified images. Boxplots show the relative BiAD signals. Each dot represents the mean relative BiAD signal of all of the spots within a single cell. Data were obtained in 3 independent experiments (depicted in orange, blue, and green). Significance was determined via a 2-tailed, unpaired t test. The p values are indicated in each boxplot.

during cellular differentiation and development of diseases. To study these locus-specific changes at the single-cell level, we developed modular dual-color BiAD sensors that are compatible with live-cell imaging. By combining the fluorescent labeling of an endogenous target locus with the BiFC-based readout of chromatin modifications, we could, for the first time, detect DNA methylation as well as the histone PTMs H3K9me2/3, H3K27me3, and H3K36me2/3 at low copy repeat regions in single human cells. With this improved dual-color BiAD sensor design, the sensor sensitivity was improved strongly, from the detection limit at highly repetitive loci (>1,000 repeats) in the previously available BiAD sensors[17] to regions with as few as 45 local repeats. Furthermore, we demonstrated that the dual-color BiAD sensors allowed us to observe changes in DNA methylation upon DNMT1i treatment, indicating that it can be used to monitor dynamic responses to drug treatments in cells with locus resolution. Hence, the dual-color BiAD sensors could be applied as a tool for the development and validation of new (epi)drugs.

The dual-color design combining the YPet fluorophore marking the target locus with the split IFP2.0 fluorophore for BiFC readout of the chromatin modification was one critical step to achieve the improved sensitivity of the BiAD sensors. The YPet marker allows the tracking of the target locus in real time during live-cell imaging and facilitates the discrimination of the IFP2.0 BiAD signal at the locus of interest from unspecific nuclear background, enabling the quantitative and qualitative readout of epigenome modifications. The split site in the IFP2.0 fluorophore had been designed for reversible BiFC assays,[25] which could help to minimize the influence of the BiAD sensors on the three-dimensional (3D) chromatin structure caused by the enforced interaction of anchor and detector binding sites. It had been demonstrated in previous studies that multivalent interactions with the histone PTM increase the affinity of natural

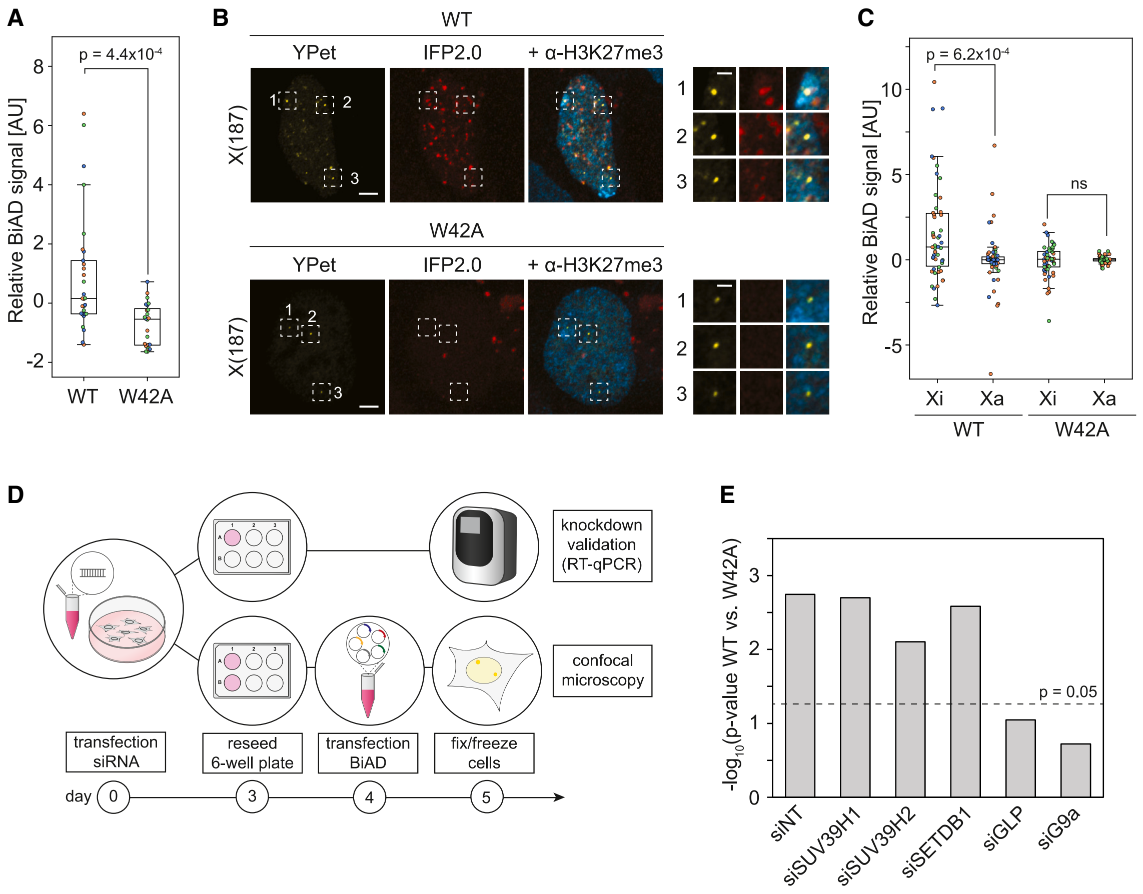

Figure 6. Dual-color BiAD sensor for H3K9me3 readout at the X chromosome

(A–C) HEK293 cells were transfected with all components of the dual-color BiAD sensor for H3K9me2/3 detection at the X(187) locus using either the WT 2× HP1βCD detector or a binding-deficient mutant (W42A) as negative control. (A) Boxplots showing the relative BiAD signal in live cells (exemplary images are shown in Figure S4A). Each dot represents the mean relative BiAD signal of all of the spots within a single cell. (B) Fixed cells were immunostained for H3K27me3 (shown in cyan) to identify the inactive (Xi) X chromosomes. Exemplary fluorescence microscopy images showing the colocalization of the marker (YPet) and BiAD signal (IFP2.0) for the WT detector at the Xi (spot 1), less BiAD signal at the Xa (spots 2 and 3), and no colocalization for transfections with the W42A mutant. Scale bars, 5 μm and 1 μm for the magnified images. (C) Individual spots were separated into Xi and Xa and normalized to the mean Xa signal for each cell. The p values are indicated in the boxplots in (B) and (C); ns, nonsignificant p value ($p > 0.05$).

(D and E) HEK293 cells were transfected with siRNAs targeting the human H3K9 methyltransferases SUV39H1, SUV39H2, SETDB1, GLP, and G9a or a control siRNA (siNT) and subsequently reseeded and transfected with the dual-color BiAD sensor for H3K9me2/3 detection at the X(187) locus. (D) Schematic representation of the experimental setup. (E) p values derived from the quantitative analysis (Figure S4C) of fluorescence microscopy images comparing relative BiAD signals of cells transfected with 2×HP1βCD WT and W42A detector modules (exemplary images are shown in Figure S4D).

reader domains for their respective mark.[29,31–33] Exploiting this principle, we showed that the use of double reader domains enabled the locus-specific detection of the histone PTMs H3K9me2/3, H3K36me2/3, and H3K27me3 in human cells with dual-color BiAD sensors.

It has been shown previously that H3K27me3 and H3K9me2/3 are enriched on the inactive X chromosome.[39–41] Using our dual-color BiAD sensors with additional double domain detector modules for H3K27me3 and H3K9me2/3 readout, we were able to detect the allelic enrichment of these marks at the X(187) locus on the inactive X chromosome. Furthermore, the dual-color BiAD sensors were used successfully to dissect the H3K9 PKMT regulatory network at the X chromosome. Using individual knockdowns of the five main human H3K9 PKMTs, G9a, GLP, SUV39H1, SUV39H2, and SETDB1, we identified that G9a and GLP, which are known to form heteromeric complexes,[42] are mainly responsible for H3K9me2 deposition at the X(187) locus. This result fits to previous data because G9a/GLP have been shown to interact with multiple X chromosome-associated factors such as the long noncoding RNAs Xist and Tsix,[43] as well as polycomb repressive complex 2, responsible for H3K27me3 deposition at the inactive X chromosome,[44] and the loss of G9a causes failure of X chromosome inactivation.[43] These findings highlight the unique potential of dual-color BiAD sensors to investigate the locus-specific changes of epigenome modifications in live cells. A recent study used fluorescently labeled mintbodies to visualize the accumulation of H3K27me3 and H4K20me1 during X chromosome inactivation.[45] Although this method allowed for tracking the Xi and Xa in live cells using dCas9-EGFP targeted to microsatellite repeats on the X

chromosome, it lacked locus-specific resolution of the analysis of the epigenome marks. Using our dual-color BiAD sensor for locus-specific H3K27me3 readout, an additional dimension could be added by analyzing the H3K27me3 dynamics at different dCas9 target loci on the X chromosome to further dissect the spatiotemporal dynamics during the establishment of X chromosome inactivation.

In our experimental setup using the HEK293 cell line, we encountered limitations with the Sirius-MS2 system to visualize regions containing fewer than 45 local repeats. However, even with this current limitation, 254 or 266 endogenous loci in the human reference genomes of female and male cells, respectively, are already within the detection range of our dual-color BiAD sensors (http://genome.ucf.edu/CRISPRbar/).[19,20] Among these potential target loci, which, for example, include the TERT gene, 143 (XX)/146 (XY) are either in gene bodies or gene control regions, illustrating potential applications of our dual color BiAD sensors with direct biological significance (Table S1). To explore the full potential of the dual-color BiAD sensors to detect chromatin modifications at even lower copy loci and ultimately with single-locus resolution, it will be essential to enhance the detection limit of the marker fluorophore. This could be achieved by a different sgRNA-based recruitment mechanism, such as the recently published Casilio system for single-locus detection.[22] Alternative approaches could include tiling of the dual-color BiAD sensors using multiple individual sgRNAs to visualize a nonrepetitive locus.[46–48] Moreover, further protein engineering of the reader domain as demonstrated for H3K9me2/3 and H3K27me3 readers[31,49] may lead to an increased sensitivity and specificity of the BiAD signal. Achieving single-locus resolution would be particularly valuable in the context of active modifications such as H3K4 methylation or histone acetylation, which mostly occur at nonrepetitive sites. In this context, the double domain approach will be applicable because the improved functionality of corresponding double reader domains has already been demonstrated such as the TAF3 PHD domain for H3K4me3[29] as well as the second bromodomain of human polybromodomain 1 for H3K14ac readout.[33]

In summary, the dual-color BiAD sensors developed in this study provide a powerful experimental approach for detecting chromatin PTMs at genomic loci containing lower copy repeats and ultimately at single-locus resolution. These sensors offer a modular and versatile toolbox that is compatible with live-cell studies, enabling the visualization of dynamic changes in epigenomic marks at endogenous target loci in single cells and allowing a combined analysis of cellular phenotypes and epigenome modifications. In combination with a broad palette of detector modules for readout of various chromatin PTMs, the dual-color BiAD sensors will greatly enhance our understanding of locus-specific dynamic epigenome regulation processes at the single-cell level. From the long-term perspective, it would be fascinating to express BiAD sensors in animals and follow epigenetic reprogramming of developing tissues with time and cellular resolution in living animals.

Limitations of the study

As discussed above, the main limitation of the current dual-color BiAD sensors is their restriction to target sites containing $\geq$45 local repeats. This limitation may be overcome with further optimization, new fluorophores, or improved imaging technology. Moreover, as with any CRISPR-dCas9 methodology, it cannot be excluded that the binding of sgRNA/dCas9 complexes affects chromatin modifications, its 3D structure, and/or binding of endogenous chromatin-binding factors in the timescale relevant to our experiments. On the technical side, one limitation of our study is that in the current design of the sensors, cotransfection of several plasmids is necessary and, therefore, each sample of transfected cells contained cells that did not contain all of the required BiAD parts and hence remained BiAD negative. This problem could be circumvented in future by the generation of stable cell lines expressing some of the BiAD components or by combining multiple BiAD components on the same plasmid. Another technical limitation is that currently the thresholds to define ROIs were manually defined for each nucleus depending on its fluorescence intensity. This setting may be automated in improved versions of the workflow.

STAR★METHODS

Detailed methods are provided in the online version of this paper and include the following:

- KEY RESOURCES TABLE
- RESOURCE AVAILABILITY
  - Lead contact
  - Materials availability
  - Data and code availability
- EXPERIMENTAL MODEL AND STUDY PARTICIPANT DETAILS
  - Cell culture
- METHOD DETAILS
  - Retrieval of repetitive genomic loci and cloning of sgRNA and dCas9-SunTag vectors
  - Cloning of detector domains
  - Transfections
  - Fixation and antibody staining
  - Fluorescence microscopy and image analysis
  - siRNA knockdown of H3K9 methyltransferases
  - DNMT1 inhibitor treatment
- QUANTIFICATION AND STATISTICAL ANALYSIS

SUPPLEMENTAL INFORMATION

Supplemental information can be found online at https://doi.org/10.1016/j.crmeth.2024.100739.

ACKNOWLEDGMENTS

The authors gratefully acknowledge the Technology Platform "Cellular Analytics" of the Stuttgart Research Center Systems Biology for their support and assistance in this work. This work has been supported by the Baden-Württemberg Stiftung gGmbH (EpiSensor, ID07, to A.J. and M.A.O.). The funder had no role in the design of the study and collection, analysis, and interpretation of data or in the writing of the manuscript.

AUTHOR CONTRIBUTIONS

A.R.K., P.B., and A.J. devised the study with contributions from C.L. and M.A.O. A.R.K. conducted all of the experiments with help from J.H., A.H.,

S.B., and L.H. A.R.K., P.B., and A.J. were involved in the data analysis. P.B. and A.J. supervised the work. L.-M.B. and C.L. provided resources. A.R.K. and A.J. wrote the draft article and prepared the figures. A.J. and M.A.O. acquired the funding. All of the authors were involved in preparing the final manuscript.

DECLARATION OF INTERESTS

The authors declare no competing interests.

Received: October 10, 2023
Revised: February 1, 2024
Accepted: February 28, 2024
Published: March 29, 2024

REFERENCES

1. Allis, C.D., and Jenuwein, T. (2016). The molecular hallmarks of epigenetic control. Nat. Rev. Genet. *17*, 487–500. https://doi.org/10.1038/nrg.2016.59.
2. Tucci, V., Isles, A.R., Kelsey, G., and Ferguson-Smith, A.C.; Erice Imprinting Group (2019). Genomic Imprinting and Physiological Processes in Mammals. Cell *176*, 952–965. https://doi.org/10.1016/j.cell.2019.01.043.
3. Żylicz, J.J., and Heard, E. (2020). Molecular Mechanisms of Facultative Heterochromatin Formation: An X-Chromosome Perspective. Annu. Rev. Biochem. *89*, 255–282. https://doi.org/10.1146/annurev-biochem-062917-012655.
4. Luo, C., Hajkova, P., and Ecker, J.R. (2018). Dynamic DNA methylation: In the right place at the right time. Science *361*, 1336–1340. https://doi.org/10.1126/science.aat6806.
5. Chen, Z., and Zhang, Y. (2020). Role of Mammalian DNA Methyltransferases in Development. Annu. Rev. Biochem. *89*, 135–158. https://doi.org/10.1146/annurev-biochem-103019-102815.
6. Jambhekar, A., Dhall, A., and Shi, Y. (2019). Roles and regulation of histone methylation in animal development. Nat. Rev. Mol. Cell Biol. *20*, 625–641. https://doi.org/10.1038/s41580-019-0151-1.
7. Mazzone, R., Zwergel, C., Artico, M., Taurone, S., Ralli, M., Greco, A., and Mai, A. (2019). The emerging role of epigenetics in human autoimmune disorders. Clin. Epigenet. *11*, 34. https://doi.org/10.1186/s13148-019-0632-2.
8. Nestler, E.J., Peña, C.J., Kundakovic, M., Mitchell, A., and Akbarian, S. (2016). Epigenetic Basis of Mental Illness. Neuroscientist *22*, 447–463. https://doi.org/10.1177/1073858415608147.
9. Ling, C., Bacos, K., and Rönn, T. (2022). Epigenetics of type 2 diabetes mellitus and weight change - a tool for precision medicine? Nat. Rev. Endocrinol. *18*, 433–448. https://doi.org/10.1038/s41574-022-00671-w.
10. Hanahan, D. (2022). Hallmarks of Cancer: New Dimensions. Cancer Discov. *12*, 31–46. https://doi.org/10.1158/2159-8290.Cd-21-1059.
11. Gupta, R.K., and Kuznicki, J. (2020). Biological and Medical Importance of Cellular Heterogeneity Deciphered by Single-Cell RNA Sequencing. Cells *9*. https://doi.org/10.3390/cells9081751.
12. Dagogo-Jack, I., and Shaw, A.T. (2018). Tumour heterogeneity and resistance to cancer therapies. Nat. Rev. Clin. Oncol. *15*, 81–94. https://doi.org/10.1038/nrclinonc.2017.166.
13. Casado-Pelaez, M., Bueno-Costa, A., and Esteller, M. (2022). Single cell cancer epigenetics. Trends Cancer *8*, 820–838. https://doi.org/10.1016/j.trecan.2022.06.005.
14. LaFave, L.M., Savage, R.E., and Buenrostro, J.D. (2022). Single-Cell Epigenomics Reveals Mechanisms of Cancer Progression. Annu. Rev. Cell Biol. *6*, 167–185.
15. Clark, S.J., Lee, H.J., Smallwood, S.A., Kelsey, G., and Reik, W. (2016). Single-cell epigenomics: powerful new methods for understanding gene regulation and cell identity. Genome Biol. *17*, 72. https://doi.org/10.1186/s13059-016-0944-x.
16. Mazan-Mamczarz, K., Ha, J., De, S., and Sen, P. (2022). Single-Cell Analysis of the Transcriptome and Epigenome. Methods Mol. Biol. *2399*, 21–60. https://doi.org/10.1007/978-1-0716-1831-8_3.
17. Lungu, C., Pinter, S., Broche, J., Rathert, P., and Jeltsch, A. (2017). Modular fluorescence complementation sensors for live cell detection of epigenetic signals at endogenous genomic sites. Nat. Commun. *8*, 649–713. https://doi.org/10.1038/s41467-017-00457-z.
18. Tanenbaum, M.E., Gilbert, L.A., Qi, L.S., Weissman, J.S., and Vale, R.D. (2014). A protein-tagging system for signal amplification in gene expression and fluorescence imaging. Cell *159*, 635–646. https://doi.org/10.1016/j.cell.2014.09.039.
19. Ma, H., Tu, L.-C., Naseri, A., Huisman, M., Zhang, S., Grunwald, D., and Pederson, T. (2016). Multiplexed labeling of genomic loci with dCas9 and engineered sgRNAs using CRISPRainbow. Nat. Biotechnol. *34*, 528–530. https://doi.org/10.1038/nbt.3526.
20. Ma, H., Tu, L.-C., Naseri, A., Chung, Y.-C., Grunwald, D., Zhang, S., and Pederson, T. (2018). CRISPR-Sirius: RNA scaffolds for signal amplification in genome imaging. Nat. Methods *15*, 928–931. https://doi.org/10.1038/s41592-018-0174-0.
21. Cheng, A.W., Jillette, N., Lee, P., Plaskon, D., Fujiwara, Y., Wang, W., Taghbalout, A., and Wang, H. (2016). Casilio: a versatile CRISPR-Cas9-Pumilio hybrid for gene regulation and genomic labeling. Cell Res. *26*, 254–257. https://doi.org/10.1038/cr.2016.3.
22. Clow, P.A., Du, M., Jillette, N., Taghbalout, A., Zhu, J.J., and Cheng, A.W. (2022). CRISPR-mediated multiplexed live cell imaging of nonrepetitive genomic loci with one guide RNA per locus. Nat. Commun. *13*, 1871. https://doi.org/10.1038/s41467-022-29343-z.
23. Nguyen, A.W., and Daugherty, P.S. (2005). Evolutionary optimization of fluorescent proteins for intracellular FRET. Nat. Biotechnol. *23*, 355–360. https://doi.org/10.1038/nbt1066.
24. Lin, Y.C., Boone, M., Meuris, L., Lemmens, I., Van Roy, N., Soete, A., Reumers, J., Moisse, M., Plaisance, S., Drmanac, R., et al. (2014). Genome dynamics of the human embryonic kidney 293 lineage in response to cell biology manipulations. Nat. Commun. *5*, 4767. https://doi.org/10.1038/ncomms5767.
25. Tchekanda, E., Sivanesan, D., and Michnick, S.W. (2014). An infrared reporter to detect spatiotemporal dynamics of protein-protein interactions. Nat. Methods *11*, 641–644. https://doi.org/10.1038/nmeth.2934.
26. Chen, M., Yan, C., Ma, Y., and Zhang, X.-E. (2021). A tandem near-infrared fluorescence complementation system with enhanced fluorescence for imaging protein-protein interactions in vivo. Biomaterials *268*, 120544. https://doi.org/10.1016/j.biomaterials.2020.120544.
27. Ohki, I., Shimotake, N., Fujita, N., Jee, J., Ikegami, T., Nakao, M., and Shirakawa, M. (2001). Solution structure of the methyl-CpG binding domain of human MBD1 in complex with methylated DNA. Cell *105*, 487–497. https://doi.org/10.1016/s0092-8674(01)00324-5.
28. Pappalardi, M.B., Keenan, K., Cockerill, M., Kellner, W.A., Stowell, A., Sherk, C., Wong, K., Pathuri, S., Briand, J., Steidel, M., et al. (2021). Discovery of a first-in-class reversible DNMT1-selective inhibitor with improved tolerability and efficacy in acute myeloid leukemia. Nat. Can. (Ott.) *2*, 1002–1017.
29. Villaseñor, R., Pfaendler, R., Ambrosi, C., Butz, S., Giuliani, S., Bryan, E., Sheahan, T.W., Gable, A.L., Schmolka, N., Manzo, M., et al. (2020). ChromID identifies the protein interactome at chromatin marks. Nat. Biotechnol. *38*, 728–736. https://doi.org/10.1038/s41587-020-0434-2.
30. Mauser, R., Kungulovski, G., Keup, C., Reinhardt, R., and Jeltsch, A. (2017). Application of dual reading domains as novel reagents in chromatin biology reveals a new H3K9me3 and H3K36me2/3 bivalent chromatin state. Epigenet. Chromatin *10*, 45. https://doi.org/10.1186/s13072-017-0153-1.
31. Veggiani, G., Villaseñor, R., Martyn, G.D., Tang, J.Q., Krone, M.W., Gu, J., Chen, C., Waters, M.L., Pearce, K.H., Baubec, T., and Sidhu, S.S. (2022). High-affinity chromodomains engineered for improved detection of

CellPress
OPEN ACCESS

histone methylation and enhanced CRISPR-based gene repression. Nat. Commun. *13*, 6975. https://doi.org/10.1038/s41467-022-34269-7.

32. Tekel, S.J., Vargas, D.A., Song, L., LaBaer, J., Caplan, M.R., and Haynes, K.A. (2018). Tandem Histone-Binding Domains Enhance the Activity of a Synthetic Chromatin Effector. ACS Synth. Biol. *7*, 842–852. https://doi.org/10.1021/acssynbio.7b00281.
33. Sanchez, O.F., Mendonca, A., Carneiro, A.D., and Yuan, C. (2017). Engineering Recombinant Protein Sensors for Quantifying Histone Acetylation. ACS Sens. *2*, 426–435. https://doi.org/10.1021/acssensors.7b00026.
34. Dukatz, M., Holzer, K., Choudalakis, M., Emperle, M., Lungu, C., Bashtrykov, P., and Jeltsch, A. (2019). H3K36me2/3 Binding and DNA Binding of the DNA Methyltransferase DNMT3A PWWP Domain Both Contribute to its Chromatin Interaction. J. Mol. Biol. *431*, 5063–5074. https://doi.org/10.1016/j.jmb.2019.09.006.
35. Kungulovski, G., Kycia, I., Tamas, R., Jurkowska, R.Z., Kudithipudi, S., Henry, C., Reinhardt, R., Labhart, P., and Jeltsch, A. (2014). Application of histone modification-specific interaction domains as an alternative to antibodies. Genome Res. *24*, 1842–1853. https://doi.org/10.1101/gr.170985.113.
36. Kaustov, L., Ouyang, H., Amaya, M., Lemak, A., Nady, N., Duan, S., Wasney, G.A., Li, Z., Vedadi, M., Schapira, M., et al. (2011). Recognition and specificity determinants of the human cbx chromodomains. J. Biol. Chem. *286*, 521–529. https://doi.org/10.1074/jbc.M110.191411.
37. Rougeulle, C., Chaumeil, J., Sarma, K., Allis, C.D., Reinberg, D., Avner, P., and Heard, E. (2004). Differential Histone H3 Lys-9 and Lys-27 Methylation Profiles on the X Chromosome. Mol. Cell Biol. *24*, 5475–5484. https://doi.org/10.1128/mcb.24.12.5475-5484.2004.
38. Li, J., Ahn, J.H., and Wang, G.G. (2019). Understanding histone H3 lysine 36 methylation and its deregulation in disease. Cell. Mol. Life Sci. *76*, 2899–2916. https://doi.org/10.1007/s00018-019-03144-y.
39. Brinkman, A.B., Roelofsen, T., Pennings, S.W.C., Martens, J.H.A., Jenuwein, T., and Stunnenberg, H.G. (2006). Histone modification patterns associated with the human X chromosome. EMBO Rep. *7*, 628–634. https://doi.org/10.1038/sj.embor.7400686.
40. Bonora, G., and Disteche, C.M. (2017). Structural aspects of the inactive X chromosome. Philos. Trans. R. Soc. Lond. B Biol. Sci. *372*, 20160357. https://doi.org/10.1098/rstb.2016.0357.
41. Vallot, C., Ouimette, J.-F., and Rougeulle, C. (2016). Establishment of X chromosome inactivation and epigenomic features of the inactive X depend on cellular contexts. Bioessays *38*, 869–880. https://doi.org/10.1002/bies.201600121.
42. Tachibana, M., Ueda, J., Fukuda, M., Takeda, N., Ohta, T., Iwanari, H., Sakihama, T., Kodama, T., Hamakubo, T., and Shinkai, Y. (2005). Histone methyltransferases G9a and GLP form heteromeric complexes and are both crucial for methylation of euchromatin at H3-K9. Genes Dev. *19*, 815–826. https://doi.org/10.1101/gad.1284005.
43. Szanto, A., Aguilar, R., Kesner, B., Blum, R., Wang, D., Cifuentes-Rojas, C., Del Rosario, B.C., Kis-Toth, K., and Lee, J.T. (2021). A disproportionate impact of G9a methyltransferase deficiency on the X chromosome. Genes Dev. *35*, 1035–1054. https://doi.org/10.1101/gad.337592.120.
44. Mozzetta, C., Pontis, J., Fritsch, L., Robin, P., Portoso, M., Proux, C., Margueron, R., and Ait-Si-Ali, S. (2014). The histone H3 lysine 9 methyltransferases G9a and GLP regulate polycomb repressive complex 2-mediated gene silencing. Mol. Cell *53*, 277–289. https://doi.org/10.1016/j.molcel.2013.12.005.
45. Tjalsma, S.J.D., Hori, M., Sato, Y., Bousard, A., Ohi, A., Raposo, A.C., Roensch, J., Le Saux, A., Nogami, J., Maehara, K., et al. (2021). H4K20me1 and H3K27me3 are concurrently loaded onto the inactive X chromosome but dispensable for inducing gene silencing. EMBO Rep. *22*, e51989. https://doi.org/10.15252/embr.202051989.
46. Chen, B., Gilbert, L.A., Cimini, B.A., Schnitzbauer, J., Zhang, W., Li, G.-W., Park, J., Blackburn, E.H., Weissman, J.S., Qi, L.S., and Huang, B. (2013). Dynamic Imaging of Genomic Loci in Living Human Cells by an Optimized CRISPR/Cas System. Cell *155*, 1479–1491. https://doi.org/10.1016/j.cell.2013.12.001.
47. Qin, P., Parlak, M., Kuscu, C., Bandaria, J., Mir, M., Szlachta, K., Singh, R., Darzacq, X., Yildiz, A., and Adli, M. (2017). Live cell imaging of low- and non-repetitive chromosome loci using CRISPR-Cas9. Nat. Commun. *8*, 14725. https://doi.org/10.1038/ncomms14725.
48. Gu, B., Swigut, T., Spencley, A., Bauer, M.R., Chung, M., Meyer, T., and Wysocka, J. (2018). Transcription-coupled changes in nuclear mobility of mammalian cis-regulatory elements. Science (New York, N.Y.) *359*, 1050–1055. https://doi.org/10.1126/science.aao3136.
49. Albanese, K.I., Krone, M.W., Petell, C.J., Parker, M.M., Strahl, B.D., Brustad, E.M., and Waters, M.L. (2020). Engineered Reader Proteins for Enhanced Detection of Methylated Lysine on Histones. ACS Chem. Biol. *15*, 103–111. https://doi.org/10.1021/acschembio.9b00651.
50. Schindelin, J., Arganda-Carreras, I., Frise, E., Kaynig, V., Longair, M., Pietzsch, T., Preibisch, S., Rueden, C., Saalfeld, S., Schmid, B., et al. (2012). Fiji: an open-source platform for biological-image analysis. Nat. Methods *9*, 676–682. https://doi.org/10.1038/nmeth.2019.
51. Hunter, J.D. (2007). Matplotlib: A 2D Graphics Environment. Comput. Sci. Eng. *9*, 90–95. https://doi.org/10.1109/mcse.2007.55.
52. Waskom, M. (2021). seaborn: statistical data visualization. J. Open Source Softw. *6*, 3021. https://doi.org/10.21105/joss.03021.
53. Pisal, R.V., Hrebíková, H., Chvátalová, J., Kunke, D., Filip, S., and Mokrý, J. (2016). Detection of Mycoplasma Contamination Directly from Culture Supernatant Using Polymerase Chain Reaction. Folia Biol. *62*, 203–206.
54. Ma, H., Naseri, A., Reyes-Gutierrez, P., Wolfe, S.A., Zhang, S., and Pederson, T. (2015). Multicolor CRISPR labeling of chromosomal loci in human cells. Proc. Natl. Acad. Sci. USA *112*, 3002–3007. https://doi.org/10.1073/pnas.1420024112.
55. Yu, D., Gustafson, W.C., Han, C., Lafaye, C., Noirclerc-Savoye, M., Ge, W.-P., Thayer, D.A., Huang, H., Kornberg, T.B., Royant, A., et al. (2014). An improved monomeric infrared fluorescent protein for neuronal and tumour brain imaging. Nat. Commun. *5*, 3626–3627. https://doi.org/10.1038/ncomms4626.
56. Mehta, S., Aye-Han, N.-N., Ganesan, A., Oldach, L., Gorshkov, K., and Zhang, J. (2014). Calmodulin-controlled spatial decoding of oscillatory Ca2+ signals by calcineurin. Elife *3*, e03765. https://doi.org/10.7554/eLife.03765.
57. Morita, S., Noguchi, H., Horii, T., Nakabayashi, K., Kimura, M., Okamura, K., Sakai, A., Nakashima, H., Hata, K., Nakashima, K., and Hatada, I. (2016). Targeted DNA demethylation in vivo using dCas9-peptide repeat and scFv-TET1 catalytic domain fusions. Nat. Biotechnol. *34*, 1060–1065. https://doi.org/10.1038/nbt.3658.
58. Koushik, S.V., Chen, H., Thaler, C., Puhl, H.L., 3rd, and Vogel, S.S. (2006). Cerulean, Venus, and VenusY67C FRET reference standards. Biophys. J. *91*, L99–L101. https://doi.org/10.1529/biophysj.106.096206.
59. Jeltsch, A., and Lanio, T. (2002). Site-directed mutagenesis by polymerase chain reaction. Methods Mol. Biol. *182*, 85–94. https://doi.org/10.1385/1-59259-194-9:085.
60. Bock, I., Kudithipudi, S., Tamas, R., Kungulovski, G., Dhayalan, A., and Jeltsch, A. (2011). Application of Celluspots peptide arrays for the analysis of the binding specificity of epigenetic reading domains to modified histone tails. BMC Biochem. *12*, 48. https://doi.org/10.1186/1471-2091-12-48.
61. Broche, J., Kungulovski, G., Bashtrykov, P., Rathert, P., and Jeltsch, A. (2021). Genome-wide investigation of the dynamic changes of epigenome modifications after global DNA methylation editing. Nucleic Acids Res. *49*, 158–176. https://doi.org/10.1093/nar/gkaa1169.

STAR★METHODS

KEY RESOURCES TABLE

REAGENT or RESOURCE	SOURCE	IDENTIFIER
**Antibodies**		
α-H3K36me3	Abcam	ab9050
α-H3K27me3	Active Motif	39155
α-H3K27me2/3	Active Motif	39536
α-H3K9me3	Diagenode	C15410193
goat α-rabbit IgG Alexa Fluor™ 405	Invitrogen	A-48258
goat α-mouse IgG Alexa Fluor™ 405	Invitrogen	A-31553
goat α-mouse IgG Alexa Fluor™ 594	Invitrogen	A-11005
goat α-mouse IgG Alexa Fluor™ 633	Invitrogen	A-21052
goat α-rabbit IgG Alexa Fluor™ 647	Invitrogen	A-32733
**Bacterial and virus strains**		
E. coli XL1 Blue	Agilent	200228
E. coli Stbl3™	Invitrogen	C737303
**Chemicals, peptides, and recombinant proteins**		
DNMT1 inhibitor GSK-3685032	MedChemExpress	HY-139664
Biliverdin	Sigma-Aldrich	30891
**Critical commercial assays**		
Celluspot™ arrays	Active Motif	Cat# 13001
**Deposited data**		
ImageJ macro for image analysis	This paper	https://doi.org/10.6084/m9.figshare.23592894
**Experimental models: Cell lines**		
HEK293	DSMZ	RRID: CVCL_0045
NIH3T3	ATCC	RRID: CRL-1658
**Oligonucleotides**		
Primers for siRNA knockdown validation, see Table S7	This paper	N/A
Primers for 5mC-sensitive qPCR, see Table S7	This paper	N/A
siRNAs used for H3K9 methyltransferase knockdown, see Table S5	This paper	N/A
**Recombinant DNA**		
Plasmids deposited with Addgene, see Table S7	This paper	N/A
**Software and algorithms**		
FIJI, ImageJ 1.54b	Schindelin et al.[50]	https://imagej.net/software/fiji/
Graphpad Prism 5	Graphpad Software, Inc	N/A
Illustrator CS6	Adobe	N/A
Python 3.9.7	Python Software Foundation	https://www.python.org/downloads/
Matplotlib	Hunter et al.[51]	N/A
Seaborn	Waskom et al.[52]	N/A
Excel 2016	Microsoft	N/A
ZEN 3.0 SR (black edition) and Zen 3.0 (blue edition)	Zeiss	N/A
**Other**		
sgRNA sequences, see Table S2	Ma et al.[20]	http://genome.ucf.edu/CRISPRbar/

RESOURCE AVAILABILITY

Lead contact

Further information and requests for resources should be directed to and will be fulfilled by the lead contact, Albert Jeltsch (albert.jeltsch@ibtb.uni-stuttgart.de).

Materials availability

Plasmids generated in this study have been deposited to Addgene (Table S2).

Data and code availability

- All data reported in this paper will be shared by the lead contact upon request.
- All original code used for image analysis has been deposited at figshare.com and is publicly available as of the date of publication. DOIs are listed in the key resources table.
- Any additional information needed to reanalyze the data reported in this paper is available from the lead contact upon request.

EXPERIMENTAL MODEL AND STUDY PARTICIPANT DETAILS

Cell culture

Human embryonic kidney (HEK293; female; RRID: CVCL_0045) and NIH3T3 (male; RRID: CRL-1658) cells were obtained from DSMZ (Braunschweig, Germany) and ATCC (American Type Culture Collection), respectively. HEK293 and NIH3T3 cells were cultivated in DMEM culture medium (DMEM supplemented with 10% Fetal Bovine Serum (FBS), 1% penicillin/streptomycin, 4 mM L-glutamine) in a humidified incubator (BINDER) at 37°C and 5% CO_2. To maintain a confluency of 70–90%, cells were split at a 1:7 to 1:10 ratio every 2 to 3 days. For this, the cells were washed with PBS (without $CaCl_2$ and $MgCl_2$), followed by addition of trypsin-EDTA solution (Sigma) and incubation at 37°C until cells detached. Afterward, cells were resuspended using culture medium and split in the desired ratio. For storage, cells were harvested at 300 g for 5 min, resuspended in freezing medium (90% FBS, 10% DMSO) and gradually frozen to −80°C. Cells were routinely checked for mycoplasma contamination by PCR.[53]

METHOD DETAILS

Retrieval of repetitive genomic loci and cloning of sgRNA and dCas9-SunTag vectors

Endogenous loci in the human genome containing repetitive sequences, which can be targeted by CRISPR-imaging, were retrieved from the CRISPRbar web server[54] (http://genome.ucf.edu/CRISPRbar/) using default settings. A subset of them containing gene-associated target loci within the detection range of the dual-color BiAD sensors is provided in Table S1. For cloning, the 12 nucleotides preceding the PAM sequence (NGG) were taken as sgRNA sequence. A list of all sgRNA sequences used in this study is provided in Table S3. The individual sgRNA sequences taken from[20,54] were inserted into pPUR-hU6-sgRNA-Sirius-8XMS2 using Golden Gate Assembly as described in.[54] pPUR-hU6-sgRNA-Sirius-8XMS2 was a gift from Thoru Pederson[20] (Addgene plasmid #121942; RRID:Addgene_121942).

To generate an expression construct for the GCN4-binding single-chain fragment variable (scFv) fused to the N-terminal part of mVenus or IFP2.0, the region encoding sfGFP in pHR-scFv-GCN4-sfGFP-GB1-NLS-dWPRE was replaced by the N-terminal part of split mVenus (amino acid 1–210, Addgene plasmid # 27794) or IFP2.0 (amino acid 1–132, Addgene plasmid # 54784) using the Gibson Assembly mix (New England Biolabs). pHR-scFv-GCN4-sfGFP-GB1-NLS-dWPRE was a gift from Ron Vale[18] (Addgene plasmid #60906; RRID:Addgene_60906). IFP2.0-C1 was a gift from Michael Davidson & Xiaokun Shu[55] (Addgene plasmid # 54784; RRID:Addgene_54784).

To obtain an expression construct for the MS2 coat protein (MCP) RNA-binding domain fused to YPet, the HaloTag in pHAGE-EFS-MCP-HALOnls was replaced with YPet from pcDNA3-Cyto-CaNAR2 using Gibson Assembly. The pHAGE-EFS-MCP-HALOnls plasmid was a gift from Thoru Pederson[20] (Addgene plasmid # 121937; RRID:Addgene_121937). The pcDNA3-Cyto-CaNAR2 plasmid containing the YPet fluorophore was a gift from Jin Zhang[56] (Addgene plasmid # 64729; RRID:Addgene_64729).

The dCas9-SunTag plasmid pCAG-dCas9-5xPlat2AflD was a gift from Izuho Hatada[57] (Addgene plasmid # 82560; RRID:Addgene_82560). This plasmid was used for DNA methylation detection. For detection of histone posttranslational modifications, five additional copies of the GCN4 epitope were inserted using the BamHI restriction site to generate a SunTag with ten GCN4 epitopes, separated by 22 amino acid linkers.

Cloning of detector domains

All chromatin reader domains were cloned as single or double domain into the mVenus-C1 mammalian expression vector from Steven Vogel[58] (Addgene plasmid # 27794; RRID:Addgene_27794) using the Gibson Assembly mix as described in.[17] For the double domain plasmids, the domains were separated by a 12 amino acid linker containing an SV40 large T-antigen monopartite NLS. For testing the specificity of detector binding or application in BiAD sensors, the mVenus fluorophore was replaced by dsRed or the C-terminal part of IFP2.0 (amino acid 133–321), respectively, by Gibson assembly.

The MBD1 MBD domain and its R44Q binding pocket mutant for DNA methylation readout were taken from.[17] For use in the dual-color BiAD sensor, the C-terminal part of mVenus was exchanged to the C-terminal part of IFP2.0. The CBX7 chromodomain (aa 1–76 of human CBX7, accession number NP_783640.1) for H3K27me3 readout was cloned as single or double domain as described above. The W35A point mutation was introduced by site-directed mutagenesis PCR.[59] For H3K36me2/3 detection, the PWWP domain of human DNMT3A1 (amino acid 283–425, accession number NP_783328.1)[34] was cloned as single or double domain into the mVenus-C1 expression vector as described above. The K299E, R366E and D333A point mutations were introduced by site-directed mutagenesis PCR.[59] For H3K9me2/3 readout, the previously used mouse HP1β chromodomain (amino acid 17–76, accession number NP_031648)[17] and the W42A binding pocket mutant were taken and cloned as single or double domain as described above.

Transfections

For transient transfections, HEK293 or NIH3T3 cells were seeded either on glass cover slips in a 6-well plate or, for live-cell imaging, in a 35 mm glass bottom Fluorodish (World Precision Instruments) at a density of 200,000 cells per well one day before transfection. Right before transfection, the medium was exchanged with 1.5 mL fresh culture medium. For transfections with the fluorescent protein IFP2.0, the medium was supplemented with 30 nM biliverdin which serves as the chromophore. The plasmid amounts for the various transient transfection reactions are given in Table S4. Each well was transfected with 1.5 μg total DNA using FuGENE (Promega) according to the manufacturer's instructions.

Fixation and antibody staining

Cells were fixed 24 h after transfection unless otherwise indicated. Before fixation, the cells were washed three times for 5 min with PBS with $CaCl_2$ and $MgCl_2$ (PBS+) to prevent detachment of the cells during the washing steps. Fixation was conducted for 10 min with 4% paraformaldehyde. Afterward, cells were washed three times for 5 min each with PBS+ and either mounted directly onto glass slides with a drop of fluoromount G with or without DAPI (Invitrogen) or antibody staining was performed.

For antibody staining, cells were permeabilized and blocked for 30 min at room temperature using saponin blocking buffer containing 5% (v/v) fetal bovine serum, and 0.1% (w/v) saponin (Roth) in PBS+. The cells were incubated with antibodies against the respective histone mark overnight at 4°C, whereby the antibodies were diluted in saponin antibody dilution buffer containing 1% (w/v) Albumin, Bovine (Sigma-Aldrich), and 0.1% (w/v) saponin (Roth) in PBS+. On the next day, the slides were washed three times for 5 min and then incubated with the corresponding secondary antibody diluted in saponin antibody dilution buffer for 1 h at room temperature in the dark. After three additional 5 min washing cycles with PBS+, the glass slides were mounted with a drop of fluoromount G with or without DAPI (Invitrogen). A list of primary and secondary antibodies for immunostainings is provided in Table S5.

The specificity of all primary antibodies used in this study was validated using CelluSpot arrays (Active Motif), basically as described in.[60] The α-H3K9me3 antibody showed a clear preference for H3K9me3 with only weak binding to H3K9me2 (Figure S5A). The α-H3K36me3 antibody demonstrated binding to H3K36me3, however, we observed some cross-reactivity with H4K20me3, H3K27me3 and H3K9me3 on peptide arrays (Figure S5B). During this study, two different antibodies were used for H3K27me3 immunostaining. The polyclonal α-H3K27me3 antibody (Active motif 39155) displayed high specificity toward H3K27me3 (Figure S5C). Due to the batch-to-batch variability of polyclonal antibodies, we also evaluated a monoclonal α-H3K27me2/3 antibody as an alternative (Figure S5D). Since we observed strong binding to H3K27me3 with only a weak signal for H3K27me2 in our specificity analysis, we utilized both antibodies for H3K27me3 immunostaining.

Fluorescence microscopy and image analysis

The samples were imaged using a confocal laser scanning microscope (LSM710 or LSM980 Airyscan 2, Carl Zeiss) equipped with a Plan-Apochromat 63x/1.4 Oil DIC M27 objective. The laser excitation wavelength and emission collection windows were ex. 405 nm/em. 410–479 nm for DAPI/Alexa 405, ex. 514 nm/em. 515–544 nm or em. 515–621 nm, if the 561 nm laser was not used in the experiment, for mVenus/YPet, ex. 561 nm/563–621 nm for dsRed, ex. 633 nm (LSM710)/ex. 639 nm (LSM980)/em. 638–747 nm for IFP2.0, Alexa 633 and Alexa 647 dyes. The image acquisition settings were kept constant within the same experiment. Z-stacks through the nucleus were collected with an interval of 0.5 μm (LSM710) or 0.13 μm (LSM980). Live-cell imaging was conducted at 37°C and 5% CO_2. The stacks were superimposed to gain the maximum intensity projection using the lite ZEN 3.0 SR (black edition) software. For image export, the Zen 3.0 (blue edition) software was used. The exemplary images were exported with the same brightness and contrast settings for each comparison of WT and mutant sensors.

Images were analyzed using a custom FIJI (ImageJ 1.54b)[50] macro without adjustments of contrast and brightness. Based on the YPet channel, two intensity thresholds were set manually for each cell to define the nucleus and spots as regions of interest (ROIs) (see Figure S1C). First, a low intensity threshold was applied to define the nuclear background signal. Subsequently, a second, high-intensity threshold was defined to identify the brightest YPet spots within the nucleus with a maximum of six spots per cell. Afterward, the mean intensity of the ROIs was measured in all channels. For quantitative analysis, the nuclear signal intensity was subtracted from the spot intensities for each channel. Then, the BiAD spot intensity (IFP2.0 channel) was normalized to the corresponding YPet spot intensity (Figure S1C; equation 1). Unless otherwise stated in the figure legends, the normalized BiAD signals of all spots within one cell were averaged and are represented as dot in the boxplots (Figure S1C; equation 2). Box-, swarm- and stripplots were generated using the Seaborn Python data visualization library.[52,51]

siRNA knockdown of H3K9 methyltransferases

For individual transient knockdown of the five mammalian H3K9 methyltransferases EHMT1 (GLP), EHMT2 (G9a), SETDB1, SUV39H1 and SUV39H2, 0.5x10^{6} cells were seeded in a 6 cm dish and siRNAs were transfected with RNAiMAX (Invitrogen) according to the manufacturer's instructions. The siRNAs used were purchased from ThermoFisher. A list of all siRNAs is provided in Table S6. Three days post transfection, cells were reseeded either onto glass slides for microscopy or in a 6-well cell culture dish for knockdown validation. Transfections of the dual-color BiAD sensor were carried out as described above. Cells were either fixed for microscopy or snap frozen for knockdown validation five days after siRNA transfection.

For knockdown validation, total RNA was isolated from cells using the RNeasy Plus Mini Kit (Qiagen) according to the manufacturer's instructions. Subsequently, mRNA was transcribed to cDNA and gene expression was determined by qPCR basically as described in.[61] The gene expression was calculated using the $\Delta\Delta$Ct method relative to the siRNA negative control and SDHA as housekeeping gene. All primer sequences used for knockdown validation are provided in Table S7.

DNMT1 inhibitor treatment

HEK293 cells were seeded onto glass slides and transfected as described in the previous section. The medium exchange before transfection contained 30 nM biliverdin (Sigma-Aldrich) and 10 μM DNMT1 inhibitor (GSK-3685032, MedChemExpress, dissolved in DMSO)[28] or an equal volume of DMSO as control. Cells were fixed three days after transfection. For analysis of global DNA demethylation, the genomic DNA (gDNA) was isolated using the QIAamp DNA Mini Kit (Qiagen) according to the manufacturer's instructions. Afterward, 500 ng gDNA were digested at CCGG sites with MspI (5mCpG insensitive) or HpaII (5mCpG sensitive) for 1 h at 37°C. Afterward, the digested products as well as undigested control samples were analyzed on an agarose gel (1%) stained with GelRed and imaged using a Quantum imaging system (Vilber).

For locus-specific 5mC analysis, two amplicons in the PR1 locus were designed that contained HpaII restriction sites (CCGG). If the CpG site within the HpaII recognition sequence is methylated, HpaII does not cleave the DNA and the qPCR template remains intact. DNA methylation at these sites was analyzed by qPCR conducted basically as described in[61] using undigested and HpaII digested genomic DNA (5–20 ng) from three biological replicates isolated from DNMT1i and DMSO treated cells. Afterward, the template quantities were calculated using a standard curve. Variations in DNA amounts between samples were normalized using a control amplicon in the PR1 locus, which does not contain HpaII restriction sites. Primer sequences used for qPCR are provided in Table S7. Subsequently, HpaII digested samples from DNMT1i and DMSO treated cells were normalized to the respective undigested gDNA to identify the relative methylation levels.

QUANTIFICATION AND STATISTICAL ANALYSIS

Microscopy images were quantified using a custom FIJI (ImageJ 1.54b) macro (https://doi.org/10.6084/m9.figshare.23592894) and Microsoft Excel 2016 as described in "fluorescence microscopy and image analysis". Statistical analyses were conducted using Microsoft Excel 2016 and GraphPad Prism 5. All details regarding statistical analysis are provided the respective figure legends and figures, including numbers of replicates for each experiment, statistical tests used and the obtained p values. The boxplots depict the median of the data as horizontal line with the box drawn from the first quartile to the third quartile and whiskers extending to 1.5 times the interquartile range.

Cell Reports Methods, Volume *4*

Supplemental information

Modular dual-color BiAD sensors for locus-specific readout of epigenome modifications in single cells

Anja R. Köhler, Johannes Haußer, Annika Harsch, Steffen Bernhardt, Lilia Häußermann, Lisa-Marie Brenner, Cristiana Lungu, Monilola A. Olayioye, Pavel Bashtrykov, and Albert Jeltsch

Supplemental Figures

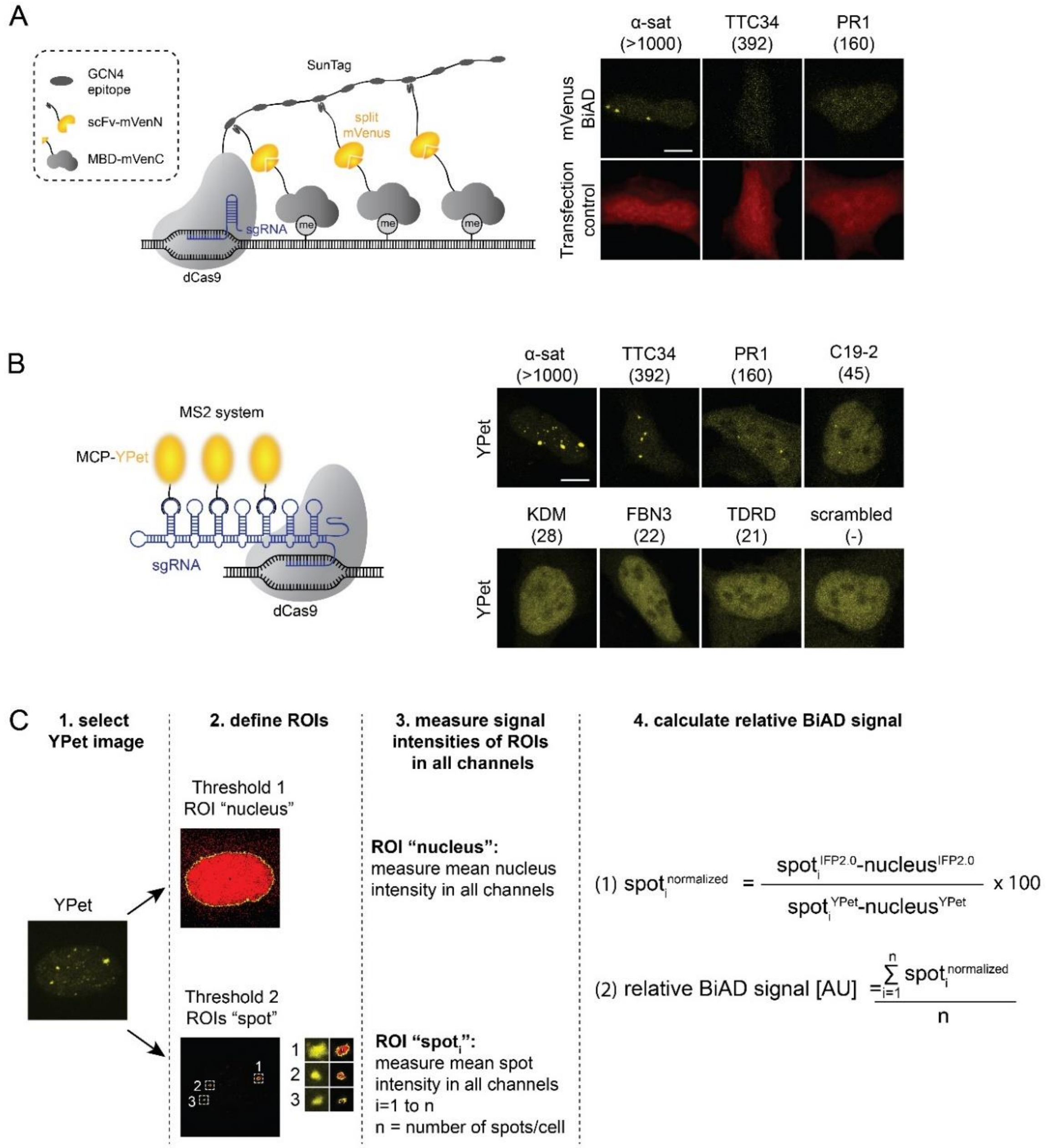

Figure S1. Development of BiAD sensors with enhanced sensitivity at repeats with lower local copy number, related to STAR Methods. A) Amplification of the BiAD signal by recruiting multiple detector modules via a 10xSunTag fused to dCas9. Exemplary fluorescence microscopy images of fixed HEK293 cells transfected with the BiAD sensor plasmids. **B)** Evaluation of the MS2-based CRISPR-Sirius imaging system in fixed HEK293 cells at different target loci. **C)** Quantitative analysis of the dual-color BiAD microscopy images using ImageJ. Two user defined thresholds are set, the first to define the nucleus and the second to define target spots in the YPet channel. The average nuclear background is subtracted from mean spot intensity for YPet and IFP2.0 channels. Then, the obtained IFP2.0 BiAD signal is normalized to the YPet marker signal. Finally, the relative BiAD signal for each cell is calculated as the mean of all normalized spots within this cell.

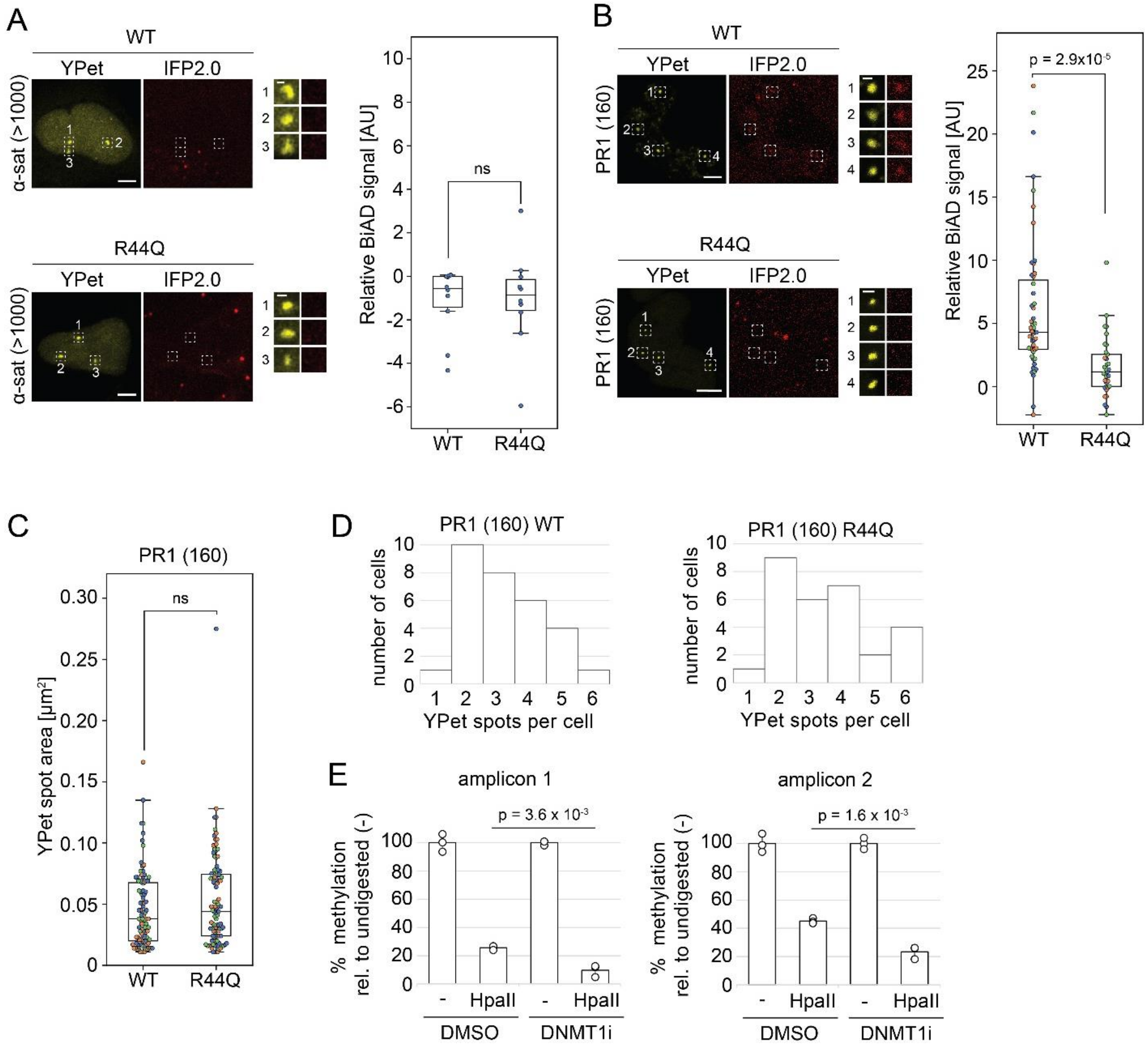

Figure S2. Additional validation of BiAD sensors for DNA methylation readout, related to Figure 1 and Figure 2. A) HEK293 cells were transfected with only four components of the dual-color BiAD sensors for 5mC detection at α-satellites (>1000 repeats), lacking the scFv-IFP2.0N expressing plasmid. Exemplary fluorescence microscopy images of fixed cells showing very low IFP2.0 (BiAD) signals and no co-localization between YPet (marker) and residual BiAD signal for wildtype and binding-deficient R44Q detector. **B)** Exemplary live-cell fluorescence microscopy images of HEK293 cells transfected with all components of the dual-color BiAD sensor for 5mC detection at the PR1 locus (160 repeats) showing the co-localization of the marker (YPet) and BiAD signal (IFP2.0) for the wildtype detector, but not for the binding-deficient R44Q mutant. **A-B)** Box plots show the relative BiAD signals. Each dot represents the mean relative BiAD signal of all spots within a single cell. Scale bar is 5 µm and 1 µm for the magnified images. **C-D)** Image analysis related to Figure 1D showing **C)** YPet spot area and **D)** spot count per nucleus for HEK293 cells transfected with all components of the dual-color BiAD sensor for 5mC detection with either the wildtype MBD1 detector (WT) or a 5mC binding-deficient mutant (R44Q). **E)** Exemplary validation of genomic DNA demethylation at the PR1 locus by DNMT1 inhibitor treatment. Genomic DNA was isolated from DNMT1i/DMSO-treated cells and digested at CCGG sites with HpaII (5mCpG sensitive). qPCR on digested and undigested (-) genomic DNA showing reduced 5mC at two amplicons within the PR1 locus for cells treated with DNMT1i compared to mock-treated cells (DMSO). Bar diagrams display the mean of three independent experiments (indicated as dots) ± SD. Significances were determined by a two-tailed, unpaired t-test. The p-values are indicated in the boxplots and bar diagrams, "ns" indicates a non-significant p-value (p>0.05).

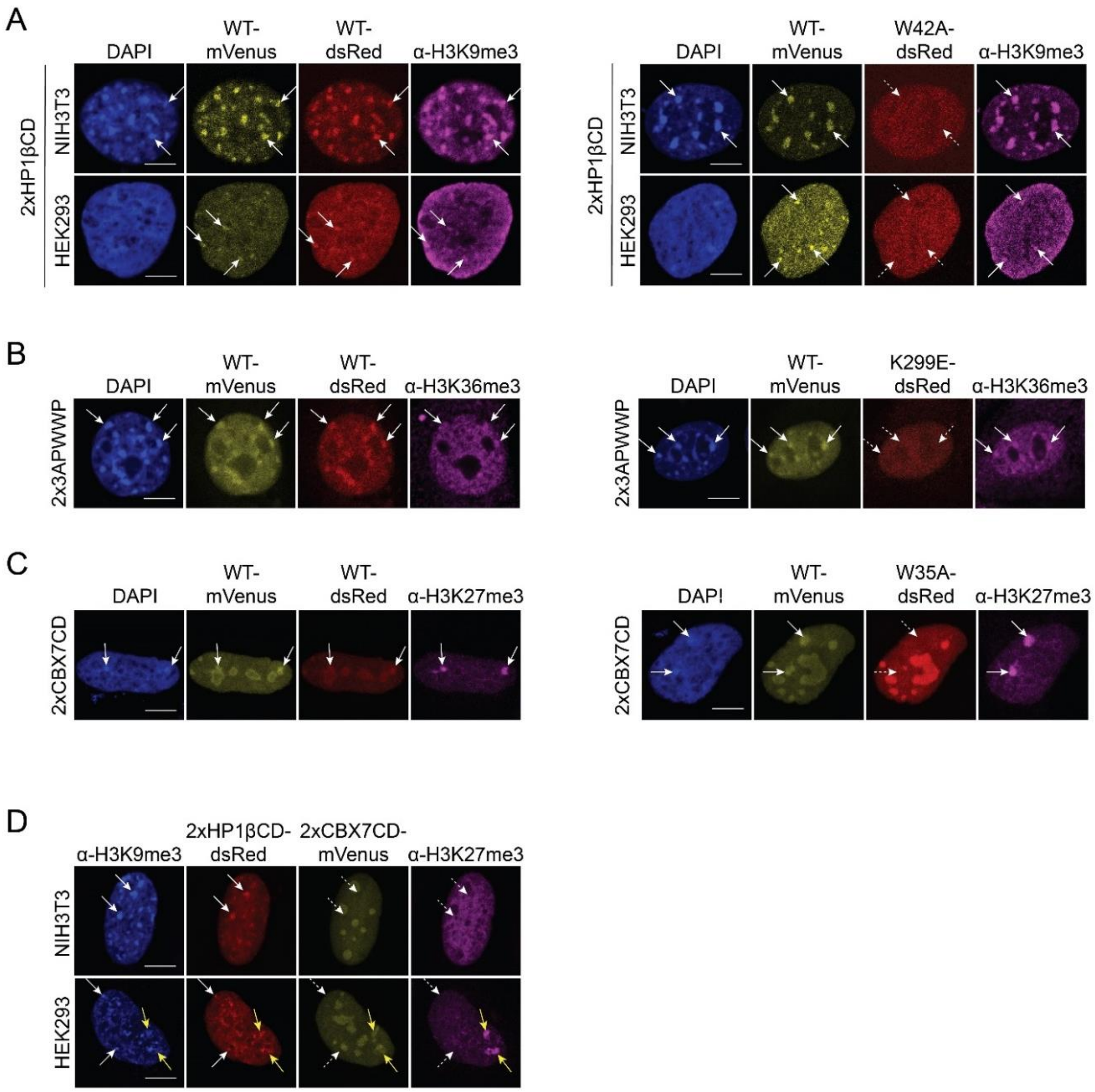

Figure S3. Validation of double reader domains as BiAD detector modules, related to Figure 3. A) Co-transfection of mVenus/dsRed-fused 2xHP1βCD WT and W42A detector modules in NIH3T3 and HEK293 cells combined with α-H3K9me3 and DAPI staining. **B)** Cotransfection of mVenus/dsRed-fused 2x3APWWP WT and K299E detector modules in NIH3T3 cells combined with α-H3K36me3 and DAPI staining. **C)** Cotransfection of mVenus/dsRed-fused 2xCBX7CD WT and W35A detector modules in HEK293 cells combined with α-H3K27me3 and DAPI staining. The co-localization of the detector with the antibody staining and DAPI are exemplarily indicated by arrows in panels A-C. Dotted arrows indicate missing co-localization of the respective binding pocket mutant. **D)** Cotransfection of 2xHP1βCD-dsRed and 2xCBX7CD-mVenus detector modules in NIH3T3 and HEK293 cells combined with α-H3K9me3 and α-H3K27me3 staining. Co-localization of 2xHP1βCD-dsRed and H3K9me3 are exemplarily indicated with arrows. Dotted arrows mark missing co-localization of 2xCBX7CD-mVenus with H3K9me3. Yellow arrows indicate the Xi in HEK293 with co-localization in all channels. All panels show images of fixed cells.

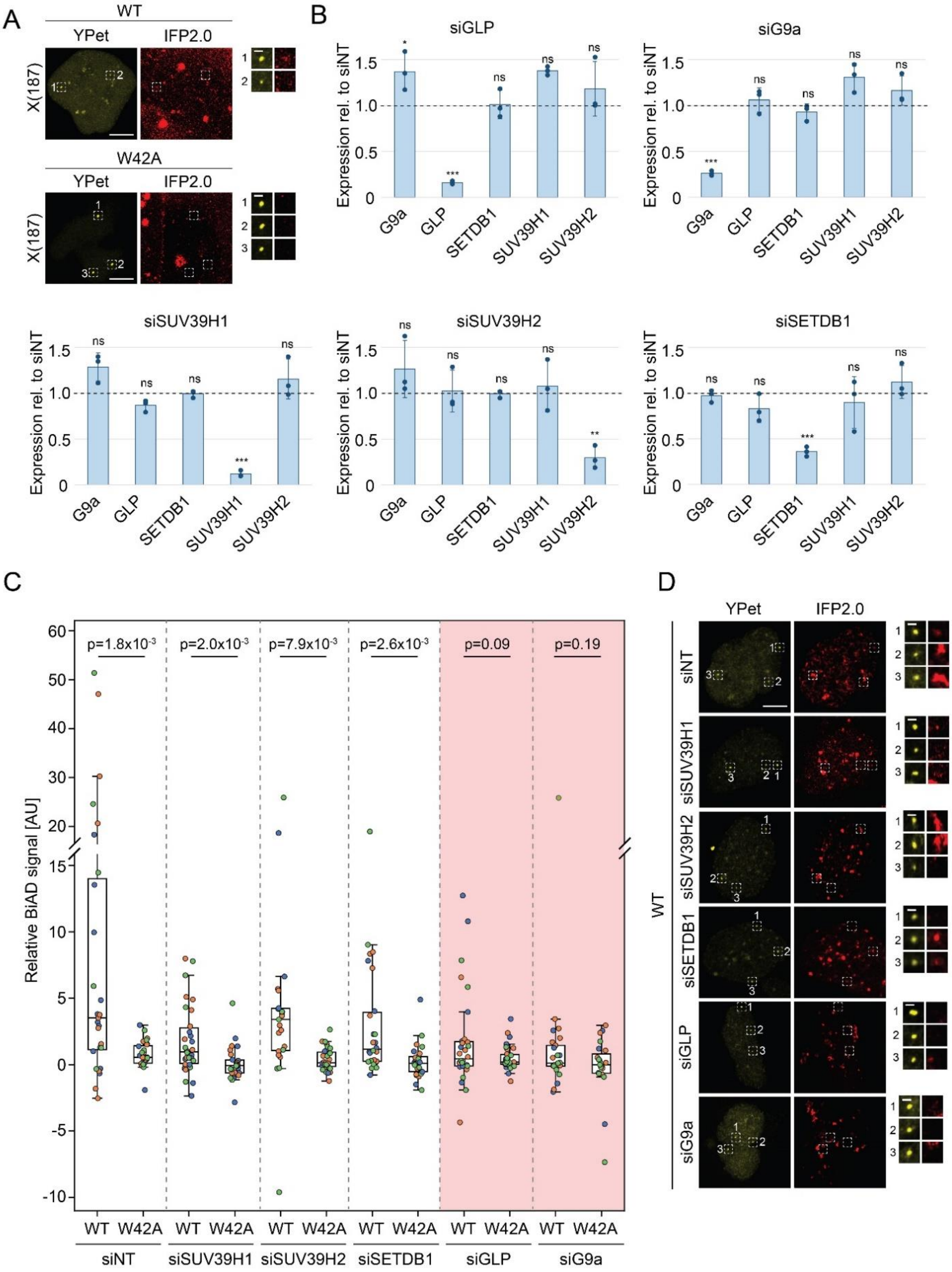

A
WT
YPet
IFP2.0
X(187)
W42A
B
siGLP
siG9a
siSUV39H1
siSUV39H2
siSETDB1
Expression rel. to siNT
G9a
GLP
SETDB1
SUV39H1
SUV39H2
ns
C
Relative BiAD signal [AU]
p=1.8x10^-3
p=2.0x10^-3
p=7.9x10^-3
p=2.6x10^-3
p=0.09
p=0.19
WT W42A
siNT
siSUV39H1
siSUV39H2
siSETDB1
siGLP
siG9a
D
YPet
IFP2.0
WT
siNT
siSUV39H1
siSUV39H2
siSETDB1
siGLP
siG9a

Figure S4. H3K9 PKMT siRNA knockdown validation and investigation of H3K9me2/3 changes at the X(187) locus, related to Figure 6. A) Exemplary live cell fluorescence microscopy images related to Figure 6A. **B)** RT-qPCR knockdown validation using siRNAs targeting the human H3K9 PKMTs SUV39H1, SUV39H2, SETDB1, GLP and G9a relative to a control siRNA (siNT). Bar diagrams illustrate the PKMT expression relative to control siRNA (siNT) five days after siRNA transfection showing the mean of three independent experiments (indicated as dots) ± SD. Significance was determined by one-way ANOVA and Dunett post-test using siNT as the control group; * $p \leq 0.05$, ** $p \leq 0.01$, *** $p \leq 0.001$, “ns” $p > 0.05$. **C)** Four days after siRNA transfection, HEK293 cells were transfected with all components of the dual-color BiAD sensor for H3K9me2/3 detection at the X(187) locus using either the wildtype 2xHP1βCD detector (WT) or a binding-deficient mutant (W42A) as negative control. Boxplots show the relative BiAD signal at the X(187) locus in cells upon knockdown of different H3K9 PKMTs. Each dot represents the mean relative BiAD signal of one cell. Data were obtained in three independent experiments (depicted in orange, blue and green). Significance was determined via a two-tailed, unpaired t-test. The p-values are indicated in each boxplot and shown in Figure 6E. **D)** Exemplary fluorescence microscopy images of fixed cells transfected with WT detector from panel B). Scale bar is 5 µm and 1 µm for the magnified images.

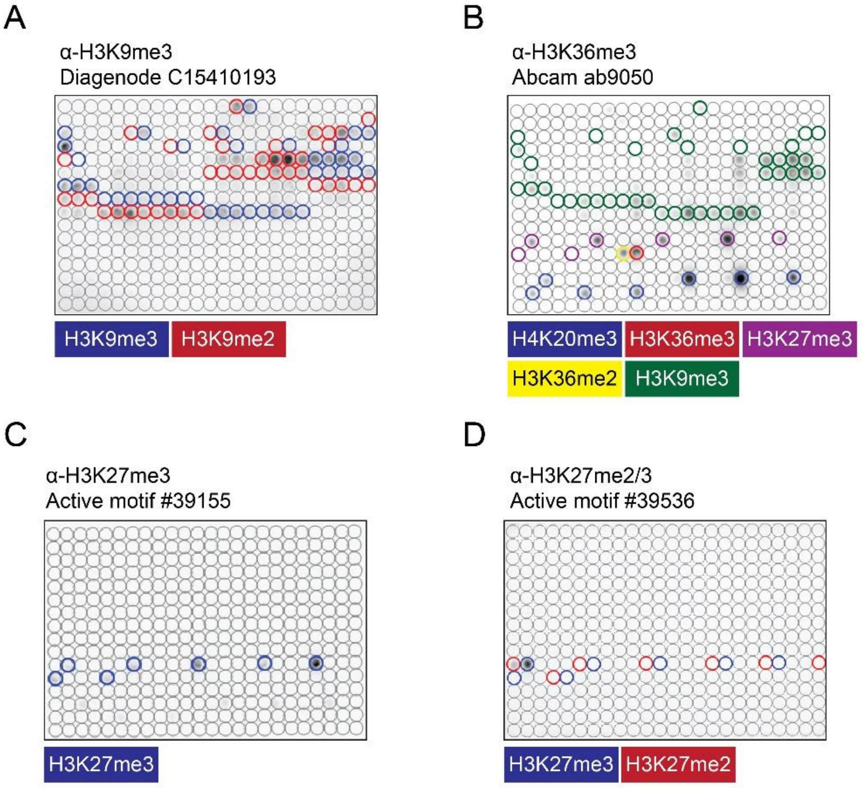

Figure S5. Antibody validation on Celluspots™ peptide arrays, related to STAR Methods. A) α-H3K9me3 (Diagenode C15410193). **B)** α-H3K36me3 (Abcam ab9050). **C)** α-H3K27me3 (Active motif #39155). **D)** α-H3K27me2/3 (Active motif #39536). Relevant peptide spots are annotated below each array.

Supplemental Tables

Table S1. Selection of target loci in the human genome that could be analyzed with the BiAD sensors described here, related to STAR Methods. The loci contain 45 or more local repeats and they are associated with a gene as indicated.

Repetitive sequence (5'-3')	Repeats	Genomic region (hg19)	Gene
CCCGTGTCTCTGCTC	59	Chr1:1223580-1225633	SCNN1D
TTAGGGTCACGGTGG	83	Chr1:2052916-2055861	PRKCZ
TCACACTCTCCCTGG	45	Chr1:227143014-227144982	COQ8A
CCAGGGAAAGCCATC	79	Chr1:246137768-246140373	SMYD3
CCACGCTCCCTAGAT	70	Chr1:246683178-246685393	LINCO1743
CCACAGGTGAGCATC	392	Chr1:2581275-2634211	TTC34
CAGAGAGAAGGGTGG	57	Chr2:1008176-1010495	SNTG2
CACTCGGGTGCAGGG	56	Chr2:1101637-1104810	SNTG2
CCTTACAGTCCTTTG	46	Chr2:1107429-1111487	SNTG2
CCCTCCCGCCTCTCT	53	Chr2:112896304-112897931	FBLN7
CCCTGTCCTGCCTGC	117	Chr2:1218818-1227201	SNTG2
CCCGAGAACTGTGCT	65	Chr2:135364479-135367491	TMEM163
CCCCTGTGTGCAACC	103	Chr2:1526480-1541951	TPO
AACTCCAGGTGAAGG	55	Chr2:1706738-1708450	PXDN
GACAGTGGTGAGTGG	83	Chr2:238451302-238453899	MLPH
CCTGGTGCAGTGCTC	59	Chr2:239441454-239459820	LINCO1107
CCTCCTCCACCTCGG	56	Chr2:241847109-241848811	CROCC2
CCCCCACACTAACCC	55	Chr2:241862860-241864454	CROCC2
GGTAGGAGCCTCTGG	112	Chr2:241915551-241920737	CROCC2
GATGGGAGCCCCGGG	45	Chr2:242704672-242706929	D2HGDH
CCGCGTGATTGCCAG	66	Chr2:242948556-242952808	LINCO1237
CCCTCCATCCAGCCC	46	Chr2:3298022-3300541	EIPR1
CCGCCTCCTCCACCA	70	Chr3:126580436-126583815	CHCHD6
CCCCTCTACTCACCC	46	Chr3:133132825-133137011	BFSP2
CCACACCCATACCTT	120	Chr3:195502236-195504816	MUC4
GGTGACAGGAAGCGG	100	Chr3:195505721-195515533	MUC4
CCTTAACTAGTCTGA	67	Chr3:197739385-197742639	LMLN
AAGTAAAATTGATGG	99	Chr3:77338424-77345592	ROBO2
AGGATGAGGGAAAGG	64	Chr4:184771961-184773735	STOX2
CCATCATACTTCCTA	88	Chr4:187353431-187357853	F11-AS1
CCCGATGTCCAAATA	48	Chr4:189424308-189430772	LINC01060
CCCCTACCCCATGCT	54	Chr4:662196-663594	PDE6B
GAGCAGGTGGGCGGG	54	Chr4:738471-741507	PCGF3
TGTGTTGGGGTCAGG	103	Chr4:7454523-7457556	SORCS2
CCCTGCTCTTCCCAC	107	Chr5:1022265-1026738	NKD2
CCCCAGTGAGCCCCC	93	Chr5:1070083-1073429	SLC12A7
GACAGGGACACCCGG	53	Chr5:1289185-1291897	TERT
CCAGCTCCTCCTCTA	74	Chr5:1326410-1329137	CLPTM1L
CCACAGCTTCCCATC	57	Chr5:176297770-176300222	UC5A
AGTCAGCGACTGAGG	48	Chr5:356563-359536	AHRR
CCAGTCCTGGTGGGG	98	Chr5:629494-633663	CEP72
GGTGTGACTGTGAGG	92	Chr5:648309-651530	CEP72
CCCCATCATAGTGTC	45	Chr6:157731388-157735394	TMEM242
GCATCTTTCTAGAGG	55	Chr6:168992931-168995966	SMOC2
ACAGCCAGCACAGGG	63	Chr6:169014272-169016452	SMOC2
CCTGCCGCACCTAGT	92	Chr6:3090978-3094477	RIPK1
GAGTGCTGGTTCCGG	50	Chr6:3145256-3147143	BPHL
AGTGGAGTCCTGTGG	71	Chr7:1040958-1043677	C7orf50
GGTGAATCACCATGG	78	Chr7:154451869-154455994	DPP6
CACTGAACTCGGTGG	83	Chr7:157439520-157442716	PTPRN2
CCATCTACCCATGCA	47	Chr7:157488518-157490732	PTPRN2
CCAGCACAGTTACCC	58	Chr7:157524153-157527056	PTPRN2
CACACTCCTGCAGGG	56	Chr7:157650733-157652746	PTPRN2
CCACACTCCATCTGC	54	Chr7:157850813-157853026	PTPRN2
CCCCATCTCACGCCA	49	Chr7:157939607-157941888	PTPRN2
CCACACTCTCACCAT	151	Chr7:158122661-158135328	PTPRN2
CCTGCGCACCGCCGG	53	Chr7:158217836-158220732	PTPRN2
CCCTGGAGTGGAGTC	46	Chr7:158316035-158317251	PTPRN2
GAGAAAGGAGAAAGG	81	Chr7:944997-946293	ADAP1
GAGGAGGAGGAAGGG	46	Chr7:981913-983521	ADAP1

Repetitive sequence (5'-3')	Repeats	Genomic region (hg19)	Gene
CCATGTCTAGTTCTC	97	Chr8:1184911-1191424	DLGAP2
CCGTGCGGGTCCTGA	46	Chr8:1298340-1300510	DLGAP2
CCAGAGTCGTGTATT	272	Chr8:1341179-1356081	DLGAP2
CAGGAGGAGCCAGGG	46	Chr8:140767496-140769803	TRAPPC9
AGATGTGTGCTCAGG	66	Chr8:145603817-145607540	ADCK5
CCAGTGGTTTCCATC	69	Chr8:145786505-145788291	ARHGAP39
CCTCACCTGTGTCAC	73	Chr8:1590035-1592301	DLGAP2
AGGACAGAGACGCGG	46	Chr8:625097-626540	ERICH1
CCCCCTGCACCCCCA	60	Chr8:940305-943540	DLGAP2
CCTCCACTGTGTGTG	98	Chr8:963588-967669	DLGAP2
TGTCACCGCGAGGGG	57	Chr8:983686-986261	DLGAP2
TGTGAGTGCATGTGG	45	Chr9:135252516-135254116	TTF1
ATCACACCTCCCAGG	70	Chr9:137740627-137742849	MIR
ACGGGACTACACGGG	61	Chr9:140223024-140225109	EXD3
CCCCCAGACCAGGAC	52	Chr9:140334277-140335713	ENTPD8
ACTCTGTCTGTGAGG	84	Chr9:140459676-140463065	DPH7
CCACCTGCCCTGATG	46	Chr9:140912342-140914057	CACNA1B
TGTGTGATGCTGGGG	78	Chr10:133972112-133975418	JAKMIP3
CCTGGGTTCTGTCTG	60	Chr10:134531001-134535345	INPP5A
CCCTGGATCACAGCC	48	Chr10:1582650-1585175	ADARB2
TCCCACTCCACCAGG	60	Chr10:1741356-1744133	ADARB2
AGATGAGCTTGGGGG	46	Chr11:134034437-134037905	NCAPD3
GAGCGTCTTCTGCGG	76	Chr11:1450754-1454390	BRSK2
CCCCGCTCACCCCCG	64	Chr11:400746-403054	PKP3
TCTGACCATGTCTGG	49	Chr11:410465-412766	SIGIRR
CACACATCAACATGG	68	Chr11:60866662-60869195	CD5
TATGCCTATGCCTGG	89	Chr12:1194126-1200023	ERC1
TGGGGTAGAAGGTGG	47	Chr12:122185156-122186161	TMEM120B
GGGGGTCTGTCCCGG	62	Chr12:124395812-124397056	DNAH10
CCTCATCCTCATCAC	59	Chr12:124951485-124953558	NCOR2
GAGTGAGTGGAGTGG	49	Chr12:125894105-125895755	TMEM132B
GAGTGTGTTTACTGG	46	Chr12:129130259-129133630	TMEM132C
TCCTCCATGACCGGG	52	Chr12:129572401-129574161	TMEM132D
CCAGGGGAACGGGAT	98	Chr12:130339606-130343633	TMEM132D
CCTTCCCTATACCCA	152	Chr12:132811643-132819399	GALNT9
CCTGGGCACACCCTC	65	Chr12:132879958-132882307	GALNT9
CCAGTTAGTTAGTGA	78	Chr12:133268545-133271869	PXMP2
CACCACCAGCACAGG	51	Chr12:3306816-3308072	TSPAN9
CCTGCAGCCTGCCCT	54	Chr12:3377306-3378778	TSPAN9
GACAGGGACAACTGG	62	Chr12:40876395-40885001	MUC19
CCACCTTCCACCTTC	102	Chr12:620386-622381	B4GALNT3
CCTGGTAAGCATGGA	397	Chr13:112930813-112973591	LINC01043
GGAGGACCTCTGTGG	54	Chr13:114000255-114002380	GRTP1
CCTCGCGGGGAAATC	70	Chr13:114848979-114852850	RASA3
GAATGTGAGTGACGG	60	Chr13:30131785-30133705	SLC7A1
CCCGTGGCACCCTCA	111	Chr14:105695963-105707283	BRF1
GCAGATGAGGGATGG	85	Chr15:101094498-101098864	PRKXP1
ACGGTGACAGCACGG	48	Chr16:1010255-1012281	LMF1
GGTGTCTCGGGATGG	66	Chr16:907422-909987	LMF1
CCTGAGCCTGAGTTC	74	Chr17:1020323-1022749	ABR
TACTTCCTATGAGGG	65	Chr17:103456-107807	RPH3AL
CCACCGTCAGACCTC	67	Chr17:136689-138746	RPH3AL
CCACCTCCATTGACC	61	Chr17:164188-167359	RPH3AL
ACACACCTCCACTGG	59	Chr17:26842241-26845271	FOXN1
CCCTCAGAACCTAAT	63	Chr17:478652-487334	VPS53
GGAGTTAGGGAGGGG	53	Chr17:77000014-77001370	CANT1
AGGTGATGGTGGTGG	73	Chr17:78287209-78289860	RNF213
CCGCACGGCAGCTCA	57	Chr17:78814075-78815874	RPTOR
CCTCAGTTTCCCTCA	75	Chr17:80317257-80319485	TEX19
CCGCGTGTGGGATTC	45	Chr17:80960763-80962730	B3GNTL1
CCTCCTCCTCTCCCC	45	Chr17:852048-852985	NXN
CCTTCCTCTATTGAT	55	Chr17:92037-94098	RPH3AL
TATGTGAACTGAGGG	48	Chr17:965418-968846	ABR
CCTGATTATTAACAT	67	Chr18:13673058-13676522	FAM210A
CCACACTGTGCCTTT	46	Chr18:44926826-44929361	MIR4527HG
GTGGTCAGGTGGGGG	79	Chr18:74104742-74106806	ZNF516
GAACCGAGGGAGAGG	51	Chr18:77679453-77681928	SLC66A2

Repetitive sequence (5'-3')	Repeats	Genomic region (hg19)	Gene
CCAGGTCTGGTCATT	99	Chr19:54422568-54428888	CACNG7
GTGTAATGTCCGAGG	49	Chr19:56361126-56363117	NLRP4
CCTGCTTCCCTCCTC	77	Chr19:59050388-59054262	ZBTB45
GGTCACACTGAGGGG	64	Chr20:20335249-20338937	CFAP61
TGTGCAGTTTGGGGG	49	Chr20:59847681-59850791	CDH4
CCATTTCTAGAAACA	84	Chr20:62238367-62241366	GMEB2
GGGGGCAGTGTGGGG	57	Chr20:62724838-62725982	OPRL1
CCCTCCCTGGCTTCT	49	Chr20:62804793-62807841	MYT1
CCTGCTGAGGGAGAT	59	Chr21:45001698-45004129	HSF2BP
TGTGGAGGGGTGTGG	56	Chr21:45815540-45816849	TRPM2
CCCTGCACCAACACA	63	Chr22:44508553-44511878	PARVB
CCTGTGTGTAGGTTC	46	Chr22:47010646-47013453	GRAMD4
CCTCCAGTCAGTGCC	72	Chr22:50782053-50784952	PPP6R2
CGCGGGGTGAGCGGG	93	ChrX:1484145-1486728	IL3RA
AGAGAGATGGAGAGG	63	ChrX:2194791-2200434	DHRSX
CCAACGGGAGGCGGG	45	ChrX:303018-305087	PPP2R3B
CGCGGGGTGAGCGGG	93	ChrY:1434145-1436728	IL3RA
AGAGAGATGGAGAGG	63	ChrY:2144791-2150434	DHRSX
CCAACGGGAGGCGGG	45	ChrY:253018-255087	PPP2R3B

Table S2. Plasmids deposited with Addgene, related to STAR Methods.

Plasmid	Addgene ID
dCas9-10xSunTag(22aa)	215745
scFv-IFP2.0N	215746
MCP-YPet	215747
MBD-IFP2.0C	215748
MBD_R44Q-IFP2.0C	215749
2xHP1bCD-IFP2.0C	215750
2xHP1bCD_W42A-IFP2.0C	215751
2x3APWWP-IFP2.0C	215752
2x3APWWP_K299E_D333A-IFP2.0C	215753
2xCBX7CD-IFP2.0C	215754
2xCBX7CD_W35A-IFP2.0C	215755

Table S3. Compilation of genomic targets, their local copy numbers and sgRNAs used in this study, related to STAR Methods.

Target locus	Local copies	Chromosome	sgRNA sequence
α-satellites	> 1000	9	TGGAATGGAATGGAATGGAA
TTC34	392	1	GATGCTCACCTG
X(187)	187	X	CAAGGCAAGGCA
PR1	160	19	GTGACAGTGAAC
C19-2	45	19	AGCAGATGTAGG
KDM	28	19	CACTCCGATAAA
FBN3	22	19	ATCCCTCCAACC
TDRD	21	19	GACTGGCTGATG
scrambled	-	-	GAACAGTCGCGTTTGCGACT

Table S4. Plasmids used for transient transfection of BiAD sensors and detector validation, related to STAR Methods.

Plasmid	Amount [ng]
sgRNA	400
dCas9-SunTag	100
scFv-IFP2.0N/mVenusN	300
Detector-IFP2.0C/mVenusC	300
MCP-YPet/NLS-mRuby2-C1 (transfection control)	400
Detector-mVenus	200
Detector-dsRed (optional)	200
pcDNA4.1 (filler plasmid for detector validation)	add 1500

Table S5. Antibodies used in this study, related to STAR Methods.

Antibody	Company	Catalog No	Dilution
α-H3K36me3	Abcam	ab9050	1:500
α-H3K27me3	Active Motif	39155	1:500
α-H3K27me2/3	Active Motif	39536	1:750
α-H3K9me3	Diagenode	C15410193	1:500
goat α-rabbit IgG Alexa Fluor™ 405	Invitrogen	A-48258	1:750
goat α-mouse IgG Alexa Fluor™ 405	Invitrogen	A-31553	1:750
goat α-mouse IgG Alexa Fluor™ 594	Invitrogen	A-11005	1:2000
goat α-mouse IgG Alexa Fluor™ 633	Invitrogen	A-21052	1:2000
goat α-rabbit IgG Alexa Fluor™ 647	Invitrogen	A-32733	1:2000

Table S6. siRNAs used in this study, related to Figure 6.

Knockdown target	Ambion silencer select assay ID
siNT (negative control)	#4390843
GLP (EHMT1)	s36392
G9a (EHMT2)	s21469
SETDB1	s19112
SUV39H1	s13658
SUV39H2	s36183

Table S7. Primer sequences used for RT-qPCR siRNA knockdown validation and 5mC-sensitive qPCR, related to Figure 2 and Figure 6.

H3K9 methyltransferase	forward primer (5' to 3')	reverse primer (5' to 3')
GLP (EHMT1)	CAGGACTTCCAAGGAGAGCA	ACTCAGGTCAGACTCGTCAC
G9a (EHMT2)	AGTGATGATGTCCACTCACTGGGA	AGAGACTGAAGTCATCACCCACCA
SETDB1	GCCTACAGCAAGGAACGTATCC	GTTGATGGCAGGCACACTTGGA
SUV39H1	GCCCAAATCGTGTGGTACAG	CCTGACGGTCGTAGATCTGG
SUV39H2	ATTGATAACCTCGATACTCGTCTT	TCTCCAGAACCTTTCATTTGATAA
SDHA (housekeeping gene)	TGGGAACAAGAGGGCATCTG	CCACCACTGCATCAAATTCAT
Amplicon 1	ACCATCCACCACTGGTTCAC	CCCACTGTGGTGGTAGAGTA
Amplicon 2	AAGCTGTTCCGTGTTAGCC	TGAAGAGGTTCCATCGTGGT
Control amplicon	CCCACTCGTTCTCCGAAACA	TGGATGGCAGTGTTGTGGTT
